# Path planning for mobile robots by fusing ant colony optimization and dynamic window approach

**DOI:** 10.1371/journal.pone.0340336

**Published:** 2026-07-27

**Authors:** Tengyan Li, Shuaishuai Cui, Xiaming Cui, Yaqi Wang, Guozhu Song

**Affiliations:** College of Software, Shanxi Agricultural University, Taigu, China; Hamad Bin Khalifa University College of Science and Engineering, QATAR

## Abstract

To address the problems of low global optimization efficiency and insufficient safety in local obstacle avoidance when mobile robots perform path planning in dynamic and complex environments, this paper proposes a path planning method fusing the ant colony optimization (ACO) and dynamic window approach (DWA), namely the ACO-DWA-DPP algorithm. Firstly, the environment is modeled using a 2D grid map. A potential field force-based heuristic function is introduced to optimize the guidance of path search, and a pheromone reward-punishment strategy and an adaptive evaporation mechanism are designed to improve the algorithm’s convergence speed and global optimization capability. Then, the planned path is subjected to secondary optimization, where redundant turning points are eliminated through connectivity checks to reduce the path length. Secondly, a dynamic collision risk coefficient is incorporated into the dynamic window approach, and the local obstacle avoidance evaluation function is improved to enhance the algorithm’s real-time response capability to dynamic obstacles. Simulation results show that, compared with the traditional ant colony optimization, the improved algorithm reduces the final converged longest path length by 41.26% ~ 48.28%, shortens the shortest path by 10.68% ~ 12.64%, decreases the number of iterations by 83.37% ~ 89.51%, and reduces the number of turning points by 66.94% ~ 81.37%. Moreover, the fused algorithm demonstrates the capability to respond to unknown obstacles in real time within the simulation environment, successfully avoiding them and meeting the requirements for the safe driving of mobile robots. The fused algorithm achieves an effective combination of global path optimization and local dynamic obstacle avoidance, providing a feasible solution for mobile robot path planning in complex scenarios.

## 1 Introduction

With the rapid development of intelligent warehouse logistics [1] and unmanned driving technology [2], mobile robots have attracted significant attention due to their potential for autonomous navigation in complex dynamic environments [3]. Efficient and robust path planning constitutes a core challenge in achieving their autonomy, among which the collaborative optimization of global and local path planning algorithms is particularly crucial. However, current mainstream algorithms still face significant bottlenecks in terms of environmental adaptability, real-time performance, and path quality.

In the field of global path planning, traditional algorithms such as Dijkstra algorithm [4], A* algorithm [5], and Rapidly-exploring Random Tree (RRT) algorithm [6] have been widely applied. Among them, Ant Colony Optimization (ACO) [7] has attracted extensive attention due to its strong global optimization capability and robustness. However, ACO has inherent drawbacks including slow convergence speed, proneness to falling into local optimal solutions, and generating paths with excessive turning points. Although existing improved schemes have their respective focuses, none have systematically solved the core problems: Qu Xinhuai et al. [8] improved the convergence speed by dynamically adjusting the pheromone evaporation rate, but their heuristic function based on Euclidean distance has insufficient robustness in complex unstructured obstacle environments and failed to effectively optimize redundant inflection points of the path; Tao Yang et al. [9] introduced Particle Swarm Optimization (PSO), which enhanced the algorithm’s global search capability but significantly increased algorithm complexity and the difficulty of parameter tuning, sacrificing real-time performance, and the generated path did not consider kinematic constraints; Wu Yu et al. [10] combined Ant Colony Optimization (ACO) with Artificial Potential Field (APF) to improve the algorithm’s obstacle avoidance performance, but their static potential field parameters were difficult to adapt to dynamic obstacle environments, and the collaborative mechanism between potential field and pheromone update lacked in-depth theoretical modeling. Similar hybrid methodologies have been explored in other contexts; for instance, Orozco-Rosas et al. [11] proposed a membrane pseudo-bacterial potential field (MemPBPF) algorithm that integrates membrane computing with APF for autonomous mobile robot path planning, demonstrating improved performance in complex scenarios. To sum up, existing improved schemes for ACO still face challenges in the following aspects: the heuristic function fails to effectively integrate target orientation, direction deviation, and dynamic risk assessment; the pheromone update mechanism lacks refined rewards and punishments for path quality; path smoothness optimization is often treated as an independent post-processing step and not effectively embedded into the core search process [12].

In the field of local path planning, the Dynamic Window Approach (DWA) [12] has become a mainstream solution due to its strict adherence to the kinematic constraints of mobile robots and efficient online obstacle avoidance capability. However, the basic DWA algorithm has drawbacks such as fixed parameters in the evaluation function, lack of global guidance, and insufficient response to the acceleration of dynamic obstacles [13]. Recent improvement efforts still have limitations: Li Xinying et al. [14] introduced Reinforcement Learning (RL) to optimize evaluation weights, which enhanced the algorithm’s scenario adaptability, but training relied on a large amount of simulation data, and the computational overhead of online decision-making increased significantly, making it difficult to ensure strict real-time performance; An alternative reinforcement learning paradigm for path planning is the QAPF learning algorithm, which combines Q-learning with artificial potential fields to accelerate learning and improve path quality in both known and unknown environments [15]; Jia Qianxi et al. [16] improved the algorithm’s dynamic obstacle avoidance using the velocity obstacle model, but their assumption of constant obstacle velocity led to insufficient modeling of motion state prediction and collision risk under varying acceleration; Li Yuqing et al. [17] used the global path as the guiding target for DWA, which provided direction constraints for the algorithm, but there was a lack of closed-loop feedback between the two. When the global path becomes invalid due to dynamic obstacle avoidance, the local planner tends to fall into a local optimum or generate oscillations. In summary, the key limitations of existing DWA algorithms are as follows: the risk quantification model for dynamic obstacles is overly simplified and fails to fully consider relative acceleration factors; the collaboration with global planning is mostly one-way guidance, lacking real-time feedback of environmental perception information and a mechanism for global path re-optimization.

To address the aforementioned bottlenecks in global and local planning, this paper proposes a collaborative path planning framework that integrates the Ant Colony Optimization (ACO) and Dynamic Window Approach (DWA) (ACO-DWA-DPP). Its core innovative contributions are as follows:

First, unlike conventional hybrid planners that employ a unidirectional pipeline, this study introduces a bidirectional closed-loop feedback mechanism. The global path generated by the improved ACO algorithm guides the initialization of DWA-based local planning; meanwhile, the DWA perceives environmental changes in real time (such as unknown obstacles and path blockages) and feeds them back to the ACO layer, triggering pheromone redistribution and global path re-optimization. This architecture breaks through the limitations of traditional serial planning and achieves the collaborative evolution of global optimality and local real-time performance.

Second, at the DWA layer, a dynamic collision risk quantification model is constructed. By analyzing the relative distance, relative velocity, and acceleration between the mobile robot and obstacles, the collision time is accurately calculated. A risk coefficient is defined accordingly and embedded into the trajectory evaluation function. This represents a significant advancement over standard DWA, which typically considers only position and velocity.

Third, redundant turning points are eliminated post-convergence through connectivity detection, directly outputting a low-circuitous path that meets the kinematic constraints of mobile robots. This ensures that the smoothing process does not interfere with the iterative pheromone-guided search, preserving the efficiency of global optimization.

## 2 Traditional ant colony optimization

This chapter begins by elaborating on the two-dimensional grid mapping method used for modeling the workspace of mobile robots. Subsequently, it provides a detailed introduction to the fundamental principles and core mechanisms of the traditional ant colony optimization algorithm. This includes the pseudo-random proportional state transition rule for path search, the roulette wheel selection method for implementing this rule, and the pheromone update strategy designed to balance the algorithm’s exploration and exploitation capabilities. These elements constitute the theoretical foundation for the subsequent algorithmic improvements presented in this paper.

### 2.1 Environmental modeling

**Fig 1:**
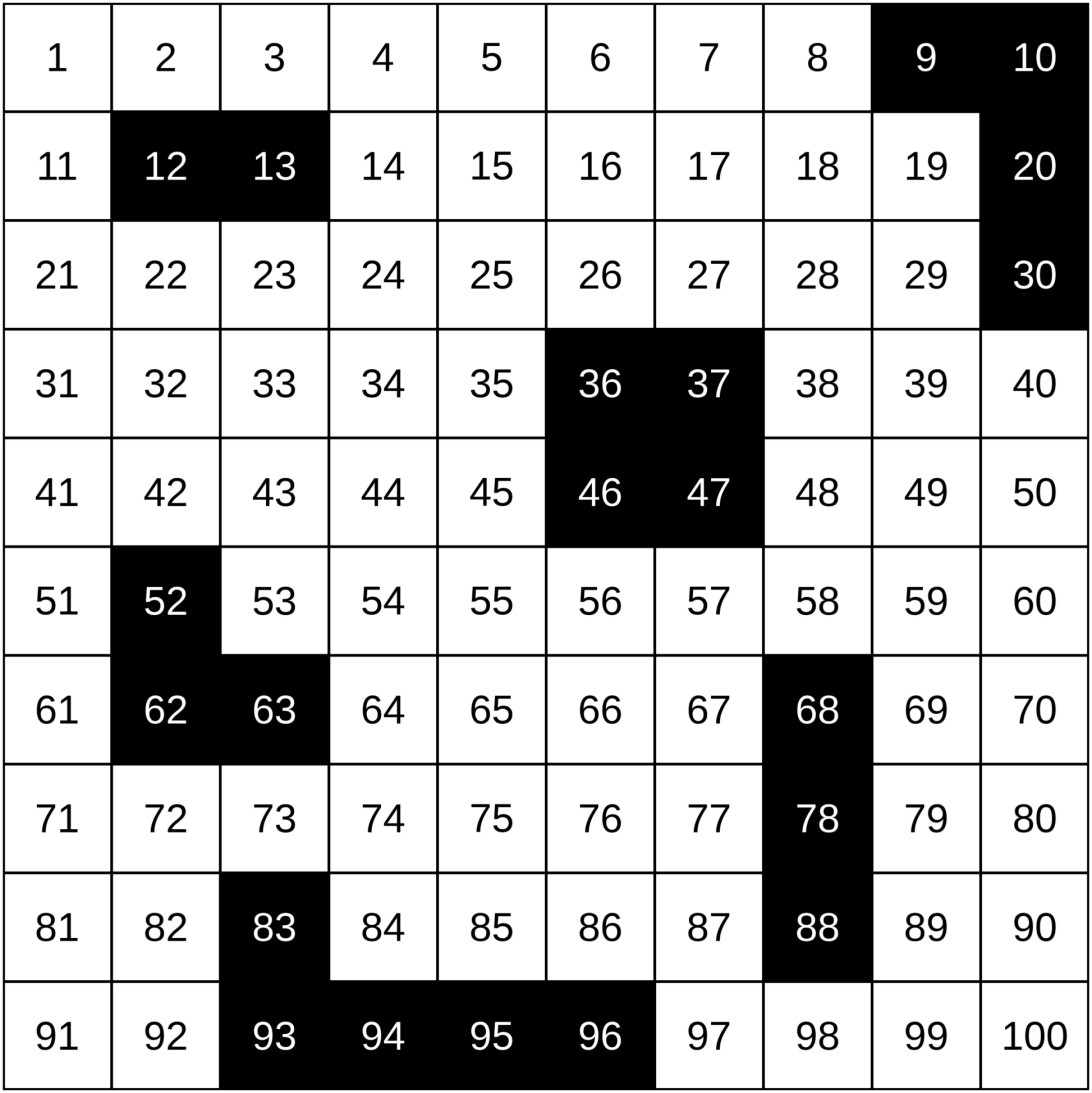
Example of a 10 × 10 2D grid map.

In this study, the operational environment of the mobile robot is represented using a 2D grid map [18]. The continuous space is discretized into a uniform grid matrix of size C × N, where each grid cell has a side length of a. In this representation, obstacle areas are assigned a value of “1” (depicted as black cells in Fig 1), while free spaces are assigned a value of “0” (shown as white cells in Fig 1). For illustrative purposes, an example of a 10 × 10 grid map is provided in Fig 1.

Grid index E is arranged in row-major order (from left to right and top to bottom), and its physical center coordinates (${\text{x}}_{\text{E}}$, ${\text{y}}_{\text{E}}$) are calculated by Equation (1):


$$\begin{array}{c} \left\{ \begin{array}{c} @lx_{E}=\;a\;\times \left(mod\;(E,N)\;-\;\text{0}.\text{5}\right)\\ y_{E}=\;a\;\times \left(C\;+\;\text{0}.\text{5}\;-\;\left\lceil E\;÷\;N\right\rceil \right) \end{array}\right. \end{array}$$ (1)

When mod(E,N)=0, the value of XE is adjusted to XE = a(N − 0.5) to mitigate boundary errors. The binary encoding scheme—where 0 denotes free space and 1 represents obstacles—enables lightweight storage and efficient map updates, thereby satisfying the computational efficiency requirements for real-time path planning in dynamic environments.

### 2.2 Traditional ant colony optimization

The Ant Colony Optimization (ACO) algorithm is a swarm intelligence method inspired by the foraging behavior of ant colonies. Specifically, it simulates the pheromone-mediated indirect communication among ants to achieve distributed optimization, utilizing a positive feedback mechanism that effectively guides the swarm to identify high-quality paths within the solution space.In essence, the algorithm operates by simulating pheromone-mediated communication. As ants move, they deposit pheromones, creating trails that influence the path selection of subsequent ants, who favor routes with stronger pheromone concentrations. As the algorithm proceeds, shorter paths are reinforced more rapidly due to the higher frequency of ant traffic, thereby enhancing their pheromone trails. This self-reinforcing process enables the colony to collectively converge upon the optimal path.

Traditional ACO algorithms employ a roulette wheel selection strategy to govern probabilistic path search, where the selection probability is a function of both pheromone concentration and heuristic factors. Nevertheless, the reliance on static parameters often creates a tension between search efficiency and convergence speed. Therefore, we proceed to systematically dissect the core elements of the conventional ACO framework—namely, the probabilistic pheromone model, the roulette wheel selection mechanism, and the pheromone update rules—to lay the theoretical groundwork for the improved algorithm presented herein.

#### 2.2.1 Probabilistic selection.

In the traditional Ant Colony Optimization (ACO) algorithm, each ant determines its movement direction using a pseudo-random proportional selection strategy. This mechanism enhances search efficiency by striking a balance between exploitation and exploration. Specifically, when ant k is located at node i at time t, its transition to the next node follows the rule described below: with a probability${\text{ q}}_{\text{0}}$(${\text{q}}_{\text{0}}\in \;$[0,1]), the ant selects the node that maximizes the product of the pheromone concentration and the heuristic function value within its neighborhood, thereby performing deterministic exploitation. Otherwise, it undertakes a random exploration strategy. This decision process is formally expressed in Equation (2):


$$\begin{array}{c} j=\left\{ \begin{array}{c} @c{arg}_{i\in A\;}max\left[{\tau_{ij}}^{\alpha }\;(t)\times {\eta_{ij}}^{\beta }(t)\right],if\;q\leq q_{\text{0}}\;\\ {P_{ij}}^{k}(t)\;,otherwise \end{array}\right. \end{array}$$ (2)

In the equation, $\tau_{\text{ij}(\text{t})}$ denotes the pheromone concentration on edge (i, j) at time t. The heuristic function $\eta_{\text{ij}}$ is defined as $\eta_{\text{ij}}$=1/${\text{d}}_{\text{ij}}$, where ${\text{d}}_{\text{ij}}$ represents the Euclidean distance between nodes i and j. The parameters α and β are the weight coefficients that control the relative importance of the pheromone trail and the heuristic information, respectively. Additionally, Ak(i) represents the set of feasible neighboring nodes for ant k when it is located at node i.

#### 2.2.2 Roulette wheel selection.

Upon adoption of the random exploration strategy (i.e., q>${\text{q}}_{\text{0}}$), the ant’s next movement is governed by a roulette wheel selection process. This process entails constructing a probability distribution over the feasible neighborhood of ant k—where each node’s selection probability is explicitly calculated—and subsequently drawing a random sample from this distribution to determine the next node. The formal definition of this probabilistic selection model is provided in Equation (3):


$$\begin{array}{c} {P_{ij}}^{k\;}=\;\left\{ \begin{array}{c} @c\frac{{\left[\tau_{ij}(t)\right]}^{\alpha }\times {\left[\eta_{ij}(t)\right]}^{\beta }\;}{\sum_{j\;\epsilon \;a_{k}}^{\infty }{\left[\tau_{ij}(t)\right]}^{\alpha }\times {\left[\eta_{ij}(t)\right]}^{\beta }},j\in a_{k}\\ \text{0},therwise \end{array}\right. \end{array}$$ (3)

In the equation, ${\text{a}}_{\text{k}}$ is the set of candidate movable grids in the neighborhood; αandβare the pheromone heuristic factor and distance heuristic factor, respectively, which govern the importance of pheromones and heuristic information in the selection process. A larger α means that ants tend to select paths with higher pheromone concentrations; a larger β means that ants tend to select nodes closer to the target point.

#### 2.2.3 Pheromone update.

The pheromone update mechanism of traditional ACO algorithms is structured around two hierarchical levels: local updates, performed in real-time, and global updates, executed iteratively. This dual-level architecture is designed to sustain a dynamic equilibrium between the ants’ explorative and exploitative behaviors.

The local real-time update mechanism is triggered upon each movement of ant k from node i to node j, where the pheromone concentration on path (i, j) is updated in accordance with Equation (4). By systematically reducing the pheromone intensity on less favorable paths throughout the search process, this mechanism serves to maintain solution diversity and prevent the algorithm from becoming trapped in locally optimal solutions:


$$\begin{array}{c} \tau_{ij}(t+\text{1})=(\text{1}-\lambda )\tau_{ij}(t)+\lambda \tau_{\text{0}} \end{array}$$ (4)

In this formulation, ρ∈(0, 1) is the local pheromone evaporation rate, controlling the attenuation of pheromone intensity over time. Meanwhile, $\tau_{\text{0}}$ represents the initial pheromone concentration assigned to each edge, ensuring a minimum level of trail information to guide ants in their initial path exploration.

During the global iterative update phase, following the completion of a full iteration by the entire ant colony, the pheromone trails on the globally optimal path are enhanced. This global update mechanism is mathematically expressed in Equation (5):


$$\begin{array}{c} \tau_{ij}(t+\text{1})=(\text{1}-\rho )\tau_{ij}(t)\;+\sum_{k\;=\;\text{1}}^{m}\Delta {\tau_{ij}}^{k}(t,t+\text{1}) \end{array}$$ (5)

In the equation,ρ ∈ (0, 1) is the global pheromone evaporation rate, representing the temporal decay of global pheromones. $\Delta {\tau_{\text{ij}}}^{\text{k}}(\text{t},\text{t}+\text{1})$ is the increment of pheromone concentration deposited by ant k on the path from node i to node j, which reinforces the pheromone level on the globally optimal path. Its calculation rule is shown in Equation (6):


$$\begin{array}{c} \Delta {\tau_{ij}}^{k}(t,t+\text{1})=\left\{ \begin{array}{c} @c\frac{\text{1}}{L_{k}},tour(i,j)\in P_{k}\\ \text{0},else \end{array}\right. \end{array}$$ (6)

In the equation, ${\text{L}}_{\text{k}}$represents the length of the globally optimal path found by ant k; ${\text{P}}_{\text{k}}$ denotes the set of edges belonging to the optimal path in the current iteration;

Although the traditional ant colony optimization algorithm demonstrates robust performance in global path planning by virtue of its positive feedback mechanism and parallel search capability, its direct application in complex dynamic environments still faces three core challenges: First, the heuristic function is overly simplistic. Relying solely on Euclidean distance can easily lead to aimless search behavior, causing the algorithm to become trapped in local optima within complex obstacle environments. Second, the pheromone update mechanism is undifferentiated. It fails to make quality-based adjustments, resulting in slow convergence speed and a waste of computational resources. Third, the generated paths contain numerous redundant turning points and do not consider the robot’s kinematic constraints, rendering the planned paths infeasible for practical execution. These issues severely restrict the applicability of traditional ACO in real-time dynamic scenarios. Therefore, this study proposes targeted improvements to ACO from the three aforementioned dimensions.

## 3 Improved ant colony optimization algorithm

To address the issues of slow convergence, susceptibility to local optima, and poor path smoothness inherent in traditional ant colony algorithms for global path planning, this chapter proposes a multi-strategy fused improved ant colony algorithm. The improvement measures primarily include four aspects: First, a multi-factor heuristic function is designed, integrating potential field force, directional deviation, and dynamic risk assessment to enhance the goal orientation of the path search. Second, a reward-punishment mechanism based on path quality is introduced to achieve differentiated regulation of pheromone updates. Third, an adaptive strategy is employed to dynamically adjust the pheromone volatility coefficient, balancing the algorithm’s global exploration and local exploitation capabilities. Finally, a path secondary optimization method based on connectivity detection is applied to eliminate redundant turning points, significantly enhancing the smoothness and executability of the planned path.

### 3.1 Improved heuristic function

To address the limitation that the traditional heuristic function relies solely on Euclidean distance—which leads to poor goal orientation—a guidance mechanism that incorporates both environmental repulsion and target attraction is required. The artificial potential field method is selected as the theoretical foundation for enhancing the heuristic function due to its computational simplicity, strong real-time performance, and ability to effectively model target attraction [19]. Compared with complex deep reinforcement learning approaches, the potential field method does not require extensive offline training data, making it more suitable for rapid response in unknown or dynamically changing grid maps. Therefore, this paper introduces the potential field model into the heuristic function. By calculating the “virtual attraction” between the current node and the target point, the ant colony can quickly focus its search toward the goal direction in the early stage, thereby reducing blind exploration in unproductive areas.

Traditional ACO algorithms commonly employ a heuristic function based primarily on the reciprocal of Euclidean distance to the target. While simple, this single-factor approach proves inadequate in complex real-world scenarios, where it often results in aimless search behavior—leading to either premature convergence to local optima or excessive computational effort in unproductive areas. In response, this study develops an enhanced heuristic function that integrates three complementary components: potential field theory, directional deviation factors, and dynamic risk assessment. This integrated model provides a robust goal-oriented guidance mechanism. The detailed implementation procedure is described as follows:

(1) Let the coordinates of the current node i be (ix, iy) and the coordinates of the target node E be (Ex, Ey).
(2) Calculate the potential field force from node i to node E: First, compute the relative distance between the current node and the target node, as shown in Equation (7):

$$\begin{array}{c} d_{iE}=\sqrt{{\left(i_{x}-E_{x}\right)}^{\text{2}}+{\left(i_{y}-E_{y}\right)}^{\text{2}}} \end{array}$$ (7)

Calculate the attractive force from the current node to the target node:

$$\begin{array}{c} F=\mu \cdot {d_{iE}}^{\text{2}} \end{array}$$ (8)

In the equation, μ denotes the attraction constant, which regulates the relative emphasis placed on pheromone concentration and heuristic information during the node selection process of ants.
(3) The improved heuristic function is shown in Equation (9):

$$\begin{array}{c} \eta (i)=\left\{ \begin{array}{c} @c\frac{{\text{0}.\text{9}}^{F}}{{\left(d_{ij}+d_{iE}\right)}^{\text{2}}}*e^{-Risk(i)}*\left(\text{1}-\lambda *cos\theta_{iE}\right)\;,if\;i\text{ ≠}E\\ \text{1}\text{0}\text{0},if\;i\;=\;E \end{array}\right. \end{array}$$ (9)

In the equation,λ∈[0,1] adjusts the influence of directional factors on the heuristic function; θ_iE_ is the azimuth angle between node i and target point E; Risk(i) is determined based on whether the node is an obstacle area or a hazardous zone, where Risk(i) →∞ for obstacle regions and Risk(i) → 0 for free regions.

### 3.2 Pheromone reward and punishment strategy

The traditional ACO employs a uniform pheromone increment rule for all paths that successfully reach the target. This leads to insufficient differentiation between high-quality paths (short paths) and low-quality paths (long paths with numerous detours), thereby weakening the positive feedback effect of pheromones and making the algorithm prone to early stagnation or oscillation. To address this issue, this paper designs a reward-punishment strategy based on path quality grading. The core idea of this strategy is to simulate the natural selection mechanism for “shortcuts” and “detours” observed in biological ant colonies: ants that find excellent paths are given a “heavy reward” (large pheromone increment), the worst-performing ants receive a “penalty” (small pheromone increment), while ordinary paths only undergo routine updates. This mechanism aims to rapidly reinforce high-quality solutions while preserving some diversity to avoid falling into local optima, thereby seeking a better balance between exploration and exploitation.

Addressing the deficiency of the traditional ACO framework in providing differentiated feedback for paths of varying quality, this study proposes a refined pheromone update mechanism incorporating a reward-punishment strategy. This mechanism ensures that path quality is appropriately reflected in the pheromone adjustment process, thereby promoting a more favorable trade-off between global exploration and local exploitation [20]. A dynamic adjustment factor is introduced to modulate the pheromone increment, with its magnitude determined by the ratio of the ant’s path length to the current global optimum. Paths exhibiting lengths shorter than or approximating the optimum are reinforced with an increased pheromone increment; conversely, inferior paths receive only baseline updates. The proposed update formulation is detailed in Equation (10), and the corresponding dynamic adjustment factor is specified in Equation (11):


$$\begin{array}{c} {\tau_{ij}}^{\left(k+\text{1}\right)}=\left\{ \begin{array}{c} (\text{1}-\rho )*{\tau_{ij}}^{k}+factor*\left(\frac{Q}{L_{km}}+\frac{A*Q}{L_{min}}\right),best\;path\\ (\text{1}-\rho )*{\tau_{ij}}^{k}+factor*\left(\frac{Q}{L_{km}}+\frac{Q}{B*L_{max}}\right),Worst\;path\\ (\text{1}-\rho )*{\tau_{ij}}^{k}+factor*\frac{Q}{L_{km}},else \end{array}\right. \end{array}$$ (10)

$$\begin{array}{c} factor\;=\left\{ \begin{array}{c} @c\text{1}.\text{5},if\;{PL}_{km}<\;\text{0}.\text{8}*min\;kl\\ \text{1}.\text{2},if\;\text{0}.\text{8}*min\;kl\leq {PL}_{km}<min\;kl\\ \text{1},other\;wise \end{array}\right. \end{array}$$ (11)

In the equation,Q represents the intensity of pheromone deposition; factor is the adjustment factor for pheromone increment; M is the number of ants; L denotes the length of the path taken by ant m in the k-th cycle; A is the number of ants that found the shortest path in the current cycle; $L_{min}$ represents the length of the shortest path in the current cycle; B is the number of ants that found the longest path in the current cycle; and $L_{max}$ denotes the length of the longest path in the current cycle.

The proposed three-level reward and punishment framework in the improved ant colony optimization algorithm strategically balances global convergence and local exploration. It ensures that globally optimal paths receive enhanced reinforcement, establishing them as highly attractive guides for the colony’s overall search direction. Concurrently, it maintains search diversity through moderated pheromone updates for suboptimal paths and inhibitory adjustments for inferior paths. This hierarchical differentiation mitigates the risks of both undifferentiated path reinforcement and elitist over-concentration, effectively preventing premature convergence to local optima. The mechanism thus achieves a synergistic balance between exploratory breadth and exploitative depth, leveraging discovered high-quality paths while preserving the capacity to identify superior alternatives.

### 3.3 Adaptive adjustment strategy

A inherent limitation of the traditional ant colony optimization algorithm lies in its use of a constant pheromone evaporation coefficient ρ, which often leads to suboptimal pheromone distribution and disrupts the delicate balance between exploration and exploitation. To remedy this deficiency, this study proposes an adaptive adjustment mechanism that transforms the evaporation coefficient from a fixed parameter into a dynamically varying function of the iteration number. The mathematical expression for this improved coefficient is provided in Equation (12):


$$\begin{array}{c} \rho =\rho_{\text{1}}*\left(\text{1}+e^{\frac{k}{K}}\right)*\left(\rho_{max}-\rho_{min}\right)*\left(\text{1}+\text{0}.\text{1}*(rand\;-\;\text{0}.\text{5})\right) \end{array}$$ (12)

In this study, the adaptive pheromone evaporation coefficient was constrained within the range of $\rho_{\text{min}}$ = 0.1 and ${\;\rho }_{\text{max}}$ = 0.9.

In this formulation, k indicates the current iteration number, and k is the predefined maximum number of iterations. The initial evaporation coefficient $\rho_{\text{1}}$ is assigned a constant value of 0.6. Additionally, rand is a stochastic function that returns a uniformly distributed random number in the range[0,1].

The proposed adaptive adjustment strategy offers the following advantages:

(1) Dynamic adaptation with flexible regulation. By replacing the fixed evaporation coefficient with an iteration-dependent variable, the improved mechanism dynamically adjusts the pheromone decay rate throughout the optimization process. This enables the algorithm to adapt its search behavior to varying problem characteristics and optimization requirements, thereby enhancing its overall adaptability and performance across different scenarios.
(2) Enhanced robustness through multi-dimensional guarantees. The stochastic component integrated into the evaporation coefficient introduces controlled randomness that helps the algorithm escape local optima, while its dynamic nature accommodates variations in map structures and initial conditions. This dual mechanism—combining random perturbation for escaping local traps with adaptive adjustment for environmental differences—effectively mitigates issues such as premature convergence and slow convergence, ensuring both efficiency and reliability in complex optimization scenarios.

### 3.4 Path secondary optimization

The aforementioned improvements primarily optimize the search efficiency and global optimality of the ACO. However, the generated paths still consist of discrete grid points and contain numerous jagged turns, which do not comply with the mobile robot’s minimum turning radius and continuous motion constraints. If these paths were directly used for local tracking by the Dynamic Window Approach (DWA), they would lead to frequent acceleration/deceleration and oscillations. Therefore, post-processing optimization of the original path is necessary. The advantage of the secondary optimization method based on connectivity detection proposed in this paper lies in its direct geometric abstraction of the path at the topological level: by removing redundant nodes that do not affect connectivity, the path is simplified into a polyline segment composed of key waypoints. This not only preserves the guiding function of the global path but also significantly reduces the tracking burden on the DWA local planner, thereby improving overall motion smoothness [21].

**Fig 2:**
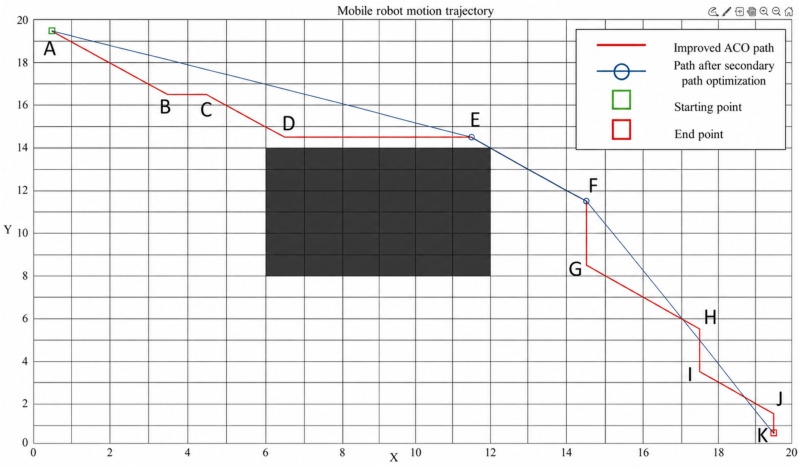
Schematic of path secondary optimization processing.

To avoid introducing additional computational burden during the iterative optimization process, the proposed path smoothing procedure based on topological connectivity is applied only once, following the convergence of the improved ACO algorithm. This ensures that the pheromone-guided search remains unaffected by post-processing, while still benefiting from a streamlined, kinematically feasible trajectory. To overcome the limitations of conventional path smoothing post-processing in traditional ACO algorithms, this paper proposes an optimization mechanism based on topological connectivity criteria. This approach iteratively prunes redundant nodes and simplifies the temporal path topology, as illustrated in Fig 2:

The topological smoothing procedure depicted in Fig 2 is executed through iterative node elimination. Commencing from the start node A, the algorithm assesses the feasibility of direct connections to downstream nodes while bypassing intermediate points. The initial evaluation confirms that a direct A–C connection is obstacle-free, permitting the removal of node B. Subsequent iterations enable the elimination of nodes C and D, establishing a direct path from A to E. Upon reaching node F, a direct connection from A is tested but found to intersect with obstacles, rendering it infeasible. Node E is therefore retained as the new reference point, and the elimination process continues recursively following the same connectivity criteria until the target node K is reached.

The obstacle collision detection mechanism in this study is founded on the geometric intersection analysis between line segments and rectangular obstacles. Path validity is assessed by examining each constituent segment for intersection with obstacle boundaries—any such intersection renders the path infeasible due to obstacle penetration. For pairwise segment intersection testing, we employ the standard cross-product orientation method. Consider two line segments AB and CD, where A, B and C, D represent their respective endpoints. The orientation function is defined in Equation (13):


$$\begin{array}{c} \text{cross}(A,B,C)=(x_{B}-x_{A})(y_{C}-y_{A})-(y_{B}-y_{A})(x_{C}-x_{A}) \end{array}$$ (13)

In the equation, A, B, C, and D denote the endpoints of two line segments. The function cross(·) is used to calculate the orientation relationship between three points.

Based on the four cross-product values, the intersection condition of the two bounded line segments can be determined as follows in Equation(14):


$$\begin{array}{c} \begin{array}{cc} o_{\text{1}} & =\text{cross}(A,B,C)\;\;o_{\text{2}}=\text{cross}(A,B,D)\\ o_{\text{3}} & =\text{cross}(C,D,A)\;\;o_{\text{4}}=\text{cross}(C,D,B) \end{array} \end{array}$$

$$\begin{array}{c} AB\cap CD\text{≠}\emptyset ⟺o_{\text{1}}o_{\text{2}}\leq \text{0}\wedge o_{\text{3}}o_{\text{4}}\leq \text{0}\wedge B_{AB,CD}=\text{1} \end{array}$$ (14)

where $B_{AB,CD}=\text{1}$ indicates that the projections of line segments AB and CD overlap in both the x and y directions.

The innovation of this method lies in its selective pruning strategy: redundant turning points are removed by evaluating the connectivity between adjacent path nodes, rather than indiscriminately optimizing all points along the path. This connectivity-driven approach preserves path validity while achieving a more concise path structure, resulting in shorter path lengths and enhanced planning efficiency.

**Fig 3:**
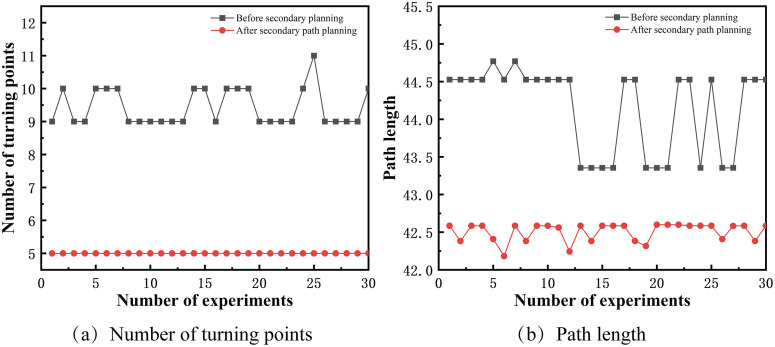
Comparison of path secondary optimization before and after.

The impact of the proposed secondary optimization mechanism is visually presented in Fig 3, which compares key path metrics before and after optimization. The following improvements are evident:

(1) Quantitative reduction in path turning points. The optimization process effectively prunes redundant and non-critical nodes, yielding a substantial decrease in the number of turning points. This structural simplification translates into reduced computational requirements, enhanced algorithmic efficiency, and improved real-time responsiveness, while simultaneously contributing to greater accuracy in the final path solution.
(2) Measurable path length minimization. A distinct contraction in path length is observable following secondary optimization, as illustrated in the figure. This reduction confirms that the proposed strategy successfully eliminates circuitous segments and unnecessary directional changes, resulting in a more streamlined and efficient path that better satisfies the planning objectives.

## 4 Basic dynamic window approach

The Dynamic Window Approach (DWA) is a trajectory planning algorithm grounded in the mobile robot’s kinematic constraints. It systematically samples admissible velocity combinations (v, w) from the velocity space, evaluates the resulting local trajectories, and selects the optimal candidate based on an objective function. The fundamental steps of this process are described below:

### 4.1 Mobile robot motion model

**Fig 4:**
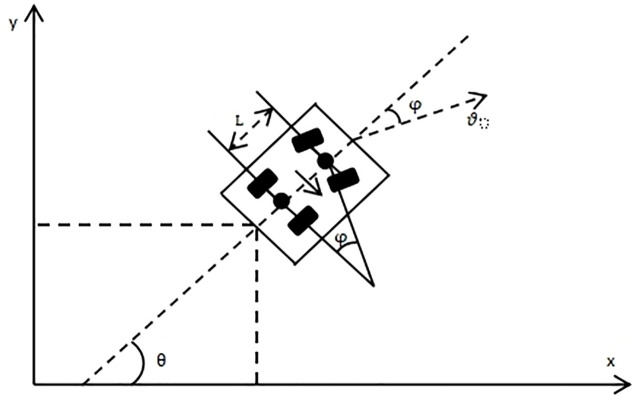
Simplified model of ackermann mobile robot.

Steering architectures in commercial mobile robot platforms generally fall into four distinct categories: differential drive (both two-wheel and four-wheel variants), Ackermann steering, and omnidirectional drive. This work specifically examines the kinematic model of robots utilizing the Ackermann steering mechanism. Through precise control of the coupling between front wheel steering angle and rear wheel velocity, this configuration accurately reproduces the steering dynamics of conventional automotive systems, conferring advantages in high-speed stability and trajectory following precision. A schematic diagram of the simplified kinematic model is presented in Fig 4.

The relevant data of the mobile robot in Fig 4 are shown in Equation (15):


$$\begin{array}{c} \left\{ \begin{array}{c} \omega =\frac{\vartheta_{r}-\vartheta_{l}}{\text{2}d}\\ \vartheta =\frac{\vartheta_{r}+\vartheta_{l}}{\text{2}}\\ tan\theta =\frac{L\omega }{\vartheta } \end{array}\right. \end{array}$$ (15)

The parameters in this equation are defined as follows: ω represents the angular velocity of the mobile robot; $\vartheta_{\text{r}}$and $\vartheta_{\text{l}}$ denote the linear velocities of the right and left rear wheels, respectively; d is half the distance between the rear wheels; and ϑ, θ, and l correspond to the robot’s linear velocity, heading angle, and wheelbase length, respectively.

It is assumed that the trajectory of the mobile robot can be decomposed into multiple time slices, and within each time slice ∆t, the mobile robot performs uniform linear motion. The discrete kinematic model of the mobile robot under this condition is shown in Equation (16):


$$\begin{array}{c} \left\{ \begin{array}{c} x_{t+\text{1}}=x_{t}+v\Delta tcos\left(\theta_{t}\right)\\ y_{t+\text{1}}=y_{t}+v\Delta tsin\left(\theta_{t}\right)\\ \theta_{t+\text{1}}=\theta_{t}\;+\;\omega \Delta t\; \end{array}\right. \end{array}$$ (16)

In the equation,（${\text{x}}_{\text{t}},{\text{y}}_{\text{t}}$）denotes the planar coordinates of the mobile robot at time t;${\;\theta }_{\text{t}}$ represents the heading angle of the mobile robot at time t; v(t) and ω(t) denote the linear velocity and angular velocity of the mobile robot, respectively. It should be noted that, during the DWA-based local planning stage, the Ackermann mobile robot is simplified as a kinematically equivalent unicycle model at the vehicle reference point. Under this representation, v denotes the forward velocity and ω denotes the heading-rate of the robot, rather than an independently controllable in-place rotational command. Therefore, infeasible motions such as in-place rotation are excluded during velocity sampling to maintain consistency with the non-holonomic motion characteristics of the Ackermann steering mechanism.

### 4.2 Mobile robot velocity sampling

In the Dynamic Window Approach (DWA), the obstacle avoidance problem for the Ackermann mobile robot is formulated as an optimization problem over a feasible velocity region under the equivalent unicycle representation. This feasible velocity region is subject to three distinct constraints: kinematic physical limits of the robot, dynamic reachability constraints imposed by motor acceleration capabilities, and safety braking constraints that ensure collision-free stopping.

(1) The velocity of the mobile robot is constrained by the mechanical performance, as shown in Equation (17):

$$\begin{array}{c} V_{s}=\left\{(v,,w)|v\in \left[v_{min},v_{max}\right],\omega \epsilon \left[\omega_{min},\omega_{max}\right]\right\} \end{array}$$ (17)

(2) Based on the motor dynamics characteristics of the mobile robot, the reachable velocity within the current cycle Δt is shown in Equation (18):

$$\begin{array}{c} v_{d}\;=\left\{(v,\omega )|v\epsilon \left[v_{c}-{a_{v}}^{min}\Delta t,v_{c}+{a_{v}}^{max}\Delta t\right],\omega \epsilon \left[\omega_{c}-{a_{\omega }}^{min}\Delta t,w_{c}+{a_{\omega }}^{max}\Delta t\right]\right\} \end{array}$$ (18)

In the equation, ${\text{ v}}_{\text{c}}$ and $\omega_{\text{c}}$ denote the linear velocity and angular velocity of the mobile robot at the current moment, respectively; ${{\text{a}}_{\text{v}}}^{\text{min}}$and ${{\text{a}}_{\omega }}^{\text{min}}$represent the minimum linear deceleration and minimum angular deceleration, respectively; ${{\text{a}}_{\text{v}}}^{\text{max}}$ and ${{\text{a}}_{\omega }}^{\text{max}}$denote the maximum linear acceleration and maximum angular acceleration, respectively.
(3) To ensure obstacle avoidance safety, the velocity must meet the braking distance requirements as shown in Equation (19):

$$\begin{array}{c} v_{a}=\left\{v,\omega \right\}|v\leq \sqrt{\text{2}dist(v,\omega ){a_{v}}^{min}}\;,\omega \leq \sqrt{\text{2}dist(v,\omega ){a_{\omega }}^{min}} \end{array}$$ (19)

In the equation, dist(v,ω) is the Euclidean distance between the predicted trajectory and the nearest obstacle.

### 4.3 Evaluation function

The Dynamic Window Approach evaluates predicted trajectories using an evaluation function and outputs the optimal velocity that satisfies the constraints. The basic evaluation function is shown in Equation (20):


$$\begin{array}{c} G(v,\omega )=\sigma \left[\delta \cdot heading(v,\omega )+\eta \cdot vel(v,\omega )+\gamma \cdot dist(v,\omega )\right] \end{array}$$ (20)

In the equation, heading(v,ω) and vel(v,ω) denote the heading angle evaluation function and linear velocity evaluation function of the mobile robot, respectively; dist(v,ω) is the obstacle distance evaluation function; δ, η, and γ are the weight coefficients of each evaluation function; and σ is the evaluation function that indicates normalization of the evaluation function.

## 5 Improved dynamic window approach

A notable limitation of the conventional Dynamic Window Approach lies in its risk assessment mechanism for dynamic obstacles, which predominantly incorporates positional and velocity information while disregarding acceleration—a fundamental dynamic parameter. This study addresses this deficiency through a systematic investigation of the relative acceleration dynamics between the mobile robot and obstacles. Based on this analysis, we propose a dynamic collision risk coefficient model and seamlessly integrate it into the path evaluation function architecture, thereby enhancing the algorithm’s responsiveness to genuinely dynamic threat scenarios.

First, the relative position and relative velocity between the mobile robot and each dynamic obstacle are determined. Let the position of the mobile robot be (${\text{x}}_{\text{r}}$，${\text{y}}_{\text{r}}$) with velocity = ($\vartheta_{\text{r}}$，$\theta_{\text{r}}$), where $\vartheta_{\text{r}}$ is the linear velocity of the robot and $\theta_{\text{r}}$ denotes the direction angle to the dynamic obstacle. The position of the dynamic obstacle is given as(x0，y0) with velocity (v0，$\theta_{\text{0}}$) and acceleration (ax，ay). The relative distance ${\text{d}}_{\text{rel}}$ and relative velocity ${\text{v}}_{\text{rel}}$ between the robot and the obstacle are then calculated using Equations (21) and (22), respectively:


$$\begin{array}{c} d_{rel}=\sqrt{{\left(x_{r}-x_{\text{0}}\right)}^{\text{2}}+{\left(y_{r}-y_{\text{0}}\right)}^{\text{2}}} \end{array}$$ (21)

$$\begin{array}{c} v_{rel}=\sqrt{{\left(v_{r}cos\theta_{r}-v_{\text{0}}-a_{x}t\right)}^{\text{2}}+{\left(v_{r}sin\theta_{r}-v_{\text{0}}-a_{y}t\right)}^{\text{2}}} \end{array}$$ (22)

All collision-time estimates and dynamic collision-risk coefficients used in the simulations were computed based on the corrected relative velocity formulation in Equation (22).

Next, the estimated time to collision ${\text{T}}_{\text{collision}}$ is computed based on the relative distance and relative velocity, provided that the relative velocity is non-zero. In cases where the relative velocity is zero or approximately zero, a large value is assigned to ${\text{T}}_{\text{collision}}$ to prevent division by zero in the calculation. This procedure is formalized in Equations (23) and (24):


$$\begin{array}{c} T_{collision}=\frac{d_{rel}}{v_{rel}} \end{array}$$ (23)

$$\begin{array}{c} D_{dynamic}=\;\frac{\text{1}}{T_{collision}} \end{array}$$ (24)

Then, incorporate the dynamic collision risk coefficient ${\text{D}}_{\text{dynamic}}$ into the evaluation function. The final obstacle avoidance evaluation function is shown in Equation (25):


$$\begin{array}{c} G(v,\omega )=\sigma \left[\delta \cdot heading(v,\omega )+\eta \cdot vel(v,\omega )+\gamma \cdot dist(v,\omega )-\lambda \cdot D_{dynamic}\right] \end{array}$$ (25)

In the equation, λ denotes the weight value of the dynamic collision risk coefficient ${\text{D}}_{\text{dynamic}}$ in the evaluation function.The dynamic collision risk coefficient ${\text{D}}_{\text{dynamic}}$ increases as the robot approaches an obstacle. By subtracting this term, trajectories with higher collision risk receive lower overall scores, thereby being penalized in the selection process.

With the introduction of the dynamic collision risk coefficient, the estimated time to collision exhibits a strong negative correlation with the distance risk term. Specifically, a shorter estimated time to collision indicates a closer proximity between the robot and the dynamic obstacle, which in turn yields a higher distance risk value—signaling the need for immediate obstacle avoidance. Conversely, when the relative distance is large or the relative velocity is low, the distance risk term decreases accordingly, reflecting a controllable collision risk. This relationship is formalized such that the dynamic collision risk coefficient increases significantly under conditions of short relative distance and high relative velocity, thereby providing an intuitive measure of imminent collision threats.

## 6 Fusion algorithm

To achieve effective synergy between global optimality and local real-time performance, this chapter proposes a hierarchical path planning framework that integrates an improved ant colony algorithm with an enhanced dynamic window approach (ACO-DWA-DPP). First, the feasibility of this fusion algorithm is analyzed at both the theoretical and implementation levels. Subsequently, the overall workflow of the algorithm is elaborated in detail: the improved ant colony algorithm plans an initial optimal path within a global static map; guided by this path, the enhanced dynamic window approach performs real-time trajectory planning in local dynamic environments. Meanwhile, a dynamic feedback mechanism is established from the local planning layer to the global planning layer. When the local planner detects that the path is blocked or identifies a superior alternative path, it can trigger online re-optimization of the global path, thereby achieving closed-loop collaboration and iterative optimization between the two layers.

### 6.1 Feasibility analysis

The proposed method is formulated through the synergistic integration of an improved ant colony algorithm and an enhanced dynamic window approach. The feasibility of this integrated framework is systematically evaluated from both theoretical and technical standpoints, with the analysis presented as follows. It is worth emphasizing that the proposed fusion architecture differs fundamentally from previous hybrid planners in its closed-loop design. Existing ACO-DWA fusion methods, such as Xue et al. [22], usually use the ACO-generated global path as guidance for DWA-based local obstacle avoidance, resulting in a mainly one-way planning pipeline. In contrast, the proposed ACO-DWA-DPP framework establishes bidirectional communication: the global path provides guidance for the local DWA planner, while newly detected obstacle information and path blockage conditions from the DWA layer are fed back to the ACO layer for pheromone redistribution and global path re-optimization. This closed-loop mechanism, combined with the acceleration-aware dynamic collision risk coefficient and post-convergence connectivity-based path smoothing, constitutes the primary novelty of this work and distinguishes the proposed framework from existing ACO-DWA fusion approaches.

#### 6.1.1 Theoretical feasibility.

The enhanced ant colony algorithm is developed through strategic improvements in three fundamental areas:

(1) Heuristic function enhancement. A distance-weighted heuristic function is formulated to reinforce the goal-oriented nature of path planning, achieving an optimal balance between exploratory breadth and convergent precision in complex environments.
(2) Adaptive pheromone regulation. Informed by adaptive optimization principles, a dynamic pheromone mechanism is implemented that leverages iteration count and path quality as feedback variables to precisely control pheromone distribution, thereby overcoming the inherent limitations of premature convergence and search stagnation.
(3) Topological path smoothing. A connectivity-based turning point elimination method is introduced, which, from a graph-theoretic perspective, incorporates mobile robot kinematic constraints to ensure both structural rationality and motion feasibility of the resultant path.

The refinement of the dynamic window approach centers on the incorporation of obstacle acceleration parameters. Rooted in Newtonian kinematic principles, this innovation substantially enhances the algorithm’s responsiveness to dynamic environments by minimizing trajectory prediction errors, consequently elevating the safety and reliability of obstacle avoidance maneuvers.

#### 6.1.2 Technical feasibility.

Within the proposed fusion architecture, the improved ant colony algorithm undertakes global path planning at a coarse granularity, whereas the enhanced dynamic window approach is tasked with local trajectory refinement and dynamic obstacle avoidance [22]. These two modules operate in a synergistic manner, with seamless integration facilitated by a jointly defined path cost function.

### 6.2 Path planning process of the fusion algorithm

**Fig 5:**
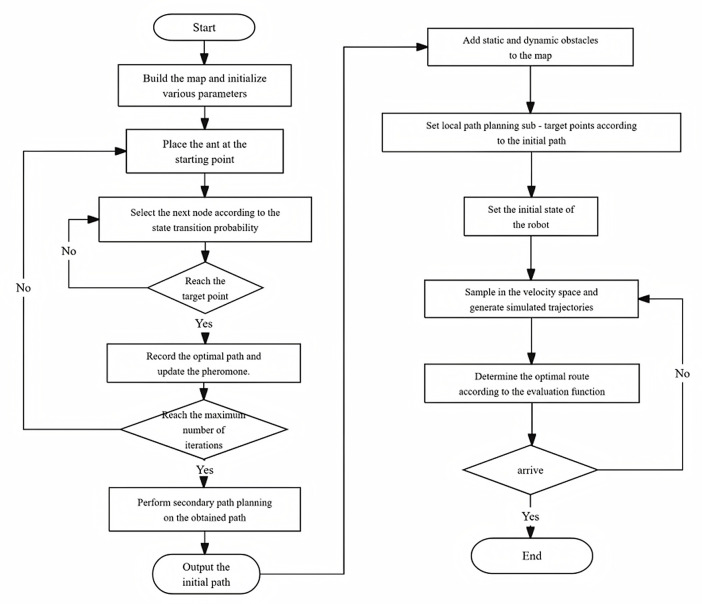
Flowchart of the ACO-DWA-DPP algorithm implementation.

As shown in Fig 5, the implementation process of the fusion algorithm is as follows:

The operational framework of the proposed hybrid algorithm is structured as follows:

(1) Preprocessing: algorithmic enhancement and environmental modeling. The conventional ACO and DWA algorithms are first refined to bolster their efficacy in large-scale complex environments. Simultaneously, a high-fidelity environmental model is constructed to serve as the foundation for all subsequent planning activities.
(2) Global path generation and local module initialization. The enhanced ACO algorithm performs a global search to identify an optimal coarse-grained path. Essential data derived from this process—including nodal coordinates and path quality indicators—are then forwarded to the improved DWA module, thereby initializing its local planning parameters.
(3) Hierarchical collaborative planning. At the macroscopic level, the improved ACO algorithm maintains and optimizes a global reference path within the environmental model. At the microscopic level, the improved DWA algorithm, guided by local sub-targets extracted from this reference path and the robot’s real-time state, samples feasible velocity trajectories and selects the optimal candidate via a predefined evaluation function for execution.
(4) Dynamic feedback and global adaptation. Throughout local navigation, the DWA module continuously assesses path quality. Upon detecting a superior alternative trajectory or encountering an infeasible segment within the global path, it communicates this information back to the ACO module. In response, the ACO algorithm dynamically adjusts the pheromone landscape, enabling real-time refinement of the global path and informed guidance of subsequent search iterations.
It should be noted that the proposed fusion algorithm operates under the assumption that obstacle information—including position, velocity, and acceleration—is continuously available to the mobile robot. In the simulation environment, this is achieved through idealized sensing, where the robot is assumed to have perfect knowledge of obstacles within a predefined detection range at each time step. The term “real-time detection” in this study refers specifically to the algorithmic capability to incorporate newly perceived obstacle data into the local planning cycle and adjust the trajectory accordingly, rather than to the physical sensing process itself. In practical implementations, this would correspond to integrating actual sensor modules such as LiDAR, depth cameras, or ultrasonic sensors, which provide real-time environmental perception. The focus of this work, however, is on the planning and decision-making layer, assuming the perception layer delivers timely and reliable obstacle information.
(5) Iterative optimization and convergence. Steps (3) and (4) are executed in a closed loop, with each iteration progressively enhancing the robot’s trajectory. This synergistic process persists until the mobile robot safely and efficiently reaches its destination within the complex dynamic environment.

## 7 Simulation experiments and analysis of the fusion algorithm

To ensure statistical significance, each algorithm was independently run 30 times in each environment, and all reported performance metrics are presented as mean ± standard deviation. In addition, pairwise Wilcoxon signed-rank tests were conducted between the proposed algorithm and each baseline algorithm, and the corresponding test statistic W and p-value are reported in S2 Table in S1 File. This approach mitigates the influence of randomness and provides a reliable basis for evaluating algorithm performance.

All experiments are conducted in the following environment: the operating system is Windows 11 Home (Chinese edition); the hardware platform consists of an Intel Core i5-9300H processor running at 2.40 GHz; and the algorithm is implemented and simulated using Matlab2019b.

A series of comparative experiments is designed to assess the efficacy of the proposed improved ant colony algorithm (denoted as ACO-DWA-DPP) in path planning tasks. Three reference algorithms are selected for comparison: the traditional ant colony algorithm (ACO), the adaptive ant colony algorithm (IACO), and the multi-strategy fused ant colony algorithm (AACO). All simulations are performed using grid-based environment models at two scales: 20 × 20 and 30 × 30 grids. The 20 × 20 map employs an obstacle coverage rate of 25%, while the 30 × 30 map is tested under two conditions with obstacle coverage rates of 25% and 30%, respectively. The start point is consistently positioned at the upper-left corner (indicated by a green box) and the target point at the lower-right corner (indicated by a red box). The core parameter configurations governing the ant colony algorithms are detailed in Table 1.

**Table 1:** Parameter setting table.

Parameter	Meaning	Value
K	Number of iterations	100
M	Number of ants	50
Alpha	Importance of pheromone	1
Beta	Importance of heuristic factor	4
$\rho_{\text{1}}$	Pheromone evaporation coefficient	0.6
$\rho_{\text{min}}$	Minimum evaporation coefficient	0.1
$\rho_{\text{max}}$	Maximum evaporation coefficient	0.9
Q	Pheromone increment intensity coefficient	10

The core algorithm parameters involved in this paper are set as shown in Table 1. The parameters for the ant colony algorithm (such as the number of iterations K, the number of ants M, the pheromone heuristic factor α, the expected heuristic factor β, and the pheromone volatility coefficient ρ, etc.) are determined with reference to the classic configurations in the literature [7–10] and fine-tuned based on pre-experimental results. The relevant parameters for the Dynamic Window Approach (such as maximum linear velocity, maximum angular velocity, acceleration, etc.) are set according to the actual motion capabilities of the adopted Ackermann mobile robot model, ensuring that the kinematic constraints in the simulation environment are consistent with real-world scenarios.

It should be noted that the parameter values have a certain impact on algorithm performance. The values of the pheromone heuristic factor α and the expected heuristic factor β determine the algorithm’s trade-off between “exploiting historical information” and “exploring new paths”; the volatility coefficient ρ affects the retention rate of pheromones. In the pre-experimental stage, key parameters were preliminarily adjusted using the controlled variable method. The results suggested that the algorithm can maintain relatively stable path planning performance when the parameters vary within moderate ranges, such as α ∈ [0.5, 2], β ∈ [2,6], and ρ ∈ [0.4, 0.8]. However, a more systematic quantitative sensitivity analysis over wider parameter combinations will be conducted in future work to further evaluate the robustness of the proposed method. The parameters selected in this paper are the values that demonstrated the best comprehensive performance within these ranges.

### 7.1 Simulation experiments of the improved ant colony algorithm

(1) Evaluation in simple environmental scenarios.

**Fig 6:**
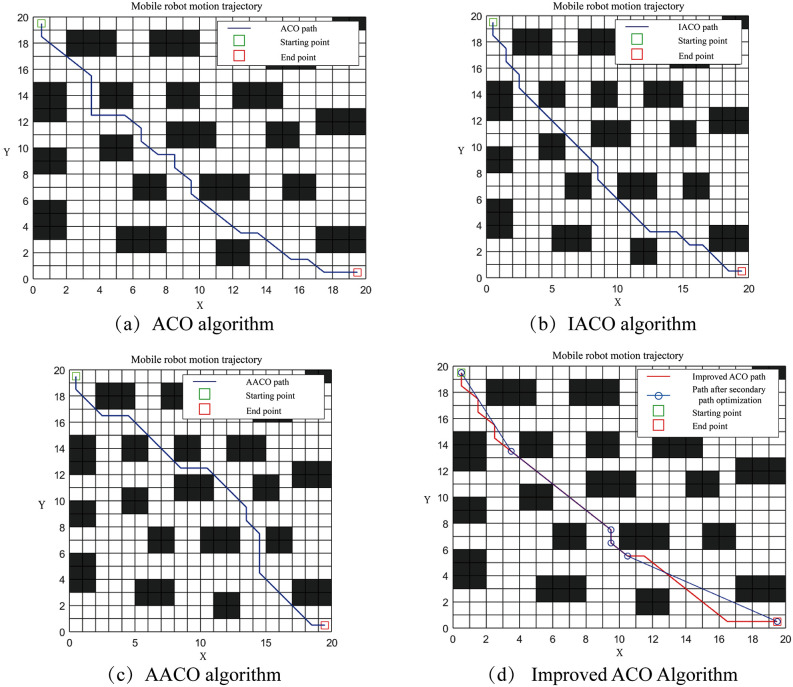
Comparison of path planning in simple environments.

To assess algorithmic performance under uncomplicated conditions, a series of simulations is conducted using a 20 × 20 grid map with 25% obstacle coverage. The resulting paths generated by the four algorithms are illustrated in Fig 6. Comparative analysis yields the following insights. The conventional ACO algorithm yields a path characterized by excessive length, attributable to the inclusion of numerous redundant turning points. The IACO algorithm demonstrates partial improvement over ACO, yet its path retains considerable tortuosity with non-optimal turning point configurations. The AACO algorithm produces a relatively streamlined trajectory; nevertheless, it exhibits a higher frequency of turning points and greater path curvature relative to the proposed method. In marked contrast, the improved ant colony algorithm generates paths with substantially fewer turning points, superior smoothness and linearity, and measurably reduced path length. These findings collectively validate the enhanced efficacy of the proposed algorithm for path planning in simple environmental contexts.

(2) Evaluation in general environmental scenarios

**Fig 7:**
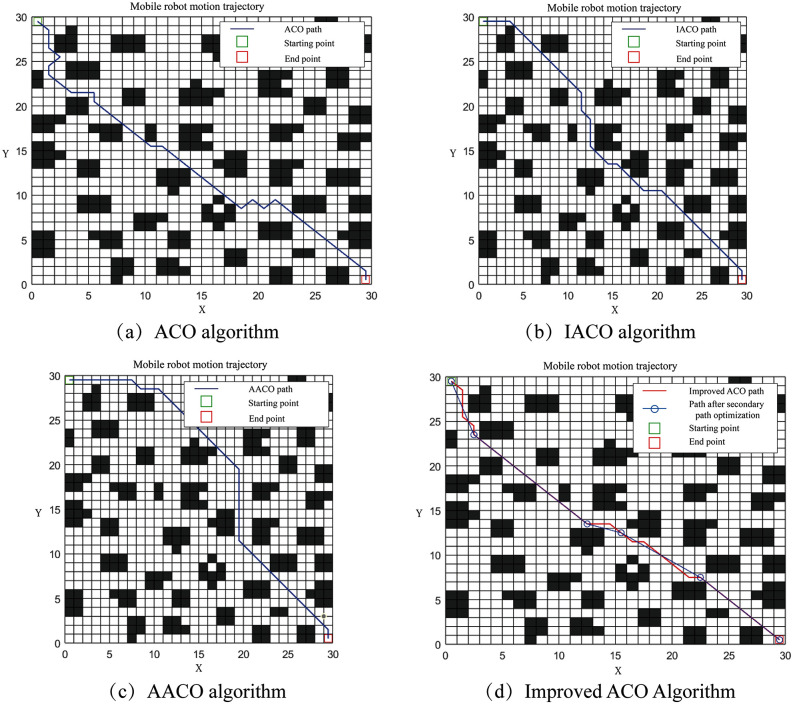
Comparison of path planning in general environments.

A comprehensive evaluation under general conditions is conducted using a 30 × 30 grid map with 25% obstacle coverage. Fig 7 illustrates the paths generated by the four algorithms, revealing distinct performance characteristics. The conventional ACO algorithm produces a trajectory marred by redundant turning points and unnecessary bends, culminating in suboptimal path length and inadequate smoothness. The IACO algorithm demonstrates moderate improvement in path continuity relative to ACO; however, its trajectory in certain segments traverses narrow passages between obstacles, potentially compromising practical feasibility. The AACO algorithm, leveraging its multi-strategy fusion approach, achieves notable reductions in both path length and turning point frequency, yet its global optimality remains subject to further enhancement. In marked contrast, the improved ant colony algorithm introduced in this paper synthesizes the strengths of these methods while incorporating dynamic weight adjustment and probabilistic optimization mechanisms. As quantitatively evidenced in Fig 7, this integrated approach yields a path characterized by minimized length, substantially reduced turning points, and superior geometric smoothness, thereby confirming its enhanced adaptability and robustness for path planning in general environmental contexts.

(3) Evaluation in complex environmental scenarios

**Fig 8:**
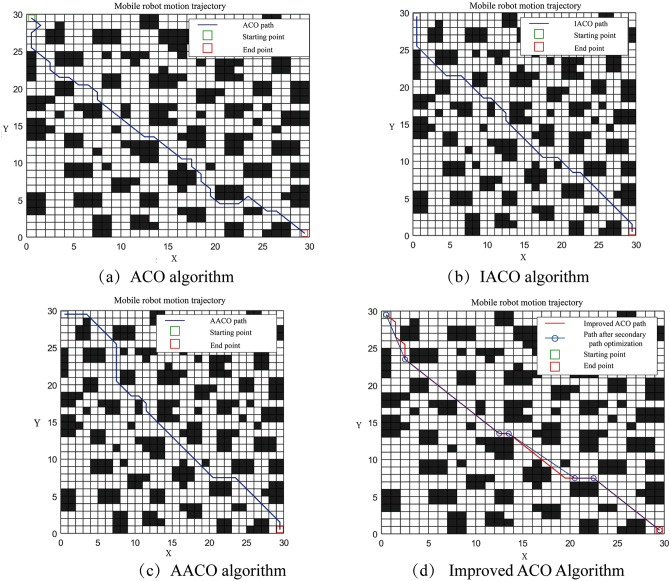
Comparison of path planning in complex environments.

A rigorous evaluation under challenging conditions is conducted using a 30 × 30 grid map with 30% obstacle coverage. Fig 8 presents a comparative visualization of the paths generated by the four algorithms, revealing distinct performance differentials. In environments characterized by dense obstacle distribution, the conventional ACO algorithm produces a trajectory marred by excessive redundant turns and densely clustered turning points, culminating in significantly prolonged path length and severely compromised smoothness. The IACO and AACO algorithms, despite demonstrating localized path refinements, still exhibit frequent turning maneuvers in highly congested regions, yielding trajectories with insufficient global parsimony. In marked contrast, the improved ant colony algorithm proposed herein generates paths characterized by dramatically reduced turning point frequency. Through its synergistic integration of “primary planning” and “secondary optimization” mechanisms, it establishes a highly coherent trajectory that approximates a straight-line connection between origin and destination, thereby achieving substantial reduction in overall path length. These results compellingly demonstrate the proposed algorithm’s superior capabilities in path smoothness, planning efficiency, and trajectory simplicity when operating within complex environmental contexts.

### 7.2 Analysis of simulation experimental results of the improved ant colony algorithm

The raw data supporting the quantitative analysis in Tables 2-5 are available in S1 Data in S1 File.

**Table 2:** Comparison of longest path length results of the four algorithms.

Map type	ACO	IACO	AACO	Improved ACO
Simple environment	187.031 ± 23.426	141.247 ± 16.987	367.293 ± 2.889	109.869 ± 14.114
General environment	294.924 ± 30.845	241.716 ± 24.388	827.920 ± 2.075	147.143 ± 14.163
Complex environment	279.741 ± 20.060	223.091 ± 12.149	771.688 ± 2.600	144.692 ± 20.367

**Table 5:** Comparison of the number of turning points of the four algorithms.

Map type	ACO	IACO	AACO	Improved ACO
Simple environment	12.100 ± 2.454	9.033 ± 2.205	9.533 ± 1.383	4.000 ± 0.000
General environment	21.467 ± 3.060	9.533 ± 2.080	11.067 ± 2.559	4.000 ± 0.000
Complex environment	20.467 ± 3.821	9.300 ± 1.932	10.500 ± 2.389	5.000 ± 0.000

(1) Longest path lengths of the four algorithms in different environments

**Fig 9:**
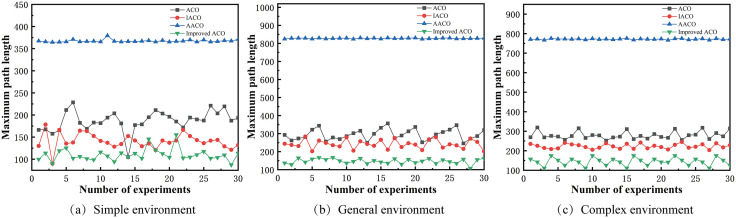
The longest path lengths of four algorithms in different environments.

Fig 9 presents a comparative analysis of the longest path length metric across the four algorithms. The results demonstrate that the improved ACO algorithm achieves the most favorable performance, yielding a shorter path length than the traditional ACO, IACO, and AACO algorithms. This quantitative evidence substantiates the enhanced global optimization capability of the proposed method, confirming its efficacy in path planning tasks. The experimental data illustrated in Fig 9 are further analyzed and synthesized to produce the comparative summary provided in Table 2. It should be noted that the “longest path length” reported in Table 2 refers to the maximum path length observed during the iterative optimization process before convergence. Although AACO occasionally yields longer exploratory paths due to its stochastic nature, the final converged paths of the proposed algorithm consistently outperform all benchmarks. The improvement percentages reported are calculated based on the final converged paths under identical experimental conditions.

The quantitative comparison presented in Table 2 reveals the substantial performance improvements achieved by the proposed algorithm across all tested environmental conditions. In simple, general, and complex scenarios, the longest path length generated by the improved ACO algorithm is reduced by 41.26%, 50.11%, and 48.28%, respectively, relative to the traditional ACO algorithm. When benchmarked against the IACO algorithm, the corresponding reductions are 22.21%, 39.13%, and 35.14%. Most notably, compared with the AACO algorithm, the proposed method yields reductions of 70.09%, 82.23%, and 81.25% across the three scenarios. These empirical findings provide compelling evidence of the enhanced global optimization efficacy and robust path planning performance of the improved algorithm under varying environmental complexities.

(2) Shortest path lengths of the four algorithms in different environments

**Fig 10:**
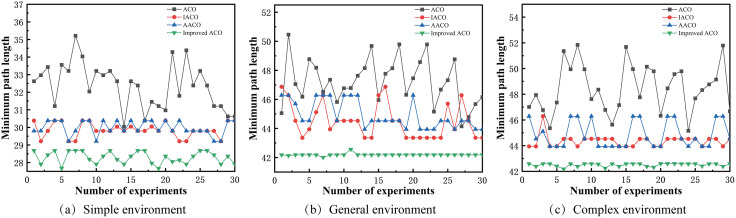
Shortest path lengths of four algorithms in different environments.

As illustrated in Fig 10, the improved ACO algorithm consistently outperforms the ACO, IACO, and AACO algorithms in terms of shortest path length, achieving reductions of varying degrees across different environments. In addition, the proposed method exhibits enhanced stability, evidenced by its reduced fluctuation range in path length and its ability to consistently converge to the same optimal path during the search process. These observations are quantitatively substantiated by the summary statistics provided in Table 3, which are derived from the data presented in Fig 10.

**Table 3:** Comparison of shortest path length results of the four algorithms.

Map type	ACO	IACO	AACO	Improved ACO
Simple environment	32.385 ± 1.299	29.863 ± 0.410	29.994 ± 0.417	28.292 ± 0.345
General environment	47.230 ± 1.598	44.488 ± 1.191	44.937 ± 0.963	42.187 ± 0.078
Complex environment	48.469 ± 1.904	44.351 ± 0.465	44.878 ± 1.050	42.507 ± 0.123

The quantitative comparison presented in Table 3 reveals consistent improvements achieved by the proposed algorithm across all environmental conditions. In simple, general, and complex scenarios, the shortest path length generated by the improved ACO algorithm is reduced by 12.64%, 10.68%, and 12.30%, respectively, relative to the traditional ACO algorithm. When benchmarked against the IACO algorithm, the corresponding reductions are 5.26%, 5.17%, and 4.16%. Compared with the AACO algorithm, the proposed method yields reductions of 5.67%, 6.12%, and 5.28% across the three environments. These empirical findings provide quantitative evidence of the enhanced optimization efficiency and robust path planning performance of the improved algorithm.

(3) Number of iterations of the four algorithms in different environments

**Fig 11:**
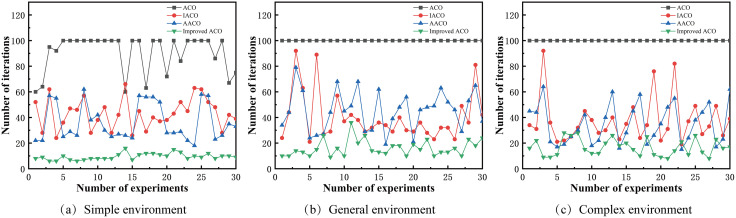
Iteration times of four algorithms in different environments.

As illustrated in Fig 11, the improved ACO algorithm demonstrates a substantial reduction in the number of iterations required for convergence relative to the ACO, IACO, and AACO algorithms. This quantitative evidence confirms its faster convergence rate and enhanced computational efficiency, underscoring the distinct advantages of the proposed method in terms of algorithmic convergence and computational performance. The experimental data presented in Fig 11 are further analyzed and summarized in Table 4.

**Table 4:** Comparison of iteration number results of the four algorithms.

Map type	ACO	IACO	AACO	Improved ACO
Simple environment	90.600 ± 14.680	43.033 ± 11.863	36.267 ± 14.453	9.500 ± 2.515
General environment	100.000 ± 0.000	40.133 ± 18.652	45.367 ± 15.650	16.133 ± 6.329
Complex environment	100.000 ± 0.000	37.600 ± 17.688	35.067 ± 15.671	16.633 ± 6.531

The quantitative comparison presented in Table 4 reveals the substantial convergence improvements achieved by the proposed algorithm across all environmental conditions. In simple, general, and complex scenarios, the iteration count required by the improved ACO algorithm is reduced by 89.51%, 83.87%, and 83.37%, respectively, relative to the traditional ACO algorithm. When benchmarked against the IACO algorithm, the corresponding reductions are 77.92%, 59.80%, and 55.76%. Compared with the AACO algorithm, the proposed method yields reductions of 73.81%, 64.44%, and 52.57% across the three environments. These empirical findings provide compelling quantitative evidence of the enhanced convergence speed and computational efficiency of the improved algorithm, underscoring its superior performance in iterative optimization.

It should also be noted that the number of iterations is not completely equivalent to the total computational cost. Compared with the traditional ACO algorithm, the proposed method introduces additional calculations in the improved heuristic function, pheromone reward-punishment strategy, adaptive evaporation mechanism, and path secondary optimization. Meanwhile, the improved DWA module also adds the calculation of the dynamic collision risk coefficient during local trajectory evaluation. These additional operations increase the computational burden of a single planning cycle to some extent. However, the improved heuristic guidance and pheromone update strategy significantly reduce the number of convergence iterations, as shown in Table 4. In addition, the path secondary optimization is performed only after the ACO algorithm converges, rather than during each iteration. Therefore, the proposed algorithm improves convergence efficiency while maintaining an acceptable computational cost in the tested simulation environments.

(4) Number of turning points of the four algorithms in different environments

**Fig 12:**
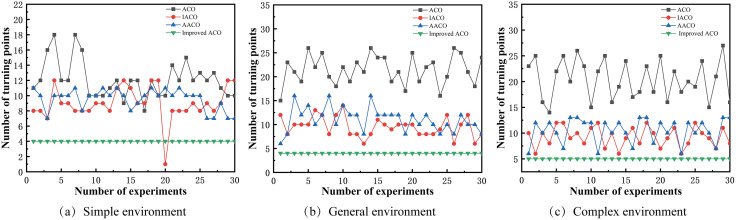
Number of turning points of four algorithms in different environments.

As illustrated in Fig 12, the improved ACO algorithm produces paths characterized by a markedly reduced number of turning points relative to the ACO, IACO, and AACO algorithms. This quantitative evidence confirms the enhanced smoothness and superior path quality achieved by the proposed method, underscoring its distinct advantage in path smoothness optimization. The experimental data presented in Fig 12 are further processed to generate the summary statistics provided in Table 5.

The quantitative comparison presented in Table 5 reveals the substantial smoothness improvements achieved by the proposed algorithm across all environmental conditions. In simple, general, and complex scenarios, the number of turning points generated by the improved ACO algorithm is reduced by 66.94%, 81.37%, and 75.57%, respectively, relative to the traditional ACO algorithm. When benchmarked against the IACO algorithm, the corresponding reductions are 55.72%, 58.04%, and 46.24%. Compared with the AACO algorithm, the proposed method yields reductions of 58.04%, 63.86%, and 52.38% across the three environments. These empirical findings provide compelling quantitative evidence of the enhanced path smoothness achieved by the improved algorithm, underscoring its superior performance in trajectory optimization.

### 7.3 Analysis of obstacle avoidance effect of the improved ant colony algorithm fused with the dynamic window algorithm

In this simulation setup, the mobile robot is assumed to be equipped with an idealized perception system capable of instantly detecting all obstacles within a specified sensing radius at each time step. The obstacles introduced in the simulation—both static and dynamic—are treated as “unknown” to the global planner but become immediately known to the local planner once they enter the robot’s detection range. This setup allows us to evaluate the algorithm’s reactive capability to newly perceived obstacles, which is what we refer to as “real-time detection” in the context of this study.

To assess the dynamic obstacle avoidance capability of the proposed hybrid algorithm, the improved dynamic window approach is configured with the following parameter settings, while the improved ant colony algorithm retains its previously established parameter configuration. The maximum linear velocity and angular velocity of the mobile robot are set to 1.5 m/s and 20.0 rad/s, respectively. The maximum linear acceleration is 0.2 m/s^2^, and the maximum angular acceleration is 50.0 rad/s^2^, with a velocity resolution of 0.02 m/s. The evaluation function is parameterized with coefficients of 0.05 for the azimuth component, 0.2 for the obstacle distance component, and 0.3 for the current velocity component. The prediction time window is set to 3 s.

**Fig 13:**
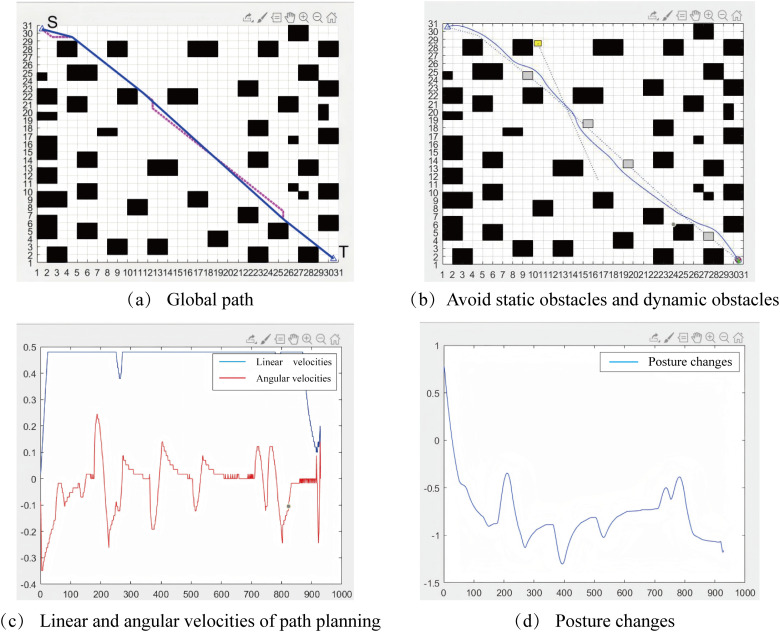
Path planning of the fusion algorithm.

A simulation environment is constructed using a 30 × 30 grid map with a static obstacle coverage rate of 20% to assess the dynamic obstacle avoidance capability of the proposed hybrid algorithm. Building upon the global path generated by the improved ant colony algorithm, multiple unforeseen static obstacles and one dynamic obstacle are introduced to challenge the algorithm’s real-time responsiveness. It should be noted that this scenario mainly evaluates the algorithm’s response to a single moving obstacle combined with multiple unknown static obstacles. Therefore, the present experiment verifies the basic dynamic obstacle avoidance capability of the proposed method, while more complex scenarios involving multiple simultaneously moving obstacles remain to be further investigated. The obstacle avoidance performance of the integrated approach—combining the improved ant colony algorithm with the dynamic window method—is systematically illustrated in Fig 13. Specifically, subfigure (a) presents the global trajectory from the start point “S” to the target point “T” under obstacle-free conditions; (b) depicts the motion scenario following the introduction of unknown static and dynamic obstacles; (c) records the temporal evolution of linear velocity (blue curve) and angular velocity (red curve) throughout the planning process; and (d) shows the corresponding variation in the mobile robot’s attitude angle.

The performance of the proposed fusion algorithm is systematically evaluated across four dimensions in Fig 13. Figure (a) depicts the global trajectory generated by the improved ant colony algorithm under static conditions, establishing a reference path for subsequent local planning. Figure (b) demonstrates the algorithm’s adaptive capability in response to unforeseen static and dynamic obstacles. The trajectory exhibits smooth detours while maintaining progress toward the target, underscoring the robustness of the integrated framework in handling environmental unpredictability. Figure (c) provides quantitative insight into motion control through the temporal evolution of linear and angular velocities. The stability of linear velocity combined with agile angular adjustments reflects the dynamic window planner’s fidelity in tracking global path instructions and its rapid reactivity to emergent obstacles. Figure (d) illustrates the attitude angle variation, which correlates strongly with path curvature and obstacle avoidance maneuvers, confirming the algorithm’s capacity to preserve motion continuity and controllability during intricate navigation tasks. Collectively, these results substantiate that the proposed fusion algorithm enables safe, reliable, and efficient autonomous navigation in dynamic unknown environments.

The experimental results in Fig 13 also validate the three novel aspects of our fusion framework. First, the successful avoidance of unknown obstacles demonstrates the effectiveness of the bidirectional feedback mechanism—the local planner detected environmental changes and implicitly guided the robot around obstacles, even though the global path was initially planned without knowledge of these obstacles. Second, the smooth velocity adjustments in Fig 13(c), particularly the controlled deceleration when approaching dynamic obstacles, reflect the benefit of the acceleration-aware risk coefficient, which enables more anticipatory rather than reactive avoidance. Third, the final trajectory’s smoothness, despite the presence of multiple obstacles, confirms that the post-convergence smoothing does not compromise the path’s feasibility or optimality.

Although the parameters selected in this paper can achieve favorable path planning results, variations in parameter values do indeed have a certain impact on algorithm performance. Generally speaking, if the pheromone heuristic factor α is too large, the algorithm can easily fall prematurely into local optima; if the expected heuristic factor β is too large, ants may overly rely on distance information and neglect the guiding role of pheromones; if the volatility coefficient ρ is too large, pheromones dissipate quickly, leading to slow algorithm convergence, whereas if ρ is too small, pheromone accumulation may be too slow, making it difficult to form effective positive feedback. The evaluation function weights in the Dynamic Window Approach (δ, η, γ, λ) also influence obstacle avoidance behavior: if the obstacle distance weight γ is too large, the robot may become overly conservative, resulting in excessively long detour paths; if the dynamic risk coefficient weight λ is too small, the response to dynamic obstacles may lag.

In the parameter selection process, this paper comprehensively considered multiple factors such as algorithm convergence speed, path quality, and obstacle avoidance safety, and validated the choices through pre-experiments. In subsequent research, adaptive parameter adjustment strategies or metaheuristic optimization algorithms could be further adopted to dynamically optimize the parameters, thereby enhancing the algorithm’s adaptability in diverse environments.

## 8 Conclusion

This paper has presented a collaborative path planning framework that integrates an improved ant colony algorithm with an enhanced dynamic window approach (ACO-DWA-DPP) for mobile robot navigation in dynamic environments. The core contribution lies not merely in the individual algorithmic enhancements, but in their synergistic integration through a bidirectional closed-loop architecture that addresses fundamental limitations of prior hybrid planners.

The experimental results yield several insights of practical significance for mobile robot path planning:

First, the bidirectional feedback mechanism demonstrates that global and local planning need not be treated as sequential modules. By enabling local perception to trigger global path re-optimization, the framework achieves a balance between computational efficiency and environmental adaptability that is difficult to attain with unidirectional approaches. This finding suggests that future hybrid planners should prioritize closed-loop architectures over traditional pipeline designs.

Second, the incorporation of acceleration into the dynamic collision risk model proves particularly valuable in scenarios involving unpredictable obstacle motion. The improved responsiveness observed in simulations indicates that acceleration-aware risk assessment can reduce collision risks without excessive conservatism—a trade-off that conventional velocity-based methods struggle to achieve. This has implications for real-world deployment where obstacle acceleration is often non-negligible.

Third, the post-convergence smoothing strategy offers a practical lesson for algorithm design: path quality optimization and iterative search can be decoupled without sacrificing performance. By applying topological smoothing only after convergence, the method preserves the efficiency of pheromone-guided exploration while still producing kinematically feasible trajectories. This decoupling principle may benefit other swarm intelligence algorithms facing similar trade-offs between search efficiency and solution quality.

Another limitation of the current validation is that the dynamic obstacle avoidance experiment only considers one moving obstacle together with multiple static obstacles. Although this setting verifies the basic real-time response capability of the proposed acceleration-aware DWA module, it is not sufficient to fully characterize the algorithm’s performance in highly dynamic environments with multiple simultaneously moving obstacles. Therefore, future work will prioritize the construction of multi-obstacle dynamic scenarios with different velocities, accelerations, and motion directions to further evaluate the robustness of the proposed closed-loop planning framework. It should be noted that the proposed algorithm has currently only been validated in a simulation environment. Although the simulation environment has been designed to be as close to real-world scenarios as possible, physical experiments remain the ultimate standard for verifying the practicality of an algorithm. In future work, we plan to deploy the proposed algorithm on a mobile robot platform equipped with an Ackermann steering mechanism to further test its path planning performance and dynamic obstacle avoidance capabilities in complex real-world environments. Based on the results of these physical experiments, we will optimize and adjust the algorithm parameters and model accordingly.

## 9 Future work

Although the fusion algorithm proposed in this paper has achieved satisfactory path planning results in simulation environments, several research directions remain worthy of further exploration. Future work will be pursued from the following aspects:

(1) Dynamic Adaptive Optimization of Algorithm Parameters
The key parameters in this paper (such as the pheromone heuristic factor α, the expected heuristic factor β, the volatility coefficient ρ, and the evaluation function weights γ, λ, etc.) currently adopt fixed values. Although they have been calibrated through pre-experiments, they may not always remain optimal when the environment changes dynamically. In the future, reinforcement learning or metaheuristic optimization algorithms (such as Particle Swarm Optimization and Differential Evolution) could be introduced to enable adaptive online adjustment of parameters, allowing the algorithm to dynamically balance exploration and exploitation capabilities according to environmental complexity, thereby further enhancing its robustness in diverse scenarios. In addition, future work will include a more systematic quantitative sensitivity analysis of key parameters, including α, β, ρ, and the DWA evaluation weights, to provide clearer guidance for parameter selection in different environments.
(2) Multi-Robot Collaborative Path Planning in Complex Dynamic Environments
This study focuses on the path planning problem for a single robot. However, real-world scenarios such as warehouse logistics and smart factories often involve the collaborative operation of multiple robots [23]. Future work could extend the proposed algorithm to multi-robot systems, investigating path conflict resolution mechanisms between robots, dynamic priority allocation strategies, and communication topology optimization, thereby achieving efficient collaboration and collision avoidance among multiple robots within confined spaces.
(3) Validation of Algorithm Generalization Ability in More Complex Environmental Scenarios
Although this paper has set up three types of environments—simple, general, and complex—real-world application scenarios are often more challenging, featuring situations such as narrow passages, U-shaped traps, and unstructured obstacle distributions. In future work, a greater variety of benchmark environments (e.g., maze maps, randomly generated maps, and scenarios with dense dynamic obstacles) could be designed to systematically evaluate the algorithm’s path search capability and obstacle avoidance performance under different topological structures. Furthermore, the possibility of integrating the algorithm with deep reinforcement learning could be explored to enhance its adaptability to unknown environments. In addition, the current simulations are mainly conducted on 20 × 20 and 30 × 30 grid maps, which are relatively small compared with large-scale warehouse or outdoor navigation scenarios. As the map scale increases, the computational cost of ant colony search and the interaction between global and local planning layers may also increase. Therefore, future work will further evaluate the scalability of the proposed framework on larger grid maps and more complex environments, and explore efficiency-improvement strategies such as hierarchical planning, map decomposition, and adaptive search-space reduction.
(4) Real-Time Path Planning Integrated with Perception Systems
Current path planning research often assumes that environmental information is completely known or that obstacle positions can be acquired in real time, without fully considering practical factors such as sensor noise, perception latency, and mapping errors. Future work could deeply integrate the proposed algorithm with a Simultaneous Localization and Mapping (SLAM) system, investigating uncertainty modeling in partially observable environments to enhance the algorithm’s robustness and real-time performance under realistic perception conditions.
(5) Physical Platform Validation and Engineering Application
This study has currently only been validated in a simulation environment, whereas physical experiments represent the ultimate standard for testing algorithm practicality. Future plans include deploying the proposed algorithm on a mobile robot platform equipped with an Ackermann steering mechanism to test its path planning performance and dynamic obstacle avoidance capabilities in complex real-world environments. Simultaneously, we will optimize and adjust the algorithm model and parameters to address issues such as dynamic response delays and control accuracy errors that arise during physical experiments, thereby advancing it toward engineering application.

## Supporting information

S1 File S1 Data. Raw data from the simulation experiments reported in the manuscript. **S2 Table.** Wilcoxon signed-rank test results for pairwise comparisons between the proposed algorithm and baseline algorithms.
(ZIP)
