# Supplementary material for: Path planning for mobile robots by fusing ant colony optimization and dynamic window approach
Source: PLoS One. 2026 Jul 27;21(7):e0340336. doi: 10.1371/journal.pone.0340336 (PMC13405125; doi:10.1371/journal.pone.0340336)
Supplement: Attachment — Submitted filename: Response to Reviewers.docx [file pone.0340336.s003.docx]

Dear Editor and Reviewers:

Thank you very much for taking the time to review our manuscript (Manuscript ID: PONE-D-25-67110) and for providing valuable and constructive comments. These insightful suggestions are of great significance in guiding us to improve the quality of our paper. We have carefully read and thoroughly discussed all the comments and have made comprehensive revisions to the manuscript accordingly. Below, we respond point-by-point to the comments raised by the Editor, Reviewer 1, Reviewer 2, and Reviewer 3, in that order, and provide a detailed explanation of the modifications made. All changes have been highlighted in red in the revised manuscript for your ease of review. Once again, we sincerely thank you for your hard work and thoughtful guidance.

**Response to Editor**

1：Please ensure that your manuscript meets PLOS ONE's style requirements, including those for file naming.

Response：Dear Editor, thank you for reviewing and providing guidance on our manuscript. In response to your comment—"Please ensure that your manuscript meets PLOS ONE's style requirements, including those for file naming"—we have conducted a comprehensive review and made necessary adjustments to the manuscript in accordance with the official PLOS ONE submission guidelines.

1、Manuscript Formatting: We have confirmed that the manuscript uses double-spacing and includes consecutive page numbers and line numbers throughout.

2、Figure and Table Formatting: All figures have been removed from the main manuscript file and are now uploaded separately as high-resolution TIFF files (resolution ≥300 dpi, meeting journal requirements).

3、File Naming: Following journal specifications, the main manuscript file has been renamed "Manuscript.docx," and the figure files have been renamed "Fig1.tif," "Fig2.tif," etc., ensuring consistency with the citations within the text.

4、Reference Formatting: The references have been uniformly formatted in the Vancouver style, ensuring compliance with ICMJE guidelines。

5、Manuscript Structure: We have verified that the manuscript contains all essential sections, including Title Page, Abstract, Introduction, Methods, Results, Discussion, Acknowledgments, and References, arranged in the order required by the journal.

All the aforementioned modifications have been highlighted in the revised manuscript. We believe that after these adjustments, the manuscript now fully complies with the submission guidelines of PLOS ONE. Should there be any further adjustments required, please do not hesitate to inform us, and we will cooperate fully.

Thank you once again for your time and valuable comments.

2：Please note that PLOS One has specific guidelines on code sharing for submissions in which author-generated code underpins the findings in the manuscript.

Response：Thank you for reviewing our manuscript and providing guidance, and for drawing our attention to PLOS ONE's important policy on code sharing. We have carefully read the guidelines you provided ([https://journals.plos.org/plosone/s/materials-and-software-sharing#](https://journals.plos.org/plosone/s/materials-and-software-sharing)) and fully understand and support the journal's requirement that "all code generated by authors must be made available without restriction upon publication of the work." We recognize that this requirement is crucial for promoting the reproducibility and transparency of scientific research.

We are currently in the process of finalizing the code, improving annotations, and preparing documentation to ensure it meets best practice standards—namely, that the code is clear, well-documented, and includes necessary dependency descriptions and test data to facilitate replication and reuse by other researchers. We plan to upload the complete and properly formatted code to a public code repository that meets PLOS ONE's requirements and can issue a persistent identifier (such as a DOI), such as Zenodo or Figshare, after the paper is formally accepted but before publication. An appropriate open-source license (such as MIT or BSD) will also be adopted.

We believe that this arrangement will both fully comply with the journal's policy requirements and ensure the quality of the code ultimately shared. Thank you for your understanding and valuable comments.

3：Please note that funding information should not appear in any section or other areas of your manuscript. We will only publish funding information present in the Funding Statement section of the online submission form. Please remove any funding-related text from the manuscript.

Response：Thank you for reviewing our manuscript and providing guidance, and for pointing out the formatting requirements regarding funding information. We have carefully read your comments and fully understand PLOS ONE's policy: funding information should only be included in the Funding Statement section of the online submission form and must not appear in any part of the manuscript or other areas.

Accordingly, we have removed all funding-related text from the manuscript. The revised manuscript has been ensured to contain no funding information whatsoever.

We confirm that this modification complies with the journal's formatting requirements. Should there be any further adjustments required, please do not hesitate to inform us, and we will cooperate fully.

Thank you once again for your time and valuable comments.

4：Thank you for stating the following financial disclosure:“This work was supported by the Key Research and Development Project of the Shanxi Provincial Department of Science and Technology (202102140601015), the 2023 Scientific and Technological Innovation Project of Jinzhong National Agricultural High-tech Zone (Taigu National Science and Technology Innovation Center) (JZNGQBSGZZ004), and the Shanxi Provincial Basic Research Program (202403021222112).”Please state what role the funders took in the study. If the funders had no role, please state: "The funders had no role in study design, data collection and analysis, decision to publish, or preparation of the manuscript."If this statement is not correct you must amend it as needed.Please include this amended Role of Funder statement in your cover letter; we will change the online submission form on your behalf.

Response：Thank you for reviewing our manuscript and providing guidance. In response to your comments regarding funding information and the funders' role statement, we have revised the manuscript and cover letter as requested, as detailed below:

1、All funding-related text has been removed from the manuscript. The revised manuscript contains no funding information whatsoever, with this information now only included in the appropriate sections of the online submission system.

2、At the end of the cover letter, prior to the signature, we have added a statement listing all funding sources along with the required role statement. The specific addition reads as follows:This work was supported by the following funding projects: the Key Research and Development Project of the Shanxi Provincial Department of Science and Technology (202102140601015), the 2023 Scientific and Technological Innovation Project of Jinzhong National Agricultural High-tech Zone (Taigu National Science and Technology Innovation Center) (JZNGQBSGZZ004), and the Shanxi Provincial Basic Research Program (202403021222112). The funders had no role in study design, data collection and analysis, decision to publish, or preparation of the manuscript.

The revised manuscript and cover letter are attached to this email for your reference. Should there be any further adjustments required, please do not hesitate to inform us, and we will cooperate fully.

Thank you once again for your time and valuable comments.

5：Please ensure that you refer to Figure 5 in your text as, if accepted, production will need this reference to link the reader to the figure.

Response：Thank you for reviewing our manuscript and providing guidance. In response to your comment, we have revised the manuscript accordingly. The specific modifications are as follows: At the beginning of Section 6.2 "Path Planning Process of the Fusion Algorithm" (line 465), we have added a clear citation statement: "As shown in Fig. 5, the implementation process of the fusion algorithm is as follows:" This ensures that readers are aware of the existence of Figure 5 and its relevance to the text before reading the content. Additionally, we have reviewed all figures throughout the manuscript to ensure that each figure (Figure 1 through Figure 13) is appropriately cited in the main text.

The revised manuscript is attached to this email for your review. Should there be any further adjustments required, please do not hesitate to inform us, and we will cooperate fully. Thank you once again for your time and valuable comments.

6：Please include captions for your Supporting Information files at the end of your manuscript, and update any in-text citations to match accordingly. Please see our Supporting Information guidelines for more information:<http://journals.plos.org/plosone/s/supporting-information>.

Response：Thank you for reviewing our manuscript and providing guidance. In response to your comment, we have revised the Supporting Information files and the manuscript as requested, with the following specific modifications:

1.Supporting Information File Processing: The folder "1_Fig.tif" has been renamed to "S1_Fig," which contains all the figures from the manuscript. The data file "2_Data.xlsx" has been renamed to "S1_Data.xlsx," which contains the raw data from all simulation experiments.

2. Supporting Information Section Added:At the end of the manuscript (lines 858-860), we have added the Supporting Information title and descriptions:

14、Supporting Information

S1_Fig: High-resolution TIFF versions of all figures from the manuscript.

S1_Data.xlsx: Raw data from the simulation experiments reported in the manuscript.

3、In-Text Citation Added: In Section 7.2 (line 613), we have added the following citation: "The raw data supporting the quantitative analysis in Tables 2-5 are available in S1 Data."

The revised manuscript and Supporting Information files are attached to this email for your review. Should there be any further adjustments required, please do not hesitate to inform us, and we will cooperate fully. Thank you once again for your time and valuable comments.

7：Please remove your figures from within your manuscript file, leaving only the individual TIFF/EPS image files, uploaded separately. These will be automatically included in the reviewers’ PDF.

Response：Thank you for reviewing our manuscript and providing guidance. In response to your comment, we have revised the manuscript and figure files as requested, with the following specific modifications:

Manuscript File Modifications: All embedded images (Figure 1 through Figure 13) have been removed from the manuscript, with only the corresponding figure captions retained.

Preparation of Individual Figure Files:All 13 figures have been exported as TIFF format files, each meeting the journal's resolution requirements (≥300 dpi). The figure files have been named "Fig1.tif" through "Fig13.tif" and are uploaded as separate files to the submission system.

We confirm that all figures are properly cited in the main text (as Figures 1-13), and the revised manuscript format complies with PLOS ONE's submission requirements. Should there be any further adjustments required, please do not hesitate to inform us, and we will cooperate fully. Thank you once again for your time and valuable comments.

8：Thank you for providing your underlying data as Supporting Information.We note that the data set contains text or data that is not in English. Please note that PLOS is an English-language publisher, so we require data sets to be provided in English as well. Please upload an English-language version of your data set.This will also allow us to determine if your data follows PLOS standards per our Data Availability policy here: https://journals.plos.org/plosone/s/data-availability

Response：Thank you for reviewing our manuscript and providing guidance, and for drawing our attention to the language requirements for datasets. We have carefully read the PLOS Data Availability Policy you provided and fully understand that PLOS, as an English-language publisher, requires all supporting information data to also be provided in English.

In response to your comment, we have conducted a comprehensive review and revision of the underlying dataset: All non-English text within the file (including column headers, worksheet names, etc.) has been converted entirely to English. The revised dataset is now presented completely in English, ensuring compliance with PLOS's Data Availability Policy and facilitating understanding, validation, and reuse by colleagues in the international academic community.

The updated English-version dataset file (S1_Data.xlsx) has been re-uploaded to the submission system.

We confirm that the revised dataset complies with PLOS's best practices for data sharing. Should there be any further adjustments required, please do not hesitate to inform us, and we will cooperate fully.

Thank you once again for your time and valuable comments.

9：If the reviewer comments include a recommendation to cite specific previously published works, please review and evaluate these publications to determine whether they are relevant and should be cited. There is no requirement to cite these works unless the editor has indicated otherwise.

Response：

Thank you for reviewing our manuscript and providing guidance. We fully understand and have complied with your comment regarding: "If the reviewers' comments include suggestions to cite specific published literature, please review and evaluate whether these references are relevant and should be cited."

During this revision process, we have carefully examined all the literature suggested for citation by the reviewers and have conducted our evaluation based on the following principles:

Relevance:Assessing whether the literature is directly related to the methodology, results, or discussion of this study;

Necessity: Determining whether citing the literature would enhance the academic integrity of the paper or provide important background for readers;

Avoidance of Redundant Citations: Ensuring that non-essential references are not added merely because they were "suggested for citation."

We confirm that the final version of the reference list has followed the editor's instructions and has not added any unnecessary references due to "suggested citations." Should there be any further adjustments required, please do not hesitate to inform us.

Thank you once again for your time and valuable comments.

10：Please review your reference list to ensure that it is complete and correct. If you have cited papers that have been retracted, please include the rationale for doing so in the manuscript text, or remove these references and replace them with relevant current references. Any changes to the reference list should be mentioned in the rebuttal letter that accompanies your revised manuscript. If you need to cite a retracted article, indicate the article’s retracted status in the References list and also include a citation and full reference for the retraction notice.

Response：

Thank you for reviewing our manuscript and providing guidance. In response to your comment, we have conducted a comprehensive and meticulous verification of the entire reference list in the manuscript. The specific tasks completed are as follows:

Completeness Verification:We have checked one by one all citations in the main text (23 in total) against the reference list at the end of the manuscript (numbered [1] to [23]), ensuring a one-to-one correspondence with no omissions or redundant entries.

Accuracy Check:We have verified the author names, titles, journal names, years, volume/issue numbers, and page ranges for each reference. Individual formatting inconsistencies (such as journal name abbreviation standards) have been corrected to ensure that all entries comply with the journal's formatting requirements.

Retraction Status Verification:We have searched all references in the list through the CrossRef and Retraction Watch databases. Upon verification, none of the cited papers have been retracted; all are valid and publicly accessible literature. Therefore, no retraction reasons need to be stated in the text, nor are replacements required.

The revised reference list has been appended at the end of the revised manuscript. We confirm that it is complete, accurate, and compliant with journal standards. Should there be any further adjustments required, please do not hesitate to inform us.

Thank you once again for your time and valuable comments.

## **Response to Reviewer 1**

1：The abstract claims the algorithm reduces the longest path length by 41.26%~48.28% compared to traditional ACO, but Table 2 shows AACO actually produces much longer paths than ACO in some cases, which undermines the validity of this comparison baseline.

Response：We thank the reviewer for this insightful observation. We agree that the comparison benchmark must be consistent and meaningful. The longer paths produced by AACO are due to its stochastic exploration mechanism in complex environments, which occasionally generates suboptimal trajectories during early iterations. However, the longest path metric reported in Table 2 refers to the maximum path length recorded throughout the iterative process, rather than the final converged path. To avoid confusion and ensure a fair comparison, we have revised the relevant text to clarify that the improvement percentages are calculated based on the final converged paths of each algorithm. Additionally, we have adjusted the wording in the Abstract (line 19) and Section 7.2 (lines 621-626) to explicitly state that the comparisons were conducted under identical experimental conditions and that the reported improvements reflect the superior convergence capability of the proposed algorithm.

2：Equation (8) defines attractive force, which contradicts standard potential field theory where attraction typically increases as distance decreases; Pls check all the equations and definitions.

Response：

We thank the reviewer for this valuable comment regarding our equation (8):$F=\mu\cdot{d_{\mathrm{iE}}}^{2}$ The reviewer points out that the attractive force defined by us does not align with the classical artificial potential field method, where the attractive force typically increases as the distance decreases. We fully understand this observation and provide our explanation below.

In the classical potential field method, the attractive force is usually designed as a function proportional to the distance, enabling the robot to gradually approach the target. However, in our improved ant colony algorithm, the "attractive force" defined in equation (8) does not act independently as a physical force on robot motion; rather, it serves as a distance weighting factor embedded within the heuristic function, functioning to adjust the probability of node selection during the path search process. Specifically, this factor combines with the subsequent exponential decay term 0.9F to form part of the heuristic function η(i). When a node is far from the target, F is large, causing 0.9F to decrease rapidly, thereby reducing the heuristic value of that node and discouraging ants from prematurely exploring regions distant from the target. Conversely, when a node is close to the target, F is small, resulting in less decay of 0.9F and a relatively higher heuristic value, thus guiding ants to focus on areas near the target. This design aims to balance the algorithm's global exploration capability with local convergence speed, avoiding blind search in early stages, and is not intended to simulate a real potential field force.

Furthermore, the heuristic function also incorporates a directional deviation term and a dynamic risk assessment term, which work together with the distance weighting factor to form a comprehensive evaluation of node quality. As the reviewer insightfully noted, when the robot approaches obstacles, the risk assessment term implicitly creates a "repulsive" effect, steering the algorithm away from hazardous areas. Meanwhile, guidance toward the target is maintained through the distance weighting factor and directional deviation term, ultimately directing the ant colony's search behavior progressively toward the goal. Therefore, although equation (8) in isolation may appear inconsistent with the classical potential field definition, it effectively promotes goal orientation while ensuring safe obstacle avoidance through the synergistic action of the overall heuristic function.

We fully respect your opinion and understand the potential ambiguity in the formulation of this equation. If the reviewer believes that the above explanation still does not fully address the issue, or if there are errors requiring further correction, we are very willing to make adjustments and improvements in subsequent revisions based on your suggestions. Once again, we thank the reviewer for the meticulous review; your comments are of great significance in enhancing the rigor and readability of this paper.

3：The paper states that redundant turning points are removed via connectivity checks, but it doesn't clarify whether this post-processing step is applied after every ACO iteration or only once at the end, affecting computational cost claims.

Response：

We sincerely thank the reviewer for their attention to the execution timing of the secondary optimization step. The description of this process in the original manuscript was indeed somewhat unclear in its expression. It should be clarified that the path smoothing procedure based on connectivity checking is executed only once, after the improved ant colony algorithm has converged to the final global path. This design is intended to ensure that the computational cost of the smoothing process does not interfere with the pheromone update mechanism during the iterative process, thereby maintaining the efficiency of the global search. We have revised the relevant description in Section 3.4 (lines 317-321) of the revised manuscript to clarify this point and have made corresponding adjustments to the discussion of computational cost.

4：The evaluation function in Equation (25) adds D_dynamic as a positive term, yet higher collision risk should penalize trajectories—this suggests a possible sign error or misinterpretation of the risk coefficient's role.

Response：

We thank the reviewer for pointing out this potential inconsistency in the formulation. Upon re-examination, we agree that the original expression may have been misleading. In our implementation, D_dynamic is defined as 1/Tcollision, which increases as the estimated time to collision decreases. Therefore, a higher D_dynamic indeed indicates a higher collision risk. To ensure that high-risk trajectories are properly penalized, this term should either be subtracted in the evaluation function or assigned a negative weight. We have chosen to revise the equation to reflect the subtraction, which is more intuitive, and the text has been updated accordingly (lines 432–436).

5：The claim that the fusion algorithm detects unknown obstacles in real time isn't substantiated by any sensor model or perception module description, making the “real-time detection” assertion appear unsupported.

Response：

We sincerely acknowledge this valid criticism. In the current manuscript, we focused primarily on the algorithmic fusion aspect without providing a detailed explanation of the perception layer. To address this issue, we have added a clarification in Section 6.2 (lines 512-522) and Section 7.3 (lines 694-699) stating that the simulations assume idealized perception capabilities (e.g., perfect obstacle detection within a certain range), and that real-time detection refers to the algorithmic response time to perceived obstacle information, rather than the physical sensing process. We also suggest future work to integrate actual sensor models (such as LiDAR, depth cameras) for real-world validation.

6：For path planning applications, the author could consider more articles: 3D vision technologies for a self-developed structural external crack damage recognition robot; Automation in Construction.

Response：

We greatly appreciate the reviewer for taking the time to review our manuscript and for recommending the insightful study on "3D vision technology for a self-developed structural external crack damage recognition robot" published in the journal Construction Automation. The literature recommended by the reviewer indeed provides us with a new perspective, particularly in the areas of robot perception and environmental modeling. The research approach in 3D vision technology holds significant reference value for our future work.

We have carefully studied the main content of this recommended paper and found that it focuses on the specific application scenario of structural crack damage identification, which differs to some extent from the path planning problem for mobile robots in complex dynamic environments explored in this paper, both in terms of research objectives and core methodologies. The primary focus of our paper is to enhance global path optimization efficiency and local dynamic obstacle avoidance safety through the fusion of an improved ant colony algorithm and the dynamic window approach. Therefore, within the current framework of our paper, it may be challenging to fully demonstrate the academic value of the 3D vision technology addressed in that literature.

Nevertheless, we are sincerely grateful for the reviewer's suggestion, as it has opened up interdisciplinary research horizons for us. In our future research work, we will actively follow and draw upon cutting-edge achievements in the field of robot perception and vision technology, with the aim of further refining and expanding our research framework. Once again, we sincerely thank the reviewer for their thoughtful guidance and valuable suggestions!

## **Response to Reviewer 2**

1：Clearly articulate the novelty of their approach relative to prior hybrid planners.

Response：

We thank the reviewer for this valuable suggestion. We have made substantial revisions to the Introduction (lines 86-101) and Section 6 (lines 461-467) to more clearly articulate the novel contributions of our work relative to existing hybrid planners. Specifically, we now explicitly contrast our approach with prior ACO-DWA fusion methods by highlighting three key innovations: (1) the bidirectional closed-loop feedback mechanism between the global and local planners, which goes beyond traditional unidirectional guidance; (2) the integration of a dynamic collision risk coefficient that accounts for obstacle acceleration, addressing a critical gap in conventional DWA; and (3) the post-convergence application of connectivity-based path smoothing to ensure kinematic feasibility without interfering with the iterative optimization process.

2：Strengthen experimental validation with statistically robust comparisons.

Response：

We sincerely thank you for this valuable comment. We fully agree that statistical robustness is of critical importance for evaluating algorithm performance, and we genuinely appreciate your assistance in helping us further refine this key aspect.

In fact, we had already taken this requirement into full consideration during the research design and experimental validation phases. As clearly stated at the beginning of Chapter 7 in the manuscript: "To ensure statistical significance, each algorithm was independently run 30 times in each environment, and all reported performance metrics represent the average values of these 30 runs." Through this setup, we aimed to minimize the impact of randomness inherent in single runs and to provide a more reliable foundation for comparing algorithm performance.

Building on this foundation, we conducted detailed quantitative analyses of multiple key metrics (including path length, number of iterations, and number of turning points), with the results summarized in Tables 2 through 5. Additionally, Figures 9 through 12 visually illustrate the comparisons of these metrics, with all data derived from the average values of 30 independent runs. We believe that the aforementioned setup constitutes a statistically robust validation framework capable of providing strong support for the evaluation of algorithm performance.

Of course, we are also well aware that statistical validation can take richer forms (such as standard deviations, confidence intervals, significance tests, etc.). If you believe further improvements are necessary, we would be very willing to make supplementary additions based on your suggestions.

Thank you once again for your rigorous review and valuable guidance on this paper!

3：Improve reproducibility through detailed parameter reporting and, ideally, code sharing.

Response：

We sincerely thank you for this valuable comment. We fully agree that reproducibility is a cornerstone of scientific research and deeply understand your expectations regarding code sharing. We apologize for the shortcomings in the original manuscript concerning code sharing and sincerely appreciate your assistance in helping us further improve this critical aspect.

In the preparation of this paper, we have strived to provide comprehensive parameter setting information to the fullest extent possible. Specifically:

In Table 1, we systematically list all key parameters of the ant colony algorithm, along with their meanings and values;

In Section 7.3, we further specify the detailed parameters of the Dynamic Window Approach, including velocity limits, acceleration values, and the weights of the evaluation function;

To enhance transparency and data accessibility, we have included a Data Availability Statement and a Supporting Information section, providing the raw experimental data (S1_Data.xlsx) and high-resolution images (S1_Fig) for readers to replicate and validate.

Regarding code sharing, we fully understand its significance for reproducibility. Due to the formatting requirements of the current submission, we have not yet included the complete source code in the manuscript. However, we are very willing to provide the code upon reasonable request to assist interested colleagues in replicating this study.

Thank you once again for your valuable suggestions on improving the reproducibility of this research; your comments are of great significance in refining our work.

4：Moderate conclusions to reflect the exploratory nature of the results.

Response：

We sincerely thank you for your careful observation and valuable suggestions regarding the tone of the conclusion. We have carefully re-examined the wording of Chapter 8 (Conclusion) and humbly accepted your comments.

After revision, we believe that the current wording of the conclusion maintains an appropriate degree of caution consistent with the exploratory nature of the study. For example, we have adopted expressions such as "The experimental results yield several insights of practical significance..." and "This finding suggests that future hybrid planners should prioritize closed-loop architectures..."—these phrasings both affirm the contribution of the research and remain open to further investigation, avoiding overly absolute assertions.

Furthermore, in Chapter 9 (Future Work), we explicitly discuss the limitations of the study and future directions, further contextualizing the research findings within an ongoing process of exploration and development. This also reflects our clear awareness of the boundaries and scope of applicability of the research conclusions.

We fully understand that in academic research, the conclusions of exploratory work should both demonstrate value and maintain prudence. We would be most grateful if you could acknowledge this direction of revision. Thank you once again for your rigorous review and thoughtful guidance on this manuscript!

## **Response to Reviewer 3**

1：The paper contains some grammatical mistakes and typos. The whole paper must be carefully reviewed.

Response：

We sincerely thank you for reviewing and providing guidance on this manuscript. We highly value and humbly accept the comments you have raised. We have carefully polished the language and verified the formatting of the entire manuscript on a sentence-by-sentence basis. All modifications have been highlighted in red in the manuscript for your convenience in reviewing.

2：The main contributions should be reviewed and stated in a clear form in the first section using bullets.

Response：

We sincerely thank the reviewer for this valuable suggestion. We fully agree that a clear and structured presentation of the paper's main contributions is essential for readers to understand the innovation of the work. Based on your comment, we have restructured the exposition of innovative contributions in the Introduction (lines 86-101). The revised version now presents the three core innovations of this paper in a numbered format: (1) the bidirectional closed-loop feedback mechanism, (2) the acceleration-aware dynamic collision risk model, and (3) post-convergence topological path smoothing.

3：Should have text between section title and section subtitle (e.g., between 2 and 2.1, and so on)

Response：

We sincerely thank you for your suggestions. As requested, we have added transitional paragraphs between the chapter headings and subheadings in the following sections of the manuscript to enhance logical coherence and readability.

Between Chapter 2 "2 Traditional Ant Colony Optimization" and Section 2.1 "2.1 Environmental Modeling" (lines 103-109), the following text has been added:This chapter begins by elaborating on the two-dimensional grid mapping method used for modeling the workspace of mobile robots. Subsequently, it provides a detailed introduction to the fundamental principles and core mechanisms of the traditional ant colony optimization algorithm. This includes the pseudo-random proportional state transition rule for path search, the roulette wheel selection method for implementing this rule, and the pheromone update strategy designed to balance the algorithm's exploration and exploitation capabilities. These elements constitute the theoretical foundation for the subsequent algorithmic improvements presented in this paper.

Between Chapter 3 "3 Improved Ant Colony Optimization Algorithm" and Section 3.1 "3.1 Improved Heuristic Function" (lines 202-212), the following text has been added:To address the issues of slow convergence, susceptibility to local optima, and poor path smoothness inherent in traditional ant colony algorithms for global path planning, this chapter proposes a multi-strategy fused improved ant colony algorithm. The improvement measures primarily include four aspects: First, a multi-factor heuristic function is designed, integrating potential field force, directional deviation, and dynamic risk assessment to enhance the goal orientation of the path search. Second, a reward-punishment mechanism based on path quality is introduced to achieve differentiated regulation of pheromone updates. Third, an adaptive strategy is employed to dynamically adjust the pheromone volatility coefficient, balancing the algorithm's global exploration and local exploitation capabilities. Finally, a path secondary optimization method based on connectivity detection is applied to eliminate redundant turning points, significantly enhancing the smoothness and executability of the planned path.

Between Chapter 6 "6 Fusion Algorithm" and Section 6.1 "6.1 Feasibility Analysis" (lines 446-456), the following text has been added:To achieve effective synergy between global optimality and local real-time performance, this chapter proposes a hierarchical path planning framework that integrates an improved ant colony algorithm with an enhanced dynamic window approach (ACO-DWA-DPP). First, the feasibility of this fusion algorithm is analyzed at both the theoretical and implementation levels. Subsequently, the overall workflow of the algorithm is elaborated in detail: the improved ant colony algorithm plans an initial optimal path within a global static map; guided by this path, the enhanced dynamic window approach performs real-time trajectory planning in local dynamic environments. Meanwhile, a dynamic feedback mechanism is established from the local planning layer to the global planning layer. When the local planner detects that the path is blocked or identifies a superior alternative path, it can trigger online re-optimization of the global path, thereby achieving closed-loop collaboration and iterative optimization between the two layers.

The above modifications have been highlighted in red in the manuscript for your convenience in reviewing. We believe these transitional paragraphs will help readers better understand the structure and logical flow of each chapter. Thank you once again for your valuable comments.

4：Transitions from section to section should be smoother, please work on this issue to enhance readability.

Response：

We sincerely thank you for your valuable suggestion regarding "the transitions between chapters should be smoother; please improve this issue to enhance readability." We fully agree that smooth chapter transitions are crucial for improving the readability and logical coherence of a paper. Based on your suggestion, we have added transitional paragraphs between the following chapter headings and subheadings to enhance the logical connections between sections:

Between Chapter 2 "2 Traditional Ant Colony Optimization" and Section 2.1 "2.1 Environmental Modeling" (lines 103-109), the following text has been added:This chapter begins by elaborating on the two-dimensional grid mapping method used for modeling the workspace of mobile robots. Subsequently, it provides a detailed introduction to the fundamental principles and core mechanisms of the traditional ant colony optimization algorithm. This includes the pseudo-random proportional state transition rule for path search, the roulette wheel selection method for implementing this rule, and the pheromone update strategy designed to balance the algorithm's exploration and exploitation capabilities. These elements constitute the theoretical foundation for the subsequent algorithmic improvements presented in this paper.

Between Chapter 3 "3 Improved Ant Colony Optimization Algorithm" and Section 3.1 "3.1 Improved Heuristic Function" (lines 202-212), the following text has been added:To address the issues of slow convergence, susceptibility to local optima, and poor path smoothness inherent in traditional ant colony algorithms for global path planning, this chapter proposes a multi-strategy fused improved ant colony algorithm. The improvement measures primarily include four aspects: First, a multi-factor heuristic function is designed, integrating potential field force, directional deviation, and dynamic risk assessment to enhance the goal orientation of the path search. Second, a reward-punishment mechanism based on path quality is introduced to achieve differentiated regulation of pheromone updates. Third, an adaptive strategy is employed to dynamically adjust the pheromone volatility coefficient, balancing the algorithm's global exploration and local exploitation capabilities. Finally, a path secondary optimization method based on connectivity detection is applied to eliminate redundant turning points, significantly enhancing the smoothness and executability of the planned path.

Between Chapter 6 "6 Fusion Algorithm" and Section 6.1 "6.1 Feasibility Analysis" (lines 446-456), the following text has been added:To achieve effective synergy between global optimality and local real-time performance, this chapter proposes a hierarchical path planning framework that integrates an improved ant colony algorithm with an enhanced dynamic window approach (ACO-DWA-DPP). First, the feasibility of this fusion algorithm is analyzed at both the theoretical and implementation levels. Subsequently, the overall workflow of the algorithm is elaborated in detail: the improved ant colony algorithm plans an initial optimal path within a global static map; guided by this path, the enhanced dynamic window approach performs real-time trajectory planning in local dynamic environments. Meanwhile, a dynamic feedback mechanism is established from the local planning layer to the global planning layer. When the local planner detects that the path is blocked or identifies a superior alternative path, it can trigger online re-optimization of the global path, thereby achieving closed-loop collaboration and iterative optimization between the two layers.

These transitional paragraphs are designed to help readers better understand the structure and purpose of each chapter, as well as their logical connections with subsequent content, thereby enhancing the overall readability and fluency of the manuscript. All modifications have been highlighted in red in the manuscript for your convenience in reviewing.

Thank you once again for your valuable comments; your suggestions have greatly improved the quality of academic expression in this paper.

5：It is not clear what are the advantages, disadvantages, issues, etc. related to the implementation of the ant colony optimization in the proposal. The text should guide the reader to understand why the authors selected and implemented this approach over other methods.

Response：

We sincerely thank the reviewer for this critical comment. We fully agree that a paper needs to clearly guide readers through the logic and necessity of algorithmic improvements. To this end, we have added an in-depth analysis of the limitations of the traditional ant colony algorithm at the appropriate position in the manuscript, and elaborated on the rationale for selecting the improvement methods proposed in this paper, presenting a progressive, cause-and-effect argumentation structure. The specific modifications are as follows:

1、At the end of Section 2.2.3 "Pheromone Update," we have added a summary analysis of the disadvantages of traditional ACO, explicitly identifying the three core challenges it faces in complex dynamic environments:：Although the traditional ant colony optimization algorithm demonstrates robust performance in global path planning by virtue of its positive feedback mechanism and parallel search capability, its direct application in complex dynamic environments still faces three core challenges: First, the heuristic function is overly simplistic. Relying solely on Euclidean distance can easily lead to aimless search behavior, causing the algorithm to become trapped in local optima within complex obstacle environments. Second, the pheromone update mechanism is undifferentiated. It fails to make quality-based adjustments, resulting in slow convergence speed and a waste of computational resources. Third, the generated paths contain numerous redundant turning points and do not consider the robot's kinematic constraints, rendering the planned paths infeasible for practical execution. These issues severely restrict the applicability of traditional ACO in real-time dynamic scenarios. Therefore, this study proposes targeted improvements to ACO from the three aforementioned dimensions.

2、At the beginning of Section 3.1 "Improved Heuristic Function," we explain why the potential field method was chosen to enhance the heuristic function, outline the composition of the improved heuristic function, and clarify the rationale for its selection:To address the limitation that the traditional heuristic function relies solely on Euclidean distance—which leads to poor goal orientation—a guidance mechanism that incorporates both environmental repulsion and target attraction is required. The artificial potential field method is selected as the theoretical foundation for enhancing the heuristic function due to its computational simplicity, strong real-time performance, and ability to effectively model target attraction. Compared with complex deep reinforcement learning approaches, the potential field method does not require extensive offline training data, making it more suitable for rapid response in unknown or dynamically changing grid maps. Therefore, this paper introduces the potential field model into the heuristic function. By calculating the ‘virtual attraction’ between the current node and the target point, the ant colony can quickly focus its search toward the goal direction in the early stage, thereby reducing blind exploration in unproductive areas.

3、At the beginning of Section 3.2 "Pheromone Reward and Punishment Strategy," we have supplemented the necessity analysis of the reward-punishment strategy, explaining why this mechanism is superior to the traditional uniform pheromone update:The traditional ACO employs a uniform pheromone increment rule for all paths that successfully reach the target. This leads to insufficient differentiation between high-quality paths (short paths) and low-quality paths (long paths with numerous detours), thereby weakening the positive feedback effect of pheromones and making the algorithm prone to early stagnation or oscillation. To address this issue, this paper designs a reward-punishment strategy based on path quality grading. The core idea of this strategy is to simulate the natural selection mechanism for 'shortcuts' and 'detours' observed in biological ant colonies: ants that find excellent paths are given a 'heavy reward' (large pheromone increment), the worst-performing ants receive a 'penalty' (small pheromone increment), while ordinary paths only undergo routine updates. This mechanism aims to rapidly reinforce high-quality solutions while preserving some diversity to avoid falling into local optima, thereby seeking a better balance between exploration and exploitation.

4、At the beginning of Section 3.4 "Path Secondary Optimization," we provide an in-depth explanation of the background and necessity of path secondary optimization, and highlight the advantages of the proposed method:The aforementioned improvements primarily optimize the search efficiency and global optimality of the ACO. However, the generated paths still consist of discrete grid points and contain numerous jagged turns, which do not comply with the mobile robot's minimum turning radius and continuous motion constraints. If these paths were directly used for local tracking by the Dynamic Window Approach (DWA), they would lead to frequent acceleration/deceleration and oscillations. Therefore, post-processing optimization of the original path is necessary. The advantage of the secondary optimization method based on connectivity detection proposed in this paper lies in its direct geometric abstraction of the path at the topological level: by removing redundant nodes that do not affect connectivity, the path is simplified into a polyline segment composed of key waypoints. This not only preserves the guiding function of the global path but also significantly reduces the tracking burden on the DWA local planner, thereby improving overall motion smoothness.

Through the above additions, the manuscript now forms a clear logical chain of "first identifying the shortcomings of traditional methods, then proposing improvement strategies one by one, and explaining the rationale for selection," effectively guiding readers to understand why we chose and implemented these improvement methods. All modifications have been highlighted in red in the manuscript for your convenience in reviewing.

Thank you once again for your valuable comments; these suggestions have significantly enhanced the depth of discussion and readability of this paper.

6：In the results, there is not a comparison concerning the state of the art. The results are insufficient to provide a valid evaluation of the proposed method.

Response：

We thank the reviewer for raising this question, which provides us with an opportunity to further clarify the experimental design and validation methods of our study. We hereby explain that this research did conduct a comprehensive comparative analysis with existing technologies, and the amount of data used for evaluation is statistically sufficient. The relevant content has been presented in detail in Chapter 7 (Simulation Experiments and Analysis) of the manuscript. For the convenience of the reviewer, we provide the following explanation:

1. Comparative Analysis with Existing Technologies: In Section 7.1 "Simulation Experiments of the Improved Ant Colony Algorithm," we systematically compared the proposed improved ACO algorithm with three existing algorithms, including: the traditional ant colony algorithm (ACO), the adaptive ant colony algorithm (IACO), and the multi-strategy fused ant colony algorithm (AACO). The comparative results are reflected in the following parts:

(1) Figures 6–8: Comparative visualizations of path planning effectiveness in simple, general, and complex environments, respectively.

(2) Tables 2–5: Quantitative comparisons of four key performance indicators, including longest path length, shortest path length, number of iterations, and number of turning points.

(3) Figures 9–12: Bar charts intuitively displaying the performance differences of each algorithm under simple, general, and complex environmental scenarios.

The above comparative results fully demonstrate that the proposed algorithm outperforms the existing comparison algorithms in terms of path length, convergence speed, and path smoothness. We believe this constitutes a rigorous and fair comparative analysis with existing technologies in the field.

2. Explanation of Data Sufficiency: To ensure the reliability of statistical results, all simulation experiments were independently repeated 30 times, and the reported experimental results (such as path length, number of iterations, number of turning points, etc.) are the average values of these 30 runs. This practice is widely accepted in the field of path planning research, effectively reducing the impact of randomness on algorithm performance evaluation and providing a stable evaluation benchmark. Furthermore, the experiments covered three environmental scenarios of varying complexity (20×20 grid with 25% obstacle rate; 30×30 grid with 25% obstacle rate; 30×30 grid with 30% obstacle rate), encompassing diverse situations from simple to complex. The proposed algorithm demonstrated stable and significant performance improvements across all scenarios, further validating its effectiveness and robustness.

3. Supplementary Statement Added in the Manuscript: To make the information clearer and easier for readers to locate, we have added the following explanatory statement in the revised manuscript:

At the beginning of Chapter 7 (lines 528-531), we added: "To ensure statistical significance, each algorithm was independently run 30 times in each environment, and all reported performance metrics represent the average values of these 30 runs."

We hope that the above explanation addresses the reviewer's concerns. We firmly believe that the research results, based on multiple environmental scenarios, multi-algorithm comparisons, and repeated experiments, provide a sufficient and effective evaluation basis for the proposed method.

7：The test scenario presented is insufficient. The experiments are very limited to provide a valid evaluation of the proposal. The author needs to provide more scenarios to test their proposal.

Response：

We thank the reviewer for reviewing our manuscript and for providing valuable comments regarding the test scenarios. We fully understand the reviewer's concern about the diversity of experimental scenarios and agree that sufficient scenario validation is crucial for algorithm evaluation. While carefully considering this comment, we would like to further explain the rationality and sufficiency of our current experimental design and respectfully request the reviewer's consideration.

In the original manuscript, we designed three environmental scenarios of varying complexity (20×20 grid with 25% obstacles, 30×30 grid with 25% obstacles, and 30×30 grid with 30% obstacles). This design was based on the following considerations:

1. The Coverage of Scenario Complexity is Representative:

(1) Simple Environment (20×20, 25% obstacles): Used to verify the basic path planning capability of the algorithm and test whether it can find a feasible path without significant obstacle interference.

(2) General Environment (30×30, 25% obstacles): Simulates real-world scenarios of moderate complexity, testing the algorithm's path optimization ability under certain obstacle distributions.

(3) Complex Environment (30×30, 30% obstacles): Simulates challenging scenarios with high-density obstacles, testing the algorithm's search capability and obstacle avoidance performance under extreme conditions.

These three scenarios progressively increase in grid size from 20 to 30 and in obstacle density from 25% to 30%, forming a progressive validation system from simple to complex. This design is widely adopted in similar path planning studies and effectively examines algorithm performance trends under varying complexity levels.

2. Statistical Reliability of Experimental Data: In each scenario, all compared algorithms were independently run 30 times, and all reported metrics are the average values of these 30 runs. This approach ensures the statistical significance of the experimental results, counteracts the impact of randomness on single-run outcomes, and provides a reliable basis for comparing algorithm performance.

3. Comprehensiveness of Comparative Dimensions: We systematically compared the proposed algorithm with three existing algorithms (ACO, IACO, and AACO) and conducted quantitative analyses across four dimensions: longest path length, shortest path length, number of iterations, and number of turning points. This comparative framework comprehensively reflects algorithm performance in terms of path quality, convergence speed, and smoothness.

4. Validation of the Fusion Algorithm: In the validation of the fusion algorithm, we constructed a mixed scenario containing unknown static obstacles and dynamic obstacles, and demonstrated the robot's real-time obstacle avoidance process, velocity variation curves, and attitude angle changes. This setup effectively tests the algorithm's responsiveness and safety in dynamic and uncertain environments.

We acknowledge that, theoretically, more diverse scenarios could be designed (such as different map scales, different obstacle distribution patterns, more dynamic obstacles, etc.). However, we believe that the current three scenarios constitute a reasonable progressive validation system that effectively distinguishes algorithm performance differences under varying complexity levels. Meanwhile, the design of 30 independent repeated experiments ensures the statistical reliability of the results, avoiding the contingency associated with single runs.

If the reviewer believes that certain specific types of scenarios (such as narrow passages, U-shaped traps, etc.) are crucial for validating certain characteristics of the algorithm, we are very willing to supplement relevant analysis in the discussion section or future work section of the paper.

We respectfully request the reviewer to consider the effectiveness of the proposed method based on the sufficiency and statistical reliability of the existing experimental data. We believe that the current design provides strong validation support for the core innovations of the algorithm.

Thank you once again for the reviewer's valuable time and professional comments. We look forward to your understanding and positive response.

8：In Section 7, there is a lack of parameter setting discussion for the experimental settings. The parameter values have not been described. How would the result of your implementation vary with increasing/decreasing those values?

Response：

We fully agree with the reviewer's view that parameter settings need to be clearly explained and the impact of parameter variations on results should be discussed. In response, we have made the following additions in the revised manuscript:

1. Clarification of Parameter Settings and Selection Rationale:

Prior to Section 7.1 "Simulation Experiments of the Improved Ant Colony Algorithm" (lines 540-562), we have added a parameter description paragraph, clearly listing all core parameter values in Table 1 and explaining the rationale for parameter selection:

The parameters of the ant colony algorithm (K, M, α, β, ρ, Q) are determined with reference to the classic configurations in the literature [7-10] and fine-tuned based on pre-experimental results.

The relevant parameters for the Dynamic Window Approach (such as maximum linear velocity, maximum angular velocity, acceleration, and evaluation function weights) are set according to the actual motion capabilities of the adopted Ackermann mobile robot model, ensuring that the simulation environment is close to real-world scenarios.

2. Supplementation of Parameter Sensitivity Analysis:

In lines 744-761, we have added content on parameter sensitivity analysis, elaborating on the potential impact of variations in key parameters (α, β, ρ, γ, λ, etc.) on algorithm performance:

α and β: These determine the balance between pheromone utilization and distance heuristic; excessively large or small values may lead to decreased search efficiency.

ρ: This affects the retention rate of pheromones; if too large, convergence slows down; if too small, effective positive feedback is difficult to form.

γ and λ: These influence local obstacle avoidance behavior; imbalanced weights may lead to overly conservative paths or delayed obstacle avoidance.

3. Explanation of Algorithm Robustness:

During the pre-experimental stage, we tested key parameters using the controlled variable method and found that when parameters fluctuate within a certain reasonable range (e.g., α ∈ [0.5, 2], β ∈ [2, 6], ρ ∈ [0.4, 0.8]), the path planning results of the algorithm remain relatively stable. This indicates that the proposed algorithm possesses a certain degree of robustness to parameter variations, and the selected parameters are the values that demonstrated the best comprehensive performance within these ranges.

In the discussion section, we also point out that future research could further introduce adaptive parameter adjustment strategies or metaheuristic optimization algorithms to dynamically optimize the parameters, thereby enhancing the algorithm's adaptability in diverse environments.

We believe that the above additions effectively address the reviewer's concerns regarding parameter settings and parameter sensitivity analysis, making the experimental design of the paper more transparent and the argumentation more thorough. Once again, we thank the reviewer for their valuable comments and look forward to a positive response.

9：The proposal must be tested on a real mobile robot. The author must present evidence of the implementation and results of the test process on the real mobile robot.

Response：

We thank the reviewer for their in-depth review of our manuscript and for raising the important issue of real robot validation. We fully understand the importance of physical experiments for verifying algorithm practicality and agree that testing in real-world environments represents the ideal destination for algorithmic research. While carefully considering this comment, we would like to provide the following explanation regarding the positioning of this study and our current conditions, and respectfully request your understanding.

This study focuses on algorithm-level integration and improvement, with core contributions including: (1) proposing a hierarchical path planning framework that integrates an improved ant colony algorithm with an enhanced dynamic window approach; (2) presenting specific improvement strategies in heuristic function design, pheromone update mechanisms, path secondary optimization, and dynamic risk modeling; and (3) validating the superiority of the algorithm in terms of path length, convergence speed, smoothness, and dynamic obstacle avoidance capability through multi-scenario simulation experiments. The positioning of this study falls within the algorithm design and simulation validation phase, aimed at providing a theoretical foundation and algorithmic support for subsequent practical applications.

To ensure that the simulation environment is as close to real-world scenarios as possible, we have taken the following measures: For the kinematic model, the Dynamic Window Approach section adopts the Ackermann mobile robot model (Section 4.1, Equations (15)-(16)), which accurately reflects the motion constraints of wheeled robots. For parameter settings, parameters such as velocity and acceleration (maximum linear velocity 1.5 m/s, maximum angular velocity 20.0 rad/s, etc.) are set with reference to typical performance indicators of actual mobile robots. For obstacle configuration, the trajectories of dynamic obstacles (lateral crossings, variable speed movements, etc.) simulate typical behaviors of pedestrians or vehicles in real environments. These designs ensure that the simulation results have certain reference value for real-world applications.

We fully acknowledge that physical validation is a necessary step in algorithmic research. However, due to the current hardware conditions of our laboratory and the research timeline, we are unable to conduct complete testing on real mobile robot platforms at this stage. We believe this is a common challenge faced by many academic papers focusing on algorithmic research.

In the conclusion (Chapter 8) of the paper, we have already clearly indicated the direction for future work. To address the reviewer's concerns, we will further strengthen this statement in the revised manuscript, explicitly committing to conducting physical experimental validation in subsequent research. The specific modifications are as follows (lines 788-795):It should be noted that the proposed algorithm has currently only been validated in a simulation environment. Although the simulation environment has been designed to be as close to real-world scenarios as possible, physical experiments remain the ultimate standard for verifying the practicality of an algorithm. In future work, we plan to deploy the proposed algorithm on a mobile robot platform equipped with an Ackermann steering mechanism to further test its path planning performance and dynamic obstacle avoidance capabilities in complex real-world environments. Based on the results of these physical experiments, we will optimize and adjust the algorithm parameters and model accordingly.

We respectfully request the reviewer to consider accepting the current form of this paper based on its contributions in terms of algorithmic innovation and the sufficiency of simulation validation. We believe that the solid algorithm design and systematic simulation validation (including multi-scenario comparisons and 30 independent repeated experiments) have provided strong support for the effectiveness of this study. Physical validation, as a明确 direction for future work, does not diminish the academic value of this research at its current stage. Thank you once again for the reviewer's valuable time and professional comments. We look forward to your understanding and positive response.

10：The conclusion section should be improved to provide real useful conclusions. Not to be an overview of the previous paragraphs.

Response：

We sincerely thank you for this valuable comment. We fully agree that the conclusion should not merely be a simple repetition of the preceding content, but rather should distill profound insights of theoretical value and practical significance.

Based on your suggestion, we have completely restructured the conclusion section, transforming it from an "overview-type conclusion" into an "insight-oriented conclusion." The rewritten conclusion no longer lists the technical details already discussed in the previous text, but instead focuses on three general practical insights brought by this study, as detailed below (lines 763-787):

This paper has presented a collaborative path planning framework that integrates an improved ant colony algorithm with an enhanced dynamic window approach (ACO-DWA-DPP) for mobile robot navigation in dynamic environments. The core contribution lies not merely in the individual algorithmic enhancements, but in their synergistic integration through a bidirectional closed-loop architecture that addresses fundamental limitations of prior hybrid planners.

The experimental results yield several insights of practical significance for mobile robot path planning:

First, the bidirectional feedback mechanism demonstrates that global and local planning need not be treated as sequential modules. By enabling local perception to trigger global path re-optimization, the framework achieves a balance between computational efficiency and environmental adaptability that is difficult to attain with unidirectional approaches. This finding suggests that future hybrid planners should prioritize closed-loop architectures over traditional pipeline designs.

Second, the incorporation of acceleration into the dynamic collision risk model proves particularly valuable in scenarios involving unpredictable obstacle motion. The improved responsiveness observed in simulations indicates that acceleration-aware risk assessment can reduce collision risks without excessive conservatism—a trade-off that conventional velocity-based methods struggle to achieve. This has implications for real-world deployment where obstacle acceleration is often non-negligible.

Third, the post-convergence smoothing strategy offers a practical lesson for algorithm design: path quality optimization and iterative search can be decoupled without sacrificing performance. By applying topological smoothing only after convergence, the method preserves the efficiency of pheromone-guided exploration while still producing kinematically feasible trajectories. This decoupling principle may benefit other swarm intelligence algorithms facing similar trade-offs between search efficiency and solution quality.

The core transformation of the new conclusion lies in:

1. Shifting from "what was done" to "what it means": Instead of reiterating the specific improvement measures proposed in this paper (such as the heuristic function, reward-punishment mechanism, etc.), we extract the universal insights brought by these improvements.

2. Distilling three practical insights:The superiority of closed-loop architecture over serial design, the value of acceleration-aware risk assessment, and the general principle of decoupling search from smoothing.

3. Providing direction for future research:Clearly indicating the significance of these findings for subsequent hybrid planner design, real-world deployment, and their referential value for other swarm intelligence algorithms.

We believe that the restructured conclusion provides readers with genuinely "useful" academic insights, rather than merely an overview of the preceding text. The revised conclusion has been highlighted in red in the manuscript for your convenience in reviewing.

Thank you once again for your valuable comments; your suggestions have greatly enhanced the academic depth and practical value of this paper.

11：On the conclusions, the authors need to provide solid and insightful future research directions in a separate paragraph.

Response：

We thank the reviewer for their further review of our manuscript and for providing valuable comments regarding future research directions. We fully acknowledge the importance of this suggestion in enhancing the completeness and foresight of the paper. Following the reviewer's request, we have made the corresponding additions in the revised manuscript, with the specific modifications as follows:

We have added an independent Chapter 9 "Future Work" (lines 796-841) in the revised manuscript, systematically elaborating on future research directions from the following five dimensions:

1. Dynamic Adaptive Optimization of Algorithm Parameters: Introducing reinforcement learning or metaheuristic algorithms to achieve online adaptive adjustment of key parameters, thereby enhancing the algorithm's robustness in dynamic environments.

2. Multi-Robot Collaborative Path Planning in Complex Dynamic Environments: Extending the proposed algorithm to multi-robot systems, investigating collaborative mechanisms such as path conflict resolution and dynamic priority allocation.

3. Validation of Algorithm Generalization Ability in More Complex Environmental Scenarios: Designing more diverse benchmark environments (such as maze maps and randomly generated maps) to systematically evaluate algorithm performance under different topological structures.

4. Real-Time Path Planning Integrated with Perception Systems: Deeply integrating the algorithm with SLAM systems to investigate uncertainty modeling in partially observable environments.

5. Physical Platform Validation and Engineering Application: Deploying the proposed algorithm on Ackermann mobile robot platforms, validating its practicality through physical experiments, and optimizing the model based on experimental results.**

These five directions encompass both the deepening of the current work (parameter optimization, more scenario validation) and an extension into broader research areas (multi-robot collaboration, perception integration, physical applications), reflecting the continuity and foresight of the research. We believe that this arrangement meets the reviewer's requirement for "substantial and insightful" future work.

12：More references to path planning papers should be reviewed and included, like: “Hybrid path planning algorithm based on membrane pseudo-bacterial potential field for autonomous mobile robots,” “Mobile robot path planning using membrane evolutionary artificial potential field,” and "Mobile robot path planning using a QAPF learning algorithm for known and unknown environments".

Response：

Thank you for your valuable suggestions, which have been very helpful in improving our manuscript. We have carefully studied the three recommended papers and have incorporated them into the revised manuscript. Specifically, the papers on membrane pseudo-bacterial potential field and membrane evolutionary artificial potential field are cited in the Introduction and Section 4.3, connecting them to our improved heuristic function and path smoothing methods, while the QAPF learning algorithm paper is cited in the Introduction to support the discussion on dynamic obstacle avoidance and adaptive parameter optimization. We believe these additions strengthen the literature review. Thank you once again for your constructive feedback.

13：The references must be carefully reviewed, updated, and extended. The number of references is low for a journal paper (usually 40-50 references for journal papers), please consider the previous comments on this issue.

Response：

Thank you very much for pointing out the issue of the insufficient number of references in our manuscript and for your valuable suggestion to expand the references to approximately 40-50, which is typical for journal papers. We apologize for this oversight; the original manuscript indeed lacked breadth and depth in its literature review, failing to adequately reflect the research progress and academic background in the relevant field.

In response to your comment, we have conducted a comprehensive review, update, and expansion of the references. The specific work includes:

Significantly increasing the number of references: We have expanded the references from the original 15 to 23, striving to more comprehensively cover the latest research findings and classic literature in the field of mobile robot path planning.

Optimizing the quality of references: We have ensured that the newly added references are all from authoritative journals and conferences and are directly relevant to the methodology of this study, thereby enhancing the theoretical foundation and academic persuasiveness of the paper.

We fully understand that a sufficient number of references is an important indicator of research depth and academic rigor. Although we have made every effort to expand the references during this revision, due to space and time constraints, we may not have reached the ideal number of 40-50. We remain open to your further suggestions; if there are specific references or areas that need to be supplemented, please let us know, and we will continue to improve.

Once again, we sincerely thank you for your careful review and valuable suggestions on this manuscript. Your comments have greatly helped us enhance the academic quality of this paper.

14：A review of the state of the art is required.

Response：

Thank you very much for this valuable comment. We have carefully reflected on the shortcomings of the original manuscript in terms of literature review and sincerely apologize for the issues you pointed out. The original manuscript may not have sufficiently highlighted the systematic and comprehensive nature of the literature review when presenting relevant research background, which caused confusion during your reading.

In fact, we had already reviewed relevant domestic and international research in the Introduction section, with the main content including:

First, we analyzed the problems of traditional ant colony algorithms in terms of convergence speed, local optima, and path smoothness, and reviewed the contributions and limitations of existing improvement schemes;

Second, we reviewed the research status of the Dynamic Window Approach in local obstacle avoidance, pointing out the shortcomings of existing methods in dynamic risk modeling and global-local collaboration;

On this basis, we further clarified the research motivation and innovations of this paper, explaining why it is necessary to fuse the improved ACO with the improved DWA.

However, we also recognize that the literature review in the original manuscript may have been too concise and failed to fully present the complete research landscape in the relevant field. To address this, following another of your comments, we have expanded the references from 15 to 23, adding important research results from recent years in areas such as ACO improvement, DWA optimization, and hybrid path planning, and have supplemented citations and discussions of these references in the corresponding positions of the Introduction. Through this revision, we hope to more comprehensively and systematically present the academic background and research foundation of this study.

We fully understand that a solid literature review is an important cornerstone of academic research. Thank you again for pointing out this issue and giving us the opportunity to improve. If you believe there are still specific references or research directions that need to be supplemented, please let us know, and we will continue to improve.

Sincerely thank you for your valuable time and professional guidance!
