# Supplementary material for: Path planning for mobile robots by fusing ant colony optimization and dynamic window approach
Source: PLoS One. 2026 Jul 27;21(7):e0340336. doi: 10.1371/journal.pone.0340336 (PMC13405125; doi:10.1371/journal.pone.0340336)
Supplement: Attachment — Submitted filename: Response_to_Reviewers_auresp_2.docx [file pone.0340336.s004.docx]

Dear Editor and Reviewers:

Thank you very much for taking the time to review our manuscript (Manuscript ID: PONE-D-25-67110) and for providing valuable and constructive comments. These insightful suggestions have been very helpful in improving the quality, rigor, and clarity of our manuscript. We have carefully considered all comments and revised the manuscript accordingly. Below, we provide a point-by-point response to the comments raised by the Editor and Reviewers, together with a detailed explanation of the corresponding revisions. All changes have been highlighted in red in the revised manuscript for your convenience. We sincerely appreciate your careful evaluation and thoughtful guidance.

**Response to Editor**

1.Supplement Tables 2 through 5 with standard deviation values for all reported metrics. Apply non-parametric pairwise significance testing to each algorithm comparison and report the resulting test statistics and p-values.

Response: Thank you for this important comment. We agree that mean-only reporting is insufficient for stochastic population-based algorithms. In the revised manuscript, Tables 2–5 have been supplemented with standard deviation values, and the results are now reported as mean ± standard deviation. In addition, we conducted pairwise Wilcoxon signed-rank tests between the proposed algorithm and each baseline algorithm using the 30 independent runs. To avoid making the main text overly lengthy, the test statistic W and the corresponding p-value for each comparison are reported in S2_Table. We also added a brief description of the statistical testing procedure in Section 7.

Thank you once again for your time and valuable comments.

2.Correct the dimensional error in Equation (22) by replacing $y_{r}$ with $v_{r}$ in the relative velocity expression, and reconfirm that all simulation results are based on the corrected formulation.

Response: Thank you for identifying this dimensional inconsistency in Equation (22). We agree that the original term involving $y_{r}\sin(\theta_{r})$ was inappropriate because $y_{r}$ represents a position coordinate rather than a velocity component. In the revised manuscript, we corrected this term to $v_{r}\sin(\theta_{r})$, so that the relative velocity expression is dimensionally consistent. We also rechecked the subsequent calculation of the collision time and the dynamic collision-risk coefficient based on the corrected formulation. The revised Equation (22) now uses the velocity components of the mobile robot and the obstacle, which makes the dynamic risk evaluation physically consistent.

Thank you once again for your time and valuable comments.

3.Correct or provide a rigorous mathematical justification for the segment intersection criterion in Equation (14).

Response: Thank you very much for this insightful comment. We agree that the original formulation based on a single cross-product sign was insufficient for rigorous bounded line-segment intersection detection. Following the reviewer’s suggestion, we have revised the segment intersection criterion in Section 3.4 by adopting the standard cross-product orientation test combined with overlap verification.

Specifically, the original simplified criterion has been replaced with a geometrically rigorous intersection determination method based on four orientation values:

$$\begin{matrix} o_{1} & =cross(A,B,C) \quad o_{2}=cross(A,B,D) \\ o_{3} & =cross(C,D,A)\quad o_{4}=cross(C,D,B) \end{matrix}$$

The intersection condition is now expressed as:

$$AB\cap CD\neq\emptyset\Longleftrightarrow o_{1}o_{2}\leq0\wedge o_{3}o_{4}\leq0\wedge B_{AB,CD}=1$$

where$B_{AB,CD}=1$ indicates that the projections of the two line segments overlap in both the x- and y-directions.

In addition, Equation (13) and the related textual descriptions in Section 3.4 have been revised accordingly to ensure mathematical correctness and consistency with standard computational geometry methods.

These revisions improve the rigor and reliability of the path-pruning process without affecting the overall conclusions of the study.

Thank you once again for your time and valuable comments.

4.Specify rho_max and rho_min in Table 1 or in the parameter description accompanying Equation (12).

Response:Thank you for pointing out this omission. We agree that the parameters in the adaptive pheromone evaporation coefficient should be explicitly specified to ensure the reproducibility of Equation (12). In the revised manuscript, we clarified that the original value “Rho = 0.6” in Table 1 represents the initial pheromone evaporation coefficient, denoted as $\rho_{1}$, rather than a fixed evaporation coefficient. We also added the lower and upper bounds of the adaptive evaporation coefficient, namely $\rho_{\min}=0.1$ and $\rho_{\max}=0.9$, in both Section 3.3 and Table 1. This revision makes the effective range of $\rho$ during the iterative process clear and reproducible.

Thank you once again for your time and valuable comments.

5.Conduct a full audit of all in-text equation cross-references and correct those that do not correspond to the rendered equation numbers.

Response: Thank you for this helpful comment. We have conducted a full audit of all in-text equation cross-references in the manuscript. The incorrect references in Section 3.1 have been corrected: the distance calculation is now referred to as Equation (7) instead of Equation (11), and the improved heuristic function is now referred to as Equation (9) instead of Equation (13). In addition, we removed the duplicated “(12):” text following the reference to Equation (12) and standardized the wording around Equation (13) in Section 3.4. All remaining in-text equation references were checked and confirmed to be consistent with the rendered equation numbers.

Thank you once again for your time and valuable comments.

6.Provide either a quantitative experimental comparison with Xue et al. (2025, Reference [22]) or a detailed technical comparison in Section 6.1.

Response: Thank you for this valuable suggestion. We agree that the relationship between the proposed method and the closely related hybrid ACO-DWA method by Xue et al. [22] should be clarified. Since the method of Xue et al. was developed for unmanned surface vehicles, whereas this study focuses on mobile robots with Ackermann steering constraints in grid-based ground environments, a direct quantitative comparison under the current experimental setting would not be fully consistent. Therefore, following the reviewer’s suggestion, we added a detailed technical comparison in Section 6.1.

In the revised manuscript, we clarified that existing ACO-DWA fusion methods, such as Xue et al. [22], generally use the ACO-generated global path as one-way guidance for DWA-based local obstacle avoidance. In contrast, the proposed ACO-DWA-DPP framework introduces a bidirectional closed-loop feedback mechanism, in which newly detected obstacles and path blockage information from the DWA layer are fed back to the ACO layer for pheromone redistribution and global path re-optimization. We also highlighted the additional differences, including the acceleration-aware dynamic collision risk coefficient and the post-convergence connectivity-based secondary optimization. These revisions clarify the novelty of the proposed framework compared with existing ACO-DWA fusion approaches.

Thank you once again for your time and valuable comments.

7.Expand the dynamic obstacle validation to include at least two simultaneously moving obstacles, or explicitly acknowledge this limitation in the Conclusion.

Response: Thank you for this valuable suggestion. We agree that a single dynamic obstacle is not sufficient to fully stress-test the proposed acceleration-aware collision-risk model or the bidirectional feedback mechanism in highly dynamic environments. Due to the current experimental scope, we are unable to add a complete multi-dynamic-obstacle experiment in this revision. Therefore, following the reviewer’s suggestion, we have explicitly acknowledged this limitation in the revised manuscript.

Specifically, we added a clarification in Section 7.3 that the current validation mainly evaluates the algorithm’s response to one moving obstacle combined with multiple unknown static obstacles. We also added a limitation statement in the Conclusion, noting that more complex scenarios involving multiple simultaneously moving obstacles with different velocities, accelerations, and motion directions will be prioritized in future work. This revision more accurately describes the scope of the current validation and avoids overgeneralizing the dynamic obstacle avoidance results.

Thank you once again for your time and valuable comments.

8.Replace the unresolved citation placeholder '[citations]' at line 460 with appropriate references.

Response: Thank you for pointing this out. We have corrected the unresolved citation placeholder in Section 6.1. The original placeholder “[citations]” has been removed and replaced with the specific reference to Xue et al. [22], which is the most closely related ACO-DWA fusion method discussed in the manuscript. The revised text now properly cites the relevant prior work when discussing existing ACO-DWA fusion approaches.

Thank you once again for your time and valuable comments.

9. Include a technical justification for applying the standard DWA velocity sampling constraints to the Ackermann kinematic model.

Response: Thank you for this important comment. We agree that the use of the standard DWA velocity sampling framework for an Ackermann mobile robot should be clarified. In the revised manuscript, we added a brief technical explanation after Equation (16), stating that the Ackermann mobile robot is simplified as a kinematically equivalent unicycle model at the vehicle reference point during the DWA-based local planning stage. Under this representation, $v$ denotes the forward velocity and $\omega$ denotes the heading-rate of the robot, rather than an independently controllable in-place rotational command. We also clarified that infeasible motions such as in-place rotation are excluded during velocity sampling. In addition, we revised the wording in Section 4.2 by replacing the original reference to a “differential-drive robot” with the Ackermann mobile robot under the equivalent unicycle representation.

Thank you once again for your time and valuable comments.

10.Report average wall-clock execution times or provide a computational complexity analysis for the proposed and baseline algorithms.

Response: Thank you for this helpful comment. We agree that iteration count alone cannot fully represent the overall computational efficiency of the algorithm. To avoid introducing unsupported runtime data, we added a concise computational complexity discussion in Section 7.2. In the revised manuscript, we clarified that the proposed improvements, including the improved heuristic function, pheromone reward-punishment strategy, adaptive evaporation mechanism, path secondary optimization, and dynamic collision-risk evaluation, increase the computational burden of a single planning cycle to some extent. We also explained that the substantial reduction in convergence iterations reported in Table 4 helps compensate for this additional overhead, and that the path secondary optimization is performed only after ACO convergence rather than during every iteration.

Thank you once again for your time and valuable comments.

11.Correct the Figure 8 caption to read 'Comparison of Path Planning in Complex Environments.'

Response: Thank you for pointing out this caption error. We have corrected the caption of Figure 8 in the revised manuscript. The original caption “Comparison of Path Planning in General Environments” has been revised to “Comparison of Path Planning in Complex Environments” to make it consistent with the corresponding experimental scenario described in Section 7.1.

Thank you once again for your time and valuable comments.

12.Correct the double-period error at line 24 of the abstract.

Response: Thank you for pointing out this typographical error. We have corrected the double-period error in the Abstract by replacing “robots..” with “robots.” in the revised manuscript.

Thank you once again for your time and valuable comments.

13.Revise the Acknowledgments section to remove the reference to a listed co-author.

Response: Thank you for pointing this out. We have revised the Acknowledgments section to remove the reference to Prof. Guozhu Song, who is already listed as a co-author of the manuscript. The revised Acknowledgments section now only thanks the laboratory colleagues and fellow students for their support and discussions.

Thank you once again for your time and valuable comments.

14.Include quantitative results from the parameter sensitivity analysis to support the robustness claim at lines 550 through 556.

Response: Thank you for this constructive suggestion. We agree that quantitative parameter sensitivity results would provide stronger support for the robustness claim. Since a complete quantitative sensitivity analysis over all parameter combinations was not included in the current experimental design, we revised the related statement in the manuscript to avoid overgeneralizing the robustness conclusion. Specifically, we changed the description of the parameter sensitivity analysis to a preliminary parameter adjustment using the controlled variable method and added that a more systematic quantitative sensitivity analysis will be conducted in future work. This revision makes the discussion more accurate and avoids unsupported claims.

Thank you once again for your time and valuable comments.

15.Include a scalability discussion in the Conclusion or Future Work sections.

Response:Thank you for this helpful suggestion. We agree that the scalability of the proposed framework should be discussed, since the current simulations are conducted on 20 × 20 and 30 × 30 grid maps. In the revised manuscript, we added a scalability discussion in the Future Work section. We clarified that larger maps may increase the computational cost of ant colony search and the interaction overhead between the global and local planning layers. We also stated that future work will evaluate the proposed framework on larger-scale grid maps and explore efficiency-improvement strategies such as hierarchical planning, map decomposition, and adaptive search-space reduction.

We sincerely thank the Editor and Reviewers again for their careful evaluation of our manuscript and for the constructive comments and suggestions. These comments have helped us improve the rigor, clarity, and completeness of the manuscript. We have revised the manuscript carefully according to all comments and hope that the revised version satisfactorily addresses the concerns raised. We appreciate your time and consideration.

Sincerely,

The Authors
