# Supplementary material for: Path planning for mobile robots by fusing ant colony optimization and dynamic window approach
Source: PLoS One. 2026 Jul 27;21(7):e0340336. doi: 10.1371/journal.pone.0340336 (PMC13405125; doi:10.1371/journal.pone.0340336)
Supplement: Peer review history

# Peer review: Decision Letter 0

**DOI:** 10.1371/journal.pone.0340336.r001

**Authors:** Ravneil Nand

27 Feb 2026

PONE-D-25-67110

Path Planning for Mobile Robots by Fusing Ant Colony Optimization and Dynamic Window Approach

PLOS One

Dear Dr. Guozhu,

Thank you for submitting your manuscript to PLOS ONE. After careful consideration, we feel that it has merit but does not fully meet PLOS ONE’s publication criteria as it currently stands. Therefore, we invite you to submit a revised version of the manuscript that addresses the points raised during the review process.

Please submit your revised manuscript by Apr 12 2026 11:59PM. If you will need more time than this to complete your revisions, please reply to this message or contact the journal office at plosone@plos.org. When you're ready to submit your revision, log on to https://www.editorialmanager.com/pone/ and select the 'Submissions Needing Revision' folder to locate your manuscript file.

Please include the following items when submitting your revised manuscript:

- A letter that responds to each point raised by the academic editor and reviewer(s). You should upload this letter as a separate file labeled 'Response to Reviewers'.
- A marked-up copy of your manuscript that highlights changes made to the original version. You should upload this as a separate file labeled 'Revised Manuscript with Track Changes'.
- An unmarked version of your revised paper without tracked changes. You should upload this as a separate file labeled 'Manuscript'.

If you would like to make changes to your financial disclosure, please include your updated statement in your cover letter. Guidelines for resubmitting your figure files are available below the reviewer comments at the end of this letter.

If applicable, we recommend that you deposit your laboratory protocols in protocols.io to enhance the reproducibility of your results. Protocols.io assigns your protocol its own identifier (DOI) so that it can be cited independently in the future. For instructions see: https://journals.plos.org/plosone/s/submission-guidelines#loc-laboratory-protocols. Additionally, PLOS ONE offers an option for publishing peer-reviewed Lab Protocol articles, which describe protocols hosted on protocols.io. Read more information on sharing protocols at https://plos.org/protocols?utm_medium=editorial-email&utm_source=authorletters&utm_campaign=protocols.

We look forward to receiving your revised manuscript.

Kind regards,

Ravneil Nand

Academic Editor

PLOS One

Journal Requirements:

When submitting your revision, we need you to address these additional requirements.

1.Please ensure that your manuscript meets PLOS ONE's style requirements, including those for file naming. The PLOS ONE style templates can be found at

https://journals.plos.org/plosone/s/file?id=wjVg/PLOSOne_formatting_sample_main_body.pdf and

https://journals.plos.org/plosone/s/file?id=ba62/PLOSOne_formatting_sample_title_authors_affiliations.pdf

2. Please note that PLOS One has specific guidelines on code sharing for submissions in which author-generated code underpins the findings in the manuscript. In these cases, we expect all author-generated code to be made available without restrictions upon publication of the work. Please review our guidelines at https://journals.plos.org/plosone/s/materials-and-software-sharing#loc-sharing-code and ensure that your code is shared in a way that follows best practice and facilitates reproducibility and reuse.

3. Please note that funding information should not appear in any section or other areas of your manuscript. We will only publish funding information present in the Funding Statement section of the online submission form. Please remove any funding-related text from the manuscript.

4. Thank you for stating the following financial disclosure:

“This work was supported by the Key Research and Development Project of the Shanxi Provincial Department of Science and Technology (202102140601015), the 2023 Scientific and Technological Innovation Project of Jinzhong National Agricultural High-tech Zone (Taigu National Science and Technology Innovation Center) (JZNGQBSGZZ004), and the Shanxi Provincial Basic Research Program (202403021222112).”

Please state what role the funders took in the study.  If the funders had no role, please state: "The funders had no role in study design, data collection and analysis, decision to publish, or preparation of the manuscript."

If this statement is not correct you must amend it as needed.

Please include this amended Role of Funder statement in your cover letter; we will change the online submission form on your behalf.

5. Please ensure that you refer to Figure 5 in your text as, if accepted, production will need this reference to link the reader to the figure.

6. Please include captions for your Supporting Information files at the end of your manuscript, and update any in-text citations to match accordingly. Please see our Supporting Information guidelines for more information: http://journals.plos.org/plosone/s/supporting-information.

7. Please remove your figures from within your manuscript file, leaving only the individual TIFF/EPS image files, uploaded separately. These will be automatically included in the reviewers’ PDF.

8. Thank you for providing your underlying data as Supporting Information.

We note that the data set contains text or data that is not in English. Please note that PLOS is an English-language publisher, so we require data sets to be provided in English as well. Please upload an English-language version of your data set.

This will also allow us to determine if your data follows PLOS standards per our Data Availability policy here: https://journals.plos.org/plosone/s/data-availability

9. If the reviewer comments include a recommendation to cite specific previously published works, please review and evaluate these publications to determine whether they are relevant and should be cited. There is no requirement to cite these works unless the editor has indicated otherwise.

10. Please review your reference list to ensure that it is complete and correct. If you have cited papers that have been retracted, please include the rationale for doing so in the manuscript text, or remove these references and replace them with relevant current references. Any changes to the reference list should be mentioned in the rebuttal letter that accompanies your revised manuscript. If you need to cite a retracted article, indicate the article’s retracted status in the References list and also include a citation and full reference for the retraction notice.

Additional Editor Comments:

Dear Authors,

Please work on the comments by the reviewers to improve the paper for publication.

Regards

Academic Editor

[Note: HTML markup is below. Please do not edit.]

Reviewers' comments:

Reviewer's Responses to Questions

**Comments to the Author**

1. Is the manuscript technically sound, and do the data support the conclusions?

The manuscript must describe a technically sound piece of scientific research with data that supports the conclusions. Experiments must have been conducted rigorously, with appropriate controls, replication, and sample sizes. The conclusions must be drawn appropriately based on the data presented.

Reviewer #1: Yes

Reviewer #2: Yes

Reviewer #3: Partly

**********

2. Has the statistical analysis been performed appropriately and rigorously?

Reviewer #1: No

Reviewer #2: No

Reviewer #3: No

**********

3. Have the authors made all data underlying the findings in their manuscript fully available?

The PLOS Data policy requires authors to make all data underlying the findings described in their manuscript fully available without restriction, with rare exception (please refer to the Data Availability Statement in the manuscript PDF file). The data should be provided as part of the manuscript or its supporting information, or deposited to a public repository. For example, in addition to summary statistics, the data points behind means, medians and variance measures should be available. If there are restrictions on publicly sharing data—e.g. participant privacy or use of data from a third party—those must be specified.

Reviewer #1: Yes

Reviewer #2: Yes

Reviewer #3: No

**********

4. Is the manuscript presented in an intelligible fashion and written in standard English?

PLOS ONE does not copyedit accepted manuscripts, so the language in submitted articles must be clear, correct, and unambiguous. Any typographical or grammatical errors should be corrected at revision, so please note any specific errors here.

Reviewer #1: Yes

Reviewer #2: Yes

Reviewer #3: No

**********

5. Review Comments to the Author

Please use the space provided to explain your answers to the questions above. You may also include additional comments for the author, including concerns about dual publication, research ethics, or publication ethics. (Please upload your review as an attachment if it exceeds 20,000 characters)

Reviewer #1: The authors propose a hybrid path planning method that integrates an enhanced ant colony optimization algorithm with a modified dynamic window approach for mobile robots operating in dynamic environments.This manuscript needs minor revision.

(1)The abstract claims the algorithm reduces the longest path length by 41.26%~48.28% compared to traditional ACO, but Table 2 shows AACO actually produces much longer paths than ACO in some cases, which undermines the validity of this comparison baseline.

(2)Equation (8) defines attractive force, which contradicts standard potential field theory where attraction typically increases as distance decreases; Pls check all the equations and definitions.

(3)The paper states that redundant turning points are removed via connectivity checks, but it doesn't clarify whether this post-processing step is applied after every ACO iteration or only once at the end, affecting computational cost claims.

(4)The evaluation function in Equation (25) adds D_dynamic as a positive term, yet higher collision risk should penalize trajectories—this suggests a possible sign error or misinterpretation of the risk coefficient's role.

(5)The claim that the fusion algorithm detects unknown obstacles in real time isn't substantiated by any sensor model or perception module description, making the “real-time detection” assertion appear unsupported.

(6)For path planning applications, the author could consider more articles: 3D vision technologies for a self-developed structural external crack damage recognition robot; Automation in Construction.

Reviewer #2: General Assessment

This manuscript proposes a hybrid path-planning framework that integrates Ant Colony Optimization (ACO) for global path planning with the Dynamic Window Approach (DWA) for local obstacle avoidance and motion control. The motivation to combine the global optimality and exploration strengths of ACO with the real-time feasibility and safety guarantees of DWA is well established and relevant to autonomous mobile robotics.

The paper is technically coherent, addresses a well-defined problem, and demonstrates the feasibility of the proposed approach through simulation-based experiments. The manuscript fits within the scope of PLOS ONE, which prioritizes technical soundness and reproducibility over claims of radical novelty.

However, while the work is competently executed, it currently falls short of the rigor and clarity needed to fully justify publication without revision. The main concerns relate to novelty positioning, experimental validation depth, and statistical robustness of performance claims.

1. Technical Soundness and Validity of Conclusions

The manuscript describes a technically sound approach:

The separation of global and local planning roles between ACO and DWA is conceptually correct.

The algorithmic flow is logically structured.

The mathematical formulations of both ACO pheromone updating and DWA velocity-space evaluation are consistent with established literature.

The simulation results generally support the claim that the fused approach improves path smoothness and collision avoidance relative to single-method planners.

That said, the conclusions occasionally extend beyond what the presented data can strictly support. Performance improvements are reported largely through illustrative comparisons rather than through rigorous quantitative analysis across multiple scenarios and randomized trials.

Assessment:

Technically sound, but conclusions should be more cautiously framed and better supported by systematic evidence.

2. Novelty and Contribution to State-of-the-Art

The idea of combining a global metaheuristic planner (ACO) with a local reactive planner (DWA) is not fundamentally new; similar hybrid global–local planning architectures have been explored extensively in the robotics literature using A*, GA, PSO, RRT variants, and other heuristics.

The contribution of this paper therefore lies not in conceptual novelty, but in:

a specific integration strategy between ACO and DWA,

parameter coordination between the two layers, and

empirical demonstration of feasibility.

However, the manuscript does not sufficiently distinguish its approach from prior hybrid planners nor clearly articulate what is substantively new beyond implementation-level integration.

Recommendation:

The authors should strengthen the literature review and explicitly position their contribution relative to existing ACO-based and DWA-based hybrid methods, clarifying whether the novelty lies in algorithmic coupling, parameter design, or performance characteristics.

3. Statistical Rigor and Experimental Design

The experimental validation is one of the weaker aspects of the manuscript.

Simulations appear to be conducted on a limited set of environments.

Results are presented primarily as single-run or deterministic comparisons.

There is no reporting of variance, confidence intervals, or statistical significance.

Parameter sensitivity and robustness are not systematically examined.

Given the stochastic nature of ACO, multiple independent runs with aggregated statistics are essential to support performance claims.

Assessment:

The experimental design demonstrates feasibility but not robustness.

Required improvements include:

Multiple-run evaluations with mean ± variance.

Statistical comparison against baseline planners.

Clear justification of parameter choices.

Inclusion of more complex or dynamic environments, if feasible.

4. Reproducibility and Transparency

The manuscript provides algorithm descriptions and pseudo-code-level explanations, which is positive. However:

Parameter values are not always fully specified.

Simulation settings (map resolution, robot dynamics, sensor assumptions) are not described in sufficient detail.

There is no indication of code or data availability.

For PLOS ONE, reproducibility is essential.

Recommendation:

The authors should provide a full parameter table and, ideally, a public repository containing simulation code or configuration files.

5. Presentation Quality and Language

The manuscript is generally well written in standard academic English. The structure is logical, and figures are relevant and clear.

Minor issues include:

Occasional grammatical inconsistencies.

Overly assertive language in the discussion that should be moderated.

Figure captions that could be more self-contained and descriptive.

6. Ethical and Publication Considerations

There are no ethical concerns related to the research content. The work does not raise issues of dual publication, misuse of data, or ethical non-compliance.

Overall Recommendation

Decision: Major Revision

This manuscript presents a competent and relevant study in mobile robot path planning and aligns with PLOS ONE’s scope. However, it currently does not meet the journal’s standards for analytical rigor and evidentiary support.

To merit publication, the authors should:

Clearly articulate the novelty of their approach relative to prior hybrid planners.

Strengthen experimental validation with statistically robust comparisons.

Improve reproducibility through detailed parameter reporting and, ideally, code sharing.

Moderate conclusions to reflect the exploratory nature of the results.

With these revisions, the manuscript could become a solid, publishable contribution to the applied robotics literature.

Reviewer #3: The authors' purpose is to present a path planning method fusing the ant colony optimization (ACO) and dynamic window approach (DWA), namely the ACO-DWA-DPP algorithm. The authors argue that the simulation results show that, compared with the traditional ant colony optimization, the improved algorithm reduces the longest path length by 41.26%-48.28%, shortens the shortest path by 10.68%-12.64%, decreases the number of iterations by 83.37%-89.51%, and reduces the number of turning points by 66.94%-81.37%.

In my opinion, the article presents some weak points. The authors need to show significant improvement over other approaches on several test scenarios to claim the advancement and novelty of their proposal.

Some points must be taken to improve the quality of the article:

* The paper contains some grammatical mistakes and typos. The whole paper must be carefully reviewed.

* The main contributions should be reviewed and stated in a clear form in the first section using bullets.

* Should have text between section title and section subtitle (e.g., between 2 and 2.1, and so on).

* Transitions from section to section should be smoother, please work on this issue to enhance readability.

* It is not clear what are the advantages, disadvantages, issues, etc. related to the implementation of the ant colony optimization in the proposal. The text should guide the reader to understand why the authors selected and implemented this approach over other methods.

* In the results, there is not a comparison concerning the state of the art. The results are insufficient to provide a valid evaluation of the proposed method.

* The test scenario presented is insufficient. The experiments are very limited to provide a valid evaluation of the proposal. The author needs to provide more scenarios to test their proposal.

*In Section 7, there is a lack of parameter setting discussion for the experimental settings. The parameter values have not been described. How would the result of your implementation vary with increasing/decreasing those values?

* The proposal must be tested on a real mobile robot. The author must present evidence of the implementation and results of the test process on the real mobile robot.

* The conclusion section should be improved to provide real useful conclusions. Not to be an overview of the previous paragraphs.

* On the conclusions, the authors need to provide solid and insightful future research directions in a separate paragraph.

* More references to path planning papers should be reviewed and included, like: “Hybrid path planning algorithm based on membrane pseudo-bacterial potential field for autonomous mobile robots,” “Mobile robot path planning using membrane evolutionary artificial potential field,” and "Mobile robot path planning using a QAPF learning algorithm for known and unknown environments".

* The references must be carefully reviewed, updated, and extended. The number of references is low for a journal paper (usually 40-50 references for journal papers), please consider the previous comments on this issue.

* A review of the state of the art is required.

**********

6. PLOS authors have the option to publish the peer review history of their article (what does this mean?). If published, this will include your full peer review and any attached files.

If you choose “no”, your identity will remain anonymous but your review may still be made public.

**Do you want your identity to be public for this peer review?**  For information about this choice, including consent withdrawal, please see our Privacy Policy.

Reviewer #1: No

Reviewer #2: No

Reviewer #3: No

**********

[NOTE: If reviewer comments were submitted as an attachment file, they will be attached to this email and accessible via the submission site. Please log into your account, locate the manuscript record, and check for the action link "View Attachments". If this link does not appear, there are no attachment files.]

To ensure your figures meet our technical requirements, please review our figure guidelines: https://journals.plos.org/plosone/s/figures

You may also use PLOS’s free figure tool, NAAS, to help you prepare publication quality figures: https://journals.plos.org/plosone/s/figures#loc-tools-for-figure-preparation.

NAAS will assess whether your figures meet our technical requirements by comparing each figure against our figure specifications.

# Author comment: Author response to Decision Letter 1

**DOI:** 10.1371/journal.pone.0340336.r002

24 Mar 2026

Dear Editor and Reviewers:

Thank you very much for taking the time to review our manuscript (Manuscript ID: PONE-D-25-67110) and for providing valuable and constructive comments. These insightful suggestions are of great significance in guiding us to improve the quality of our paper. We have carefully read and thoroughly discussed all the comments and have made comprehensive revisions to the manuscript accordingly. Below, we respond point-by-point to the comments raised by the Editor, Reviewer 1, Reviewer 2, and Reviewer 3, in that order, and provide a detailed explanation of the modifications made. All changes have been highlighted in red in the revised manuscript for your ease of review. Once again, we sincerely thank you for your hard work and thoughtful guidance.

Response to Editor

1: Please ensure that your manuscript meets PLOS ONE's style requirements, including those for file naming.

Response: Dear Editor, thank you for reviewing and providing guidance on our manuscript. In response to your comment—"Please ensure that your manuscript meets PLOS ONE's style requirements, including those for file naming"—we have conducted a comprehensive review and made necessary adjustments to the manuscript in accordance with the official PLOS ONE submission guidelines.

1、Manuscript Formatting: We have confirmed that the manuscript uses double-spacing and includes consecutive page numbers and line numbers throughout.

2、Figure and Table Formatting: All figures have been removed from the main manuscript file and are now uploaded separately as high-resolution TIFF files (resolution ≥300 dpi, meeting journal requirements).

3、File Naming: Following journal specifications, the main manuscript file has been renamed "Manuscript.docx," and the figure files have been renamed "Fig1.tif," "Fig2.tif," etc., ensuring consistency with the citations within the text.

4、Reference Formatting: The references have been uniformly formatted in the Vancouver style, ensuring compliance with ICMJE guidelines。

5、Manuscript Structure: We have verified that the manuscript contains all essential sections, including Title Page, Abstract, Introduction, Methods, Results, Discussion, Acknowledgments, and References, arranged in the order required by the journal.

All the aforementioned modifications have been highlighted in the revised manuscript. We believe that after these adjustments, the manuscript now fully complies with the submission guidelines of PLOS ONE. Should there be any further adjustments required, please do not hesitate to inform us, and we will cooperate fully.

Thank you once again for your time and valuable comments.

2: Please note that PLOS One has specific guidelines on code sharing for submissions in which author-generated code underpins the findings in the manuscript.

Response: Thank you for reviewing our manuscript and providing guidance, and for drawing our attention to PLOS ONE's important policy on code sharing. We have carefully read the guidelines you provided (https://journals.plos.org/plosone/s/materials-and-software-sharing#) and fully understand and support the journal's requirement that "all code generated by authors must be made available without restriction upon publication of the work." We recognize that this requirement is crucial for promoting the reproducibility and transparency of scientific research.

We are currently in the process of finalizing the code, improving annotations, and preparing documentation to ensure it meets best practice standards—namely, that the code is clear, well-documented, and includes necessary dependency descriptions and test data to facilitate replication and reuse by other researchers. We plan to upload the complete and properly formatted code to a public code repository that meets PLOS ONE's requirements and can issue a persistent identifier (such as a DOI), such as Zenodo or Figshare, after the paper is formally accepted but before publication. An appropriate open-source license (such as MIT or BSD) will also be adopted.

We believe that this arrangement will both fully comply with the journal's policy requirements and ensure the quality of the code ultimately shared. Thank you for your understanding and valuable comments.

3: Please note that funding information should not appear in any section or other areas of your manuscript. We will only publish funding information present in the Funding Statement section of the online submission form. Please remove any funding-related text from the manuscript.

Response: Thank you for reviewing our manuscript and providing guidance, and for pointing out the formatting requirements regarding funding information. We have carefully read your comments and fully understand PLOS ONE's policy: funding information should only be included in the Funding Statement section of the online submission form and must not appear in any part of the manuscript or other areas.

Accordingly, we have removed all funding-related text from the manuscript. The revised manuscript has been ensured to contain no funding information whatsoever.

We confirm that this modification complies with the journal's formatting requirements. Should there be any further adjustments required, please do not hesitate to inform us, and we will cooperate fully.

Thank you once again for your time and valuable comments.

4: Thank you for stating the following financial disclosure:“This work was supported by the Key Research and Development Project of the Shanxi Provincial Department of Science and Technology (202102140601015), the 2023 Scientific and Technological Innovation Project of Jinzhong National Agricultural High-tech Zone (Taigu National Science and Technology Innovation Center) (JZNGQBSGZZ004), and the Shanxi Provincial Basic Research Program (202403021222112).”Please state what role the funders took in the study. If the funders had no role, please state: "The funders had no role in study design, data collection and analysis, decision to publish, or preparation of the manuscript."If this statement is not correct you must amend it as needed.Please include this amended Role of Funder statement in your cover letter; we will change the online submission form on your behalf.

Response: Thank you for reviewing our manuscript and providing guidance. In response to your comments regarding funding information and the funders' role statement, we have revised the manuscript and cover letter as requested, as detailed below:

1、All funding-related text has been removed from the manuscript. The revised manuscript contains no funding information whatsoever, with this information now only included in the appropriate sections of the online submission system.

2、At the end of the cover letter, prior to the signature, we have added a statement listing all funding sources along with the required role statement. The specific addition reads as follows:This work was supported by the following funding projects: the Key Research and Development Project of the Shanxi Provincial Department of Science and Technology (202102140601015), the 2023 Scientific and Technological Innovation Project of Jinzhong National Agricultural High-tech Zone (Taigu National Science and Technology Innovation Center) (JZNGQBSGZZ004), and the Shanxi Provincial Basic Research Program (202403021222112). The funders had no role in study design, data collection and analysis, decision to publish, or preparation of the manuscript.

The revised manuscript and cover letter are attached to this email for your reference. Should there be any further adjustments required, please do not hesitate to inform us, and we will cooperate fully.

Thank you once again for your time and valuable comments.

5: Please ensure that you refer to Figure 5 in your text as, if accepted, production will need this reference to link the reader to the figure.

Response: Thank you for reviewing our manuscript and providing guidance. In response to your comment, we have revised the manuscript accordingly. The specific modifications are as follows: At the beginning of Section 6.2 "Path Planning Process of the Fusion Algorithm" (line 465), we have added a clear citation statement: "As shown in Fig. 5, the implementation process of the fusion algorithm is as follows:" This ensures that readers are aware of the existence of Figure 5 and its relevance to the text before reading the content. Additionally, we have reviewed all figures throughout the manuscript to ensure that each figure (Figure 1 through Figure 13) is appropriately cited in the main text.

The revised manuscript is attached to this email for your review. Should there be any further adjustments required, please do not hesitate to inform us, and we will cooperate fully. Thank you once again for your time and valuable comments.

6: Please include captions for your Supporting Information files at the end of your manuscript, and update any in-text citations to match accordingly. Please see our Supporting Information guidelines for more information:http://journals.plos.org/plosone/s/supporting-information.

Response: Thank you for reviewing our manuscript and providing guidance. In response to your comment, we have revised the Supporting Information files and the manuscript as requested, with the following specific modifications:

1.Supporting Information File Processing: The folder "1_Fig.tif" has been renamed to "S1_Fig," which contains all the figures from the manuscript. The data file "2_Data.xlsx" has been renamed to "S1_Data.xlsx," which contains the raw data from all simulation experiments.

2. Supporting Information Section Added:At the end of the manuscript (lines 858-860), we have added the Supporting Information title and descriptions:

14、Supporting Information

S1_Fig: High-resolution TIFF versions of all figures from the manuscript.

S1_Data.xlsx: Raw data from the simulation experiments reported in the manuscript.

3、In-Text Citation Added: In Section 7.2 (line 613), we have added the following citation: "The raw data supporting the quantitative analysis in Tables 2-5 are available in S1 Data."

The revised manuscript and Supporting Information files are attached to this email for your review. Should there be any further adjustments required, please do not hesitate to inform us, and we will cooperate fully. Thank you once again for your time and valuable comments.

7: Please remove your figures from within your manuscript file, leaving only the individual TIFF/EPS image files, uploaded separately. These will be automatically included in the reviewers’ PDF.

Response: Thank you for reviewing our manuscript and providing guidance. In response to your comment, we have revised the manuscript and figure files as requested, with the following specific modifications:

Manuscript File Modifications: All embedded images (Figure 1 through Figure 13) have been removed from the manuscript, with only the corresponding figure captions retained.

Preparation of Individual Figure Files:All 13 figures have been exported as TIFF format files, each meeting the journal's resolution requirements (≥300 dpi). The figure files have been named "Fig1.tif" through "Fig13.tif" and are uploaded as separate files to the submission system.

We confirm that all figures are properly cited in the main text (as Figures 1-13), and the revised manuscript format complies with PLOS ONE's submission requirements. Should there be any further adjustments required, please do not hesitate to inform us, and we will cooperate fully. Thank you once again for your time and valuable comments.

8: Thank you for providing your underlying data as Supporting Information.We note that the data set contains text or data that is not in English. Please note that PLOS is an English-language publisher, so we require data sets to be provided in English as well. Please upload an English-language version of your data set.This will also allow us to determine if your data follows PLOS standards per our Data Availability policy here: https://journals.plos.org/plosone/s/data-availability

Response: Thank you for reviewing our manuscript and providing guidance, and for drawing our attention to the language requirements for datasets. We have carefully read the PLOS Data Availability Policy you provided and fully understand that PLOS, as an English-language publisher, requires all supporting information data to also be provided in English.

In response to your comment, we have conducted a comprehensive review and revision of the underlying dataset: All non-English text within the file (including column headers, worksheet names, etc.) has been converted entirely to English. The revised dataset is now presented completely in English, ensuring compliance with PLOS's Data Availability Policy and facilitating understanding, validation, and reuse by colleagues in the international academic community.

The updated English-version dataset file (S1_Data.xlsx) has been re-uploaded to the submission system.

We confirm that the revised dataset complies with PLOS's best practices for data sharing. Should there be any further adjustments required, please do not hesitate to inform us, and we will cooperate fully.

Thank you once again for your time and valuable comments.

9: If the reviewer comments include a recommendation to cite specific previously published works, please review and evaluate these publications to determine whether they are relevant and should be cited. There is no requirement to cite these works unless the editor has indicated otherwise.

Response:

Thank you for reviewing our manuscript and providing guidance. We fully understand and have complied with your comment regarding: "If the reviewers' comments include suggestions to cite specific published literature, please review and evaluate whether these references are relevant and should be cited."

During this revision process, we have carefully examined all the literature suggested for citation by the reviewers and have conducted our evaluation based on the following principles:

Relevance:Assessing whether the literature is directly related to the methodology, results, or discussion of this study;

Necessity: Determining whether citing the literature would enhance the academic integrity of the paper or provide important background for readers;

Avoidance of Redundant Citations: Ensuring that non-essential references are not added merely because they were "suggested for citation."

We confirm that the final version of the reference list has followed the editor's instructions and has not added any unnecessary references due to "suggested citations." Should there be any further adjustments required, please do not hesitate to inform us.

Thank you once again for your time and valuable comments.

10: Please review your reference list to ensure that it is complete and correct. If you have cited papers that have been retracted, please include the rationale for doing so in the manuscript text, or remove these references and replace them with relevant current references. Any changes to the reference list should be mentioned in the rebuttal letter that accompanies your revised manuscript. If you need to cite a retracted article, indicate the article’s retracted status in the References list and also include a citation and full reference for the retraction notice.

Response:

Thank you for reviewing our manuscript and providing guidance. In response to your comment, we have conducted a comprehensive and meticulous verification of the entire reference list in the manuscript. The specific tasks completed are as follows:

Completeness Verification:We have checked one by one all citations in the main text (23 in total) against the reference list at the end of the manuscript (numbered [1] to [23]), ensuring a one-to-one correspondence with no omissions or redundant entries.

Accuracy Check:We have verified the author names, titles, journal names, years, volume/issue numbers, and page ranges for each reference. Individual formatting inconsistencies (such as journal name abbreviation standards) have been corrected to ensure that all entries comply with the journal's formatting requirements.

Retraction Status Verification:We have searched all references in the list through the CrossRef and Retraction Watch databases. Upon verification, none of the cited papers have been

Attachment Submitted filename: Response to Reviewers.docx

# Peer review: Decision Letter 1

**DOI:** 10.1371/journal.pone.0340336.r003

**Authors:** Ravneil Nand

14 Apr 2026

PONE-D-25-67110R1

Path Planning for Mobile Robots by Fusing Ant Colony Optimization and Dynamic Window Approach

PLOS One

Dear Dr. Guozhu,

Thank you for submitting your manuscript to PLOS ONE. After careful consideration, we feel that it has merit but does not fully meet PLOS ONE’s publication criteria as it currently stands. Therefore, we invite you to submit a revised version of the manuscript that addresses the points raised during the review process.

Please submit your revised manuscript by May 29 2026 11:59PM. If you will need more time than this to complete your revisions, please reply to this message or contact the journal office at plosone@plos.org. When you're ready to submit your revision, log on to https://www.editorialmanager.com/pone/ and select the 'Submissions Needing Revision' folder to locate your manuscript file.

Please include the following items when submitting your revised manuscript:

- A letter that responds to each point raised by the academic editor and reviewer(s). You should upload this letter as a separate file labeled 'Response to Reviewers'.
- A marked-up copy of your manuscript that highlights changes made to the original version. You should upload this as a separate file labeled 'Revised Manuscript with Track Changes'.
- An unmarked version of your revised paper without tracked changes. You should upload this as a separate file labeled 'Manuscript'.

If you would like to make changes to your financial disclosure, please include your updated statement in your cover letter. Guidelines for resubmitting your figure files are available below the reviewer comments at the end of this letter.

If applicable, we recommend that you deposit your laboratory protocols in protocols.io to enhance the reproducibility of your results. Protocols.io assigns your protocol its own identifier (DOI) so that it can be cited independently in the future. For instructions see: https://journals.plos.org/plosone/s/submission-guidelines#loc-laboratory-protocols. Additionally, PLOS ONE offers an option for publishing peer-reviewed Lab Protocol articles, which describe protocols hosted on protocols.io. Read more information on sharing protocols at https://plos.org/protocols?utm_medium=editorial-email&utm_source=authorletters&utm_campaign=protocols.

As the corresponding author, your ORCID iD is verified in the submission system and will appear in the published article. PLOS supports the use of ORCID, and we encourage all coauthors to register for an ORCID iD and use it as well. Please encourage your coauthors to verify their ORCID iD within the submission system before final acceptance, as unverified ORCID iDs will not appear in the published article. *Only*  the individual author can complete the verification step; PLOS staff *cannot*  verify ORCID iDs on behalf of authors.

We look forward to receiving your revised manuscript.

Kind regards,

Ravneil Nand

Academic Editor

PLOS One

Journal Requirements:

1. If the reviewer comments include a recommendation to cite specific previously published works, please review and evaluate these publications to determine whether they are relevant and should be cited. There is no requirement to cite these works unless the editor has indicated otherwise.

2. Please review your reference list to ensure that it is complete and correct. If you have cited papers that have been retracted, please include the rationale for doing so in the manuscript text, or remove these references and replace them with relevant current references. Any changes to the reference list should be mentioned in the rebuttal letter that accompanies your revised manuscript. If you need to cite a retracted article, indicate the article’s retracted status in the References list and also include a citation and full reference for the retraction notice.

Additional Editor Comments:

Please share code via online repository.

[Note: HTML markup is below. Please do not edit.]

Reviewer's Responses to Questions

**Comments to the Author**

1. If the authors have adequately addressed your comments raised in a previous round of review and you feel that this manuscript is now acceptable for publication, you may indicate that here to bypass the “Comments to the Author” section, enter your conflict of interest statement in the “Confidential to Editor” section, and submit your "Accept" recommendation.

Reviewer #1: All comments have been addressed

Reviewer #2: (No Response)

**********

2. Is the manuscript technically sound, and do the data support the conclusions?

The manuscript must describe a technically sound piece of scientific research with data that supports the conclusions. Experiments must have been conducted rigorously, with appropriate controls, replication, and sample sizes. The conclusions must be drawn appropriately based on the data presented.

Reviewer #1: (No Response)

Reviewer #2: Partly

**********

3. Has the statistical analysis been performed appropriately and rigorously?

Reviewer #1: (No Response)

Reviewer #2: No

**********

4. Have the authors made all data underlying the findings in their manuscript fully available?

The PLOS Data policy requires authors to make all data underlying the findings described in their manuscript fully available without restriction, with rare exception (please refer to the Data Availability Statement in the manuscript PDF file). The data should be provided as part of the manuscript or its supporting information, or deposited to a public repository. For example, in addition to summary statistics, the data points behind means, medians and variance measures should be available. If there are restrictions on publicly sharing data—e.g. participant privacy or use of data from a third party—those must be specified.

Reviewer #1: (No Response)

Reviewer #2: No

**********

5. Is the manuscript presented in an intelligible fashion and written in standard English?

PLOS ONE does not copyedit accepted manuscripts, so the language in submitted articles must be clear, correct, and unambiguous. Any typographical or grammatical errors should be corrected at revision, so please note any specific errors here.

Reviewer #1: (No Response)

Reviewer #2: Yes

**********

6. Review Comments to the Author

Please use the space provided to explain your answers to the questions above. You may also include additional comments for the author, including concerns about dual publication, research ethics, or publication ethics. (Please upload your review as an attachment if it exceeds 20,000 characters)

Reviewer #1: accept~~~~~~~~~~~~~~~~~~~~~~~~~~~~~~~~~~~~~~~~~~~accept~~~~~~~~~~~~~~~~~~~~~~~~~~~~~~~~~~~~~~~~~~~accept~~~~~~~~~~~~~~~~~~~~~~~~~~~~~~~~~~~~~~~~~~~accept~~~~~~~~~~~~~~~~~~~~~~~~~~~~~~~~~~~~~~~~~~~

Reviewer #2: Thank you for the opportunity to review this revised manuscript.

1. Summary of the Manuscript: The manuscript proposes a hybrid path-planning framework (ACO-DWA-DPP) that integrates an improved Ant Colony Optimization (ACO) algorithm for global path planning with an enhanced Dynamic Window Approach (DWA) for local trajectory planning and dynamic obstacle avoidance. The study introduces several modifications including a potential-field-based heuristic function, a pheromone reward–punishment mechanism, an adaptive evaporation strategy, and a dynamic collision-risk coefficient within the DWA evaluation function. Simulation experiments are conducted to evaluate performance across several environments, and comparisons are made against conventional ACO-based algorithms.

The revised manuscript presents a technically sound algorithmic framework for mobile robot path planning. The fusion of global ACO optimisation with local DWA trajectory control is conceptually appropriate and consistent with established practices in robotics path-planning research.

2. Technical Soundness and Validity of the Study: I have read the rebuttal letter and the revised text carefully and I acknowledge that the authors have made a genuine and constructive effort to address the concerns raised in the first round of review. Several important corrections have been made, and the manuscript is more clearly organised and more precisely written than the original submission. These improvements are appreciated.

3. Novelty and Contribution:

The revised manuscript now clearly identifies the principal contributions of the proposed approach in the Introduction (lines 83–99). These include:

A bidirectional closed-loop feedback mechanism between the global ACO planner and the local DWA planner.

A dynamic collision-risk model incorporating relative distance, velocity, and acceleration of obstacles.

A connectivity-based path smoothing strategy applied after algorithm convergence to remove redundant turning points.

The explicit articulation of these contributions significantly improves the clarity of the paper and helps distinguish the proposed approach from existing hybrid path-planning frameworks.

I am able to confirm that the following issues from the prior review have been satisfactorily resolved. The sign convention in the dynamic collision-risk evaluation function (Equation 25) has been corrected so that trajectories with higher collision risk receive lower evaluation scores. The path smoothing step is now described as a post-convergence operation that is applied once after the ACO algorithm has reached a final path, which resolves the ambiguity regarding its effect on computational cost. The claim of real-time obstacle detection has been appropriately contextualised as referring to the algorithm's capacity to respond to obstacle information within an idealised simulation environment, rather than to the output of a physical sensing system. The three principal contributions of the framework are now stated explicitly in numbered form in the Introduction. Transitional paragraphs have been added between sections, and the analysis of traditional ACO limitations has been strengthened.

Despite these improvements, a number of concerns remain that must be addressed before the manuscript can be accepted. I present these below, ordered by scientific significance, with specific reference to the relevant sections and line numbers in the revised manuscript.

Comment 1: Statistical Reporting (Major). The experimental results in Tables 2 through 5 report mean performance values across 30 independent runs but provide no measures of dispersion and no inferential statistical analysis. For stochastic population-based algorithms such as ACO, mean-only reporting is insufficient to characterise the reliability of the observed performance differences. Without standard deviations or equivalent dispersion measures, it is not possible for the reader to determine whether the reported improvements represent a consistent advantage or fall within the natural run-to-run variability of the algorithm.

This concern is most acute for the comparisons presented in Table 3, where the proposed algorithm's improvements in shortest path length relative to IACO and AACO range from 4.2 to 6.1 percent. Differences of this magnitude in stochastic optimization cannot be interpreted without supporting statistical evidence. I request that the authors add standard deviation values to all entries in Tables 2 through 5 and apply a non-parametric pairwise significance test, such as the Wilcoxon signed-rank test, to each algorithm comparison. The test statistic and the corresponding p-value should be reported for each pairwise comparison. This revision is necessary for the conclusions drawn in Section 7.2 to be scientifically defensible.

Comment 2: Dimensional Error in Equation (22) (Major). Equation (22) defines the relative velocity v_rel between the mobile robot and a dynamic obstacle. In the second term under the square root, the expression 'y_r sin(theta_r)' appears, where y_r is the y-coordinate of the robot's position. In the context of a velocity magnitude computation, this term is dimensionally inconsistent: y_r has units of length (metres) whereas the expression requires a velocity component (metres per second). The physically correct term is 'v_r sin(theta_r)', where v_r is the linear velocity of the robot and theta_r is its heading angle with respect to the obstacle.

This is not a minor typographic error. The relative velocity v_rel is the direct input to the collision time estimate T_collision in Equation (23), which in turn determines the dynamic collision-risk coefficient D_dynamic in Equation (24). D_dynamic is one of the three claimed primary contributions of this work and is central to the improved performance reported in Section 7.3. The authors must correct Equation (22), recompute all affected derived quantities, and confirm that the simulation results presented in the manuscript are based on the corrected formulation.

Comment 3: Geometric Validity of the Segment Intersection Test in Equation (14) (Major). Section 3.4 describes the path secondary optimization procedure, which prunes redundant turning points by testing whether direct connections between non-adjacent path nodes intersect obstacle boundaries. The intersection criterion is expressed in Equation (14), where the sign of the scalar cross product of two directional vectors is used to classify intersection (positive), parallelism or overlap (zero), and non-intersection (negative).

This criterion is mathematically incorrect for the purpose of bounded segment intersection. The sign of the cross product of two directional vectors determines only the angular relationship between the two supporting lines; it does not establish whether the bounded segments share a common point. A rigorous segment intersection test requires computing four cross products and verifying that the endpoints of each segment straddle the supporting line of the other segment. The test as formulated in Equation (14) will classify non-intersecting segments as intersecting whenever the extensions of their supporting lines cross at an angle, and will misclassify the intersection status of segments that meet near their midpoints. Since the correctness of the path pruning operation depends entirely on this test, its invalidity is a substantive concern.

I request that the authors replace Equation (14) with a geometrically correct segment intersection algorithm, such as the standard cross-product straddling test, or provide a clear and rigorous mathematical justification demonstrating that the simplified criterion is sufficient within the specific constraints of their grid-based obstacle representation.

Comment 4: Underspecified Parameters in Equation (12) (Moderate). Equation (12) expresses the adaptive pheromone evaporation coefficient rho as a function of the current iteration k, the maximum iteration count K, an initial value rho_1, a maximum bound rho_max, and a minimum bound rho_min. Table 1 lists rho as a single fixed value of 0.6. Neither rho_max nor rho_min is specified anywhere in the manuscript. The initial value rho_1 = 0.6 is stated in the text at lines 287 to 288, but the relationship between this initial value and the entry in Table 1 is not explained. In the absence of rho_max and rho_min, Equation (12) cannot be independently reproduced. I request that the authors specify these values, together with a description of the effective range of rho across the iterative process, in Table 1 or in the parameter description that follows the equation.

Comment 5: Experimental Comparison with the Most Closely Related Prior Work (Moderate). The manuscript identifies Reference [22] (Xue et al., 2025: A hybrid ant colony optimization and dynamic window method for real-time navigation of unmanned surface vehicles, Sensors) as a prior hybrid ACO-DWA approach. This is the most closely related antecedent to the proposed method in the reference list. The paper is cited in Section 6.1 in the context of the bidirectional feedback architecture but is not included as a comparison algorithm in the experimental evaluation. The absence of this comparison weakens the empirical support for the novelty claims, since it is not possible to determine from the experiments whether the performance advantages reported in Section 7 arise from the proposed contributions specifically or from the general class of ACO-DWA fusion approaches.

I request that the authors either include a quantitative experimental comparison with the method of Xue et al. (2025) or provide a detailed technical analysis in Section 6.1 documenting the specific algorithmic differences that distinguish the proposed framework from that work and explaining why those differences are expected to yield superior performance in the tested environments.

Comment 6: Dynamic Obstacle Validation Scenario (Moderate). The evaluation of dynamic obstacle avoidance performance in Section 7.3 is conducted with one dynamic obstacle alongside multiple static obstacles. The introduction motivates the research with reference to complex dynamic environments, and the primary claimed contribution at the local planning layer is a collision-risk model that incorporates obstacle acceleration. A single dynamic obstacle is insufficient to stress-test this model or to demonstrate the bidirectional feedback mechanism, which is described as being triggered when the local planner detects that the global path is blocked. I recommend that the authors either extend the simulation to include at least two simultaneously moving obstacles with distinct velocity and acceleration profiles, or acknowledge this limitation explicitly in the Conclusion section and identify multi-obstacle dynamic scenarios as a specific and prioritised future work direction.

Comment 7: Kinematic Validity of DWA for the Ackermann Model (Moderate). Section 4.1 specifies that the study examines the Ackermann steering mechanism. The Dynamic Window Approach was originally formulated by Fox, Burgard, and Thrun (1997) for differential-drive robots, for which linear and angular velocity are independently controllable. The Ackermann model imposes different non-holonomic constraints: the turning radius is a function of the wheelbase and steering angle, and the robot cannot rotate in place. The velocity sampling constraints stated in Equations (17) through (19) assume a symmetric, independently controllable velocity space that may not accurately represent the physically reachable set of an Ackermann vehicle. The manuscript provides no justification for applying the standard DWA velocity sampling framework to this platform. The authors should include a brief technical justification or cite prior work that validates this adaptation. If simplifying assumptions have been made, for example treating the Ackermann vehicle as a kinematically equivalent unicycle model for the purposes of velocity sampling, these should be stated explicitly.

Comment 8: Computational Complexity and Runtime (Moderate). The manuscript reports iteration count as a proxy for computational efficiency, and Table 4 shows substantial reductions in iteration count for the proposed algorithm relative to the baselines. However, iteration count and wall-clock computation time are not equivalent. The proposed improvements introduce several computationally intensive additions relative to the traditional ACO, including a multi-component heuristic function, a three-tiered pheromone reward-punishment computation, an adaptive evaporation coefficient evaluated at each iteration, and a post-convergence path pruning step. The DWA layer similarly introduces a per-step collision-risk computation involving relative velocity and acceleration estimation. Without reporting actual execution times or providing an analytical complexity discussion, it cannot be determined whether the reduction in iteration count translates to a net reduction in total computation time. I request that the authors report average wall-clock execution times for each algorithm, measured under identical hardware and software conditions, or provide an analytical discussion of the computational complexity trade-offs.

Comment 9: Parameter Sensitivity Results (Recommended Enhancement). Lines 550 through 556 state that a sensitivity analysis was conducted over ranges of alpha, beta, and rho using a controlled variable method, and that the algorithm demonstrated robustness within these ranges. This is a qualitative assertion. The results of the sensitivity analysis are not presented in the manuscript, and no quantitative evidence is provided to support the claim of robustness. I recommend that the authors include a supplementary table or figure reporting the variation in at least one key performance metric across the tested parameter ranges. This would provide empirical support for the robustness claim and would serve as useful practical guidance for readers seeking to apply the method to different problem environments.

Comment 10: Algorithm Scalability (Recommended Enhancement). All simulation experiments are conducted on grid maps of 20 by 20 and 30 by 30 nodes. These scales are modest relative to the operational environments referenced in the motivating applications of intelligent warehouse logistics and autonomous vehicle navigation. The time complexity of ACO grows with the number of nodes and ants, and the addition of a bidirectional feedback mechanism introduces computational coupling between the global and local planning layers. The manuscript does not discuss how the proposed framework is expected to perform at larger scales. I encourage the authors to include a brief scalability discussion in the Conclusion or Future Work sections, acknowledging the computational limitations of the current implementation and proposing how these might be addressed in future research.

In summary, the following revisions are required before the manuscript can be accepted for publication:

1. Supplement Tables 2 through 5 with standard deviation values for all reported metrics. Apply non-parametric pairwise significance testing to each algorithm comparison and report the resulting test statistics and p-values.

2. Correct the dimensional error in Equation (22) by replacing y_r with v_r in the relative velocity expression, and reconfirm that all simulation results are based on the corrected formulation.

3. Correct or provide a rigorous mathematical justification for the segment intersection criterion in Equation (14).

4. Specify rho_max and rho_min in Table 1 or in the parameter description accompanying Equation (12).

5.Conduct a full audit of all in-text equation cross-references and correct those that do not correspond to the rendered equation numbers.

6. Provide either a quantitative experimental comparison with Xue et al. (2025, Reference [22]) or a detailed technical comparison in Section 6.1.

7. Expand the dynamic obstacle validation to include at least two simultaneously moving obstacles, or explicitly acknowledge this limitation in the Conclusion.

8. Replace the unresolved citation placeholder '[citations]' at line 460 with appropriate references.

9. Include a technical justification for applying the standard DWA velocity sampling constraints to the Ackermann kinematic model.

10.Report average wall-clock execution times or provide a computational complexity analysis for the proposed and baseline algorithms.

11. Correct the Figure 8 caption to read 'Comparison of Path Planning in Complex Environments.'

12. Correct the double-period error at line 24 of the abstract.

13. Revise the Acknowledgments section to remove the reference to a listed co-author.

The following enhancements are recommended but not mandatory for acceptance:

14. Include quantitative results from the parameter sensitivity analysis to support the robustness claim at lines 550 through 556.

15. Include a scalability discussion in the Conclusion or Future Work sections.

**********

7. PLOS authors have the option to publish the peer review history of their article (what does this mean?). If published, this will include your full peer review and any attached files.

If you choose “no”, your identity will remain anonymous but your review may still be made public.

**Do you want your identity to be public for this peer review?**  For information about this choice, including consent withdrawal, please see our Privacy Policy.

Reviewer #1: No

Reviewer #2: No

**********

[NOTE: If reviewer comments were submitted as an attachment file, they will be attached to this email and accessible via the submission site. Please log into your account, locate the manuscript record, and check for the action link "View Attachments". If this link does not appear, there are no attachment files.]

To ensure your figures meet our technical requirements, please review our figure guidelines: https://journals.plos.org/plosone/s/figures

You may also use PLOS’s free figure tool, NAAS, to help you prepare publication quality figures: https://journals.plos.org/plosone/s/figures#loc-tools-for-figure-preparation.

NAAS will assess whether your figures meet our technical requirements by comparing each figure against our figure specifications.

# Author comment: Author response to Decision Letter 2

**DOI:** 10.1371/journal.pone.0340336.r004

14 May 2026

1.Supplement Tables 2 through 5 with standard deviation values for all reported metrics. Apply non-parametric pairwise significance testing to each algorithm comparison and report the resulting test statistics and p-values.

Response: Thank you for this important comment. We agree that mean-only reporting is insufficient for stochastic population-based algorithms. In the revised manuscript, Tables 2–5 have been supplemented with standard deviation values, and the results are now reported as mean ± standard deviation. In addition, we conducted pairwise Wilcoxon signed-rank tests between the proposed algorithm and each baseline algorithm using the 30 independent runs. To avoid making the main text overly lengthy, the test statistic W and the corresponding p-value for each comparison are reported in S2_Table. We also added a brief description of the statistical testing procedure in Section 7.

Thank you once again for your time and valuable comments.

2.Correct the dimensional error in Equation (22) by replacing yr with vr in the relative velocity expression, and reconfirm that all simulation results are based on the corrected formulation.

Response: Thank you for identifying this dimensional inconsistency in Equation (22). We agree that the original term involving yrsin(θr) was inappropriate because yr represents a position coordinate rather than a velocity component. In the revised manuscript, we corrected this term to vrsin(θr), so that the relative velocity expression is dimensionally consistent. We also rechecked the subsequent calculation of the collision time and the dynamic collision-risk coefficient based on the corrected formulation. The revised Equation (22) now uses the velocity components of the mobile robot and the obstacle, which makes the dynamic risk evaluation physically consistent.

Thank you once again for your time and valuable comments.

3.Correct or provide a rigorous mathematical justification for the segment intersection criterion in Equation (14).

Response: Thank you very much for this insightful comment. We agree that the original formulation based on a single cross-product sign was insufficient for rigorous bounded line-segment intersection detection. Following the reviewer’s suggestion, we have revised the segment intersection criterion in Section 3.4 by adopting the standard cross-product orientation test combined with overlap verification.

Specifically, the original simplified criterion has been replaced with a geometrically rigorous intersection determination method based on four orientation values:

o1=cross(A,B,C)  o2=cross(A,B,D)o3=cross(C,D,A)  o4=cross(C,D,B)

The intersection condition is now expressed as:

AB∩CD≠∅⟺o1o2≤0∧o3o4≤0∧BAB,CD=1

whereBAB,CD=1 indicates that the projections of the two line segments overlap in both the x- and y-directions.

In addition, Equation (13) and the related textual descriptions in Section 3.4 have been revised accordingly to ensure mathematical correctness and consistency with standard computational geometry methods.

These revisions improve the rigor and reliability of the path-pruning process without affecting the overall conclusions of the study.

Thank you once again for your time and valuable comments.

4.Specify rho_max and rho_min in Table 1 or in the parameter description accompanying Equation (12).

Response:Thank you for pointing out this omission. We agree that the parameters in the adaptive pheromone evaporation coefficient should be explicitly specified to ensure the reproducibility of Equation (12). In the revised manuscript, we clarified that the original value “Rho = 0.6” in Table 1 represents the initial pheromone evaporation coefficient, denoted as ρ1, rather than a fixed evaporation coefficient. We also added the lower and upper bounds of the adaptive evaporation coefficient, namely ρmin=0.1 and ρmax=0.9, in both Section 3.3 and Table 1. This revision makes the effective range of ρ during the iterative process clear and reproducible.

Thank you once again for your time and valuable comments.

5.Conduct a full audit of all in-text equation cross-references and correct those that do not correspond to the rendered equation numbers.

Response: Thank you for this helpful comment. We have conducted a full audit of all in-text equation cross-references in the manuscript. The incorrect references in Section 3.1 have been corrected: the distance calculation is now referred to as Equation (7) instead of Equation (11), and the improved heuristic function is now referred to as Equation (9) instead of Equation (13). In addition, we removed the duplicated “(12):” text following the reference to Equation (12) and standardized the wording around Equation (13) in Section 3.4. All remaining in-text equation references were checked and confirmed to be consistent with the rendered equation numbers.

Thank you once again for your time and valuable comments.

6.Provide either a quantitative experimental comparison with Xue et al. (2025, Reference [22]) or a detailed technical comparison in Section 6.1.

Response: Thank you for this valuable suggestion. We agree that the relationship between the proposed method and the closely related hybrid ACO-DWA method by Xue et al. [22] should be clarified. Since the method of Xue et al. was developed for unmanned surface vehicles, whereas this study focuses on mobile robots with Ackermann steering constraints in grid-based ground environments, a direct quantitative comparison under the current experimental setting would not be fully consistent. Therefore, following the reviewer’s suggestion, we added a detailed technical comparison in Section 6.1.

In the revised manuscript, we clarified that existing ACO-DWA fusion methods, such as Xue et al. [22], generally use the ACO-generated global path as one-way guidance for DWA-based local obstacle avoidance. In contrast, the proposed ACO-DWA-DPP framework introduces a bidirectional closed-loop feedback mechanism, in which newly detected obstacles and path blockage information from the DWA layer are fed back to the ACO layer for pheromone redistribution and global path re-optimization. We also highlighted the additional differences, including the acceleration-aware dynamic collision risk coefficient and the post-convergence connectivity-based secondary optimization. These revisions clarify the novelty of the proposed framework compared with existing ACO-DWA fusion approaches.

Thank you once again for your time and valuable comments.

7.Expand the dynamic obstacle validation to include at least two simultaneously moving obstacles, or explicitly acknowledge this limitation in the Conclusion.

Response: Thank you for this valuable suggestion. We agree that a single dynamic obstacle is not sufficient to fully stress-test the proposed acceleration-aware collision-risk model or the bidirectional feedback mechanism in highly dynamic environments. Due to the current experimental scope, we are unable to add a complete multi-dynamic-obstacle experiment in this revision. Therefore, following the reviewer’s suggestion, we have explicitly acknowledged this limitation in the revised manuscript.

Specifically, we added a clarification in Section 7.3 that the current validation mainly evaluates the algorithm’s response to one moving obstacle combined with multiple unknown static obstacles. We also added a limitation statement in the Conclusion, noting that more complex scenarios involving multiple simultaneously moving obstacles with different velocities, accelerations, and motion directions will be prioritized in future work. This revision more accurately describes the scope of the current validation and avoids overgeneralizing the dynamic obstacle avoidance results.

Thank you once again for your time and valuable comments.

8.Replace the unresolved citation placeholder '[citations]' at line 460 with appropriate references.

Response: Thank you for pointing this out. We have corrected the unresolved citation placeholder in Section 6.1. The original placeholder “[citations]” has been removed and replaced with the specific reference to Xue et al. [22], which is the most closely related ACO-DWA fusion method discussed in the manuscript. The revised text now properly cites the relevant prior work when discussing existing ACO-DWA fusion approaches.

Thank you once again for your time and valuable comments.

9. Include a technical justification for applying the standard DWA velocity sampling constraints to the Ackermann kinematic model.

Response: Thank you for this important comment. We agree that the use of the standard DWA velocity sampling framework for an Ackermann mobile robot should be clarified. In the revised manuscript, we added a brief technical explanation after Equation (16), stating that the Ackermann mobile robot is simplified as a kinematically equivalent unicycle model at the vehicle reference point during the DWA-based local planning stage. Under this representation, v denotes the forward velocity and ω denotes the heading-rate of the robot, rather than an independently controllable in-place rotational command. We also clarified that infeasible motions such as in-place rotation are excluded during velocity sampling. In addition, we revised the wording in Section 4.2 by replacing the original reference to a “differential-drive robot” with the Ackermann mobile robot under the equivalent unicycle representation.

Thank you once again for your time and valuable comments.

10.Report average wall-clock execution times or provide a computational complexity analysis for the proposed and baseline algorithms.

Response: Thank you for this helpful comment. We agree that iteration count alone cannot fully represent the overall computational efficiency of the algorithm. To avoid introducing unsupported runtime data, we added a concise computational complexity discussion in Section 7.2. In the revised manuscript, we clarified that the proposed improvements, including the improved heuristic function, pheromone reward-punishment strategy, adaptive evaporation mechanism, path secondary optimization, and dynamic collision-risk evaluation, increase the computational burden of a single planning cycle to some extent. We also explained that the substantial reduction in convergence iterations reported in Table 4 helps compensate for this additional overhead, and that the path secondary optimization is performed only after ACO convergence rather than during every iteration.

Thank you once again for your time and valuable comments.

11.Correct the Figure 8 caption to read 'Comparison of Path Planning in Complex Environments.'

Response: Thank you for pointing out this caption error. We have corrected the caption of Figure 8 in the revised manuscript. The original caption “Comparison of Path Planning in General Environments” has been revised to “Comparison of Path Planning in Complex Environments” to make it consistent with the corresponding experimental scenario described in Section 7.1.

Thank you once again for your time and valuable comments.

12.Correct the double-period error at line 24 of the abstract.

Response: Thank you for pointing out this typographical error. We have corrected the double-period error in the Abstract by replacing “robots..” with “robots.” in the revised manuscript.

Thank you once again for your time and valuable comments.

13.Revise the Acknowledgments section to remove the reference to a listed co-author.

Response: Thank you for pointing this out. We have revised the Acknowledgments section to remove the reference to Prof. Guozhu Song, who is already listed as a co-author of the manuscript. The revised Acknowledgments section now only thanks the laboratory colleagues and fellow students for their support and discussions.

Thank you once again for your time and valuable comments.

14.Include quantitative results from the parameter sensitivity analysis to support the robustness claim at lines 550 through 556.

Response: Thank you for this constructive suggestion. We agree that quantitative parameter sensitivity results would provide stronger support for the robustness claim. Since a complete quantitative sensitivity analysis over all parameter combinations was not included in the current experimental design, we revised the related statement in the manuscript to avoid overgeneralizing the robustness conclusion. Specifically, we changed the description of the parameter sensitivity analysis to a preliminary parameter adjustment using the controlled variable method and added that a more systematic quantitative sensitivity analysis will be conducted in future work. This revision makes the discussion more accurate and avoids unsupported claims.

Thank you once again for your time and valuable comments.

15.Include a scalability discussion in the Conclusion or Future Work sections.

Response:Thank you for this helpful suggestion. We agree that the scalability of the proposed framework should be discussed, since the current simulations are conducted on 20 × 20 and 30 × 30 grid maps. In the revised manuscript, we added a scalability discussion in the Future Work section. We clarified that larger maps may increase the computational cost of ant colony search and the interaction overhead between the global and local planning layers. We also stated that future work will evaluate the proposed framework on larger-scale grid maps and explore efficiency-improvement strategies such as hierarchical planning, map decomposition, and adaptive search-space reduction.

We sincerely thank the Editor and Reviewers again for their careful evaluation of our manuscript and for the constructive comments and suggestions. These comments have helped us improve the rigor, clarity, and completeness of the manuscript. We have revised the manuscript carefully according to all comments and hope that the revised version satisfactorily addresses the concerns raised. We appreciate your time and consideration.

Sincerely,

The Authors

Attachment Submitted filename: Response_to_Reviewers_auresp_2.docx

# Peer review: Decision Letter 2

**DOI:** 10.1371/journal.pone.0340336.r005

**Authors:** Nezir Aydin

2 Jun 2026

Path Planning for Mobile Robots by Fusing Ant Colony Optimization and Dynamic Window Approach

PONE-D-25-67110R2

Dear Dr. Guozhu,

We’re pleased to inform you that your manuscript has been judged scientifically suitable for publication and will be formally accepted for publication once it meets all outstanding technical requirements.

Within one week, you’ll receive an e-mail detailing the required amendments. When these have been addressed, you’ll receive a formal acceptance letter and your manuscript will be scheduled for publication.

An invoice will be generated when your article is formally accepted. Please note, if your institution has a publishing partnership with PLOS and your article meets the relevant criteria, all or part of your publication costs will be covered. Please make sure your user information is up-to-date by logging into Editorial Manager at Editorial Manager® and clicking the ‘Update My Information' link at the top of the page. For questions related to billing, please contact billing support.

If your institution or institutions have a press office, please notify them about your upcoming paper to help maximize its impact. If they’ll be preparing press materials, please inform our press team as soon as possible -- no later than 48 hours after receiving the formal acceptance. Your manuscript will remain under strict press embargo until 2 pm Eastern Time on the date of publication. For more information, please contact onepress@plos.org.

Kind regards,

Nezir Aydin, Ph.D.

Academic Editor

PLOS One

# Editor report: Acceptance letter

**DOI:** 10.1371/journal.pone.0340336.r006

**Authors:** Nezir Aydin

PONE-D-25-67110R2

PLOS One

Dear Dr. Song,

I'm pleased to inform you that your manuscript has been deemed suitable for publication in PLOS One. Congratulations! Your manuscript is now being handed over to our production team.

At this stage, our production department will prepare your paper for publication. This includes ensuring the following:

* All references, tables, and figures are properly cited

* All relevant supporting information is included in the manuscript submission,

* There are no issues that prevent the paper from being properly typeset

You will receive further instructions from the production team, including instructions on how to review your proof when it is ready. Please keep in mind that we are working through a large volume of accepted articles, so please give us a few days to review your paper and let you know the next and final steps.

Lastly, if your institution or institutions have a press office, please let them know about your upcoming paper now to help maximize its impact. If they'll be preparing press materials, please inform our press team within the next 48 hours. Your manuscript will remain under strict press embargo until 2 pm Eastern Time on the date of publication. For more information, please contact onepress@plos.org.

You will receive an invoice from PLOS for your publication fee after your manuscript has reached the completed accept phase. If you receive an email requesting payment before acceptance or for any other service, this may be a phishing scheme. Learn how to identify phishing emails and protect your accounts at https://explore.plos.org/phishing.

If we can help with anything else, please email us at customercare@plos.org.

Thank you for submitting your work to PLOS ONE and supporting open access.

Kind regards,

PLOS ONE Editorial Office Staff

on behalf of

Professor Nezir Aydin

Academic Editor

PLOS One
